# Gene Expression Profiling during Conidiation in the Rice Blast Pathogen *Magnaporthe oryzae*

**DOI:** 10.1371/journal.pone.0043202

**Published:** 2012-08-21

**Authors:** Kyoung Su Kim, Yong-Hwan Lee

**Affiliations:** 1 Department of Applied Biology, College of Agriculture and Life Sciences, Research Institute for Agriculture and Life Sciences, Kangwon National University, Chuncheon, Korea; 2 Department of Agricultural Biotechnology, Center for Fungal Genetic Resources, Center for Fungal Pathogenesis, Center for Agricultural Biomaterials, Plant Genomics and Breeding Institute, and Research Institute for Agriculture and Life Sciences, Seoul National University, Seoul, Korea; Universidad Pública de Navarra, Spain

## Abstract

Conidiation of phytopathogenic fungi is a key developmental process that plays a central role in their life cycles and in epidemics. However, there is little information on conidiation-induced molecular changes in the rice blast fungus *Magnaporthe oryzae*. As a first step to understand conidiogenesis in this fungus, we measured genome-wide gene expression profiles during conidiation using a whole genome oligonucleotide microarray. At a two-fold expression difference, approximately 4.42% and 4.08% of genes were upregulated and downregulated, respectively, during conidiation. The differentially expressed genes were functionally categorized by gene ontology (GO) term analysis, which demonstrated that the gene set encoded proteins that function in metabolism, cell wall biosynthesis, transcription, and molecule transport. To define the events of the complicated process of conidiogenesis, another set of microarray experiments was performed using a deletion mutant for *MoHOX2*, a stage-specific transcriptional regulator essential for conidial formation, which was expressed *de novo* in a conidiation-specific manner in *M. oryzae*. Gene expression profiles were compared between the wild-type and the Δ*Mohox2* mutant during conidiation. This analysis defined a common gene set that was upregulated in the wild-type and downregulated in the Δ*Mohox2* mutant during conidiation; this gene set is expected to include conidiation-related downstream genes of *MoHOX2*. We identified several hundred genes that are differentially-expressed during conidiation; our results serve as an important resource for understanding the conidiation, a process in *M. oryzae,* which is critical for disease development.

## Introduction

Microbes have evolved the capacity to complete a series of complicated developmental processes in association with surrounding environments as strategies for survival and dispersal. This is obviously true for the rice blast fungus *Magnaporthe oryzae*, a pathogen of considerable importance for both global rice cultivation and understanding fungal biology. The rice blast fungus causes up to 15% annual yield loss, which is enough rice to feed about 60 million people [1]. *M. oryzae* is a polycyclic pathogen capable of many disease cycles in favorable weather within a single crop growing season, which may explain why the pathogen is so destructive to rice in certain geographical areas. Spatial dissemination and disease severity leading to the epidemic of rice blast rely on production of conidia by multiple rounds of asexual reproduction. A new round of conidiation from infected tissues takes as few as 3–5 days, depending on the level of compatibility between the pathogen and the host, as well as environmental conditions [2]. Therefore, understanding the molecular mechanisms of conidiation in *M. oryzae* enables the development of better strategies for the control of fungal crop disease.

Conidiogenesis involves a series of morphological events. Like most fungi, *M. oryzae* must undergo hyphal growth, through which hyphae become developmentally competent, a few days before conidiation begins. Then the fungus develops conidiophores, specialized aerial hyphae that are less branched and modestly determinate in growth, which are distinguishable from vegetative aerial hyphae. After conidiophore development, three-celled conidia are arrayed at the tip of a conidiophore in a sympodial fashion. Swelling at the apex of a conidiophore gives rise to the first conidium. The active apical tip of the conidiophores moves to the side to form the next conidium. Rounds of this event produce several conidia, mostly three to five, borne sympodially on a conidiophore. Conidiation occurs, provided that competent hyphae are exposed to aeration although other factors such as humidity and light have a quantitative effect on conidiation. Conversely, conidiation can be blocked by maintaining hyphae in submerged growth. These observations indicate that aeration is a key inducer of conidiation. Molecular mechanisms governing conidiation have been characterized in detail for the two model organisms *Aspergillus nidulans* [3], [4] and *Neurospora crassa* [5], but not for a phytopathogenic fungus, *M. oryzae,* despite its essential roles in pathogenic development.

Mutagenesis-mediated approaches have been employed to identify genes regulating conidiation. These efforts have identified several loci regulating conidial shapes. For example, a mutation at the spore morphology *(SMO*) locus produces a variety of abnormal shapes of conidia that are defective in the development of appressorium, a specialized infection structure. However, conidial production by *smo^-^* mutants is unaffected [6]. Disruption of a gene designated acropetal (*ACR1*) produces conidia with a head-to-tail array instead of sympodially arrayed conidia; the mutant (*acr1^-^*) fails to cause disease due to a defect in attachment to hydrophobic surfaces [7]. Several other loci have been defined as conidiation mutants (and thus were named *con*), which were obtained by random mutagenesis, but most of these loci have not been characterized [8]. A recent characterization of one of the loci, *Con7*, revealed that it encodes a member of the zinc-finger transcription factor family that is required not only for conidial shape, but also for appressorium formation; however, *Con7* does not affect conidial production [9]. These lines of evidence indicate that genes for conidial morphology are often linked to pathogenicity through their effect on pre-infection stages. In addition to genes involved in conidial shapes, recent studies have characterized two transcription factors, *COS1* [10] and *MoHOX2* [11], [12] that are necessary for conidiophore development and conidial formation, respectively; therefore, these genes will likely be of great value in the molecular and genetic dissection of differentiation pathways during conidiogenesis.

*M. oryzae* has emerged as a model for studying fungal development and pathogenicity due to its economic significance and its amenability to molecular technologies. The availability of the genome sequences of both rice and the fungal pathogen has facilitated a systematic, functional analysis of the genomes, which provides a more integrated view of the cellular roles of both known and novel genes in fungal development and pathogenesis [13], [14]. Considerable progress has been made in understanding the nature of appressorial development and pathogenicity in *M. oryzae* through genome-wide approaches including microarray analysis [15], [16], [17]. However, less is understood of the molecular events during conidiation on a whole genome-scale. In this study, we employed an Agilent whole-genome oligonucleotide chip to establish large-scale expression profiling of the *M. oryzae* transcriptome during conidiation, resulting in the identification of global changes in mRNA abundance; several hundred conidiation-induced genes were identified. Furthermore, a subset of genes that are regulated by the transcriptional regulator MoHOX2 was determined from the conidiation-induced gene set. Therefore, this study provides a foundation for studying conidiation-associated genes in *M. oryzae* and gaining insights into the molecular events controlling conidiation, which could contribute to the development of strategies for crop protection.

## Results

### Culture Conditions for Studying *M. oryzae* Conidiation

**Figure 1:**
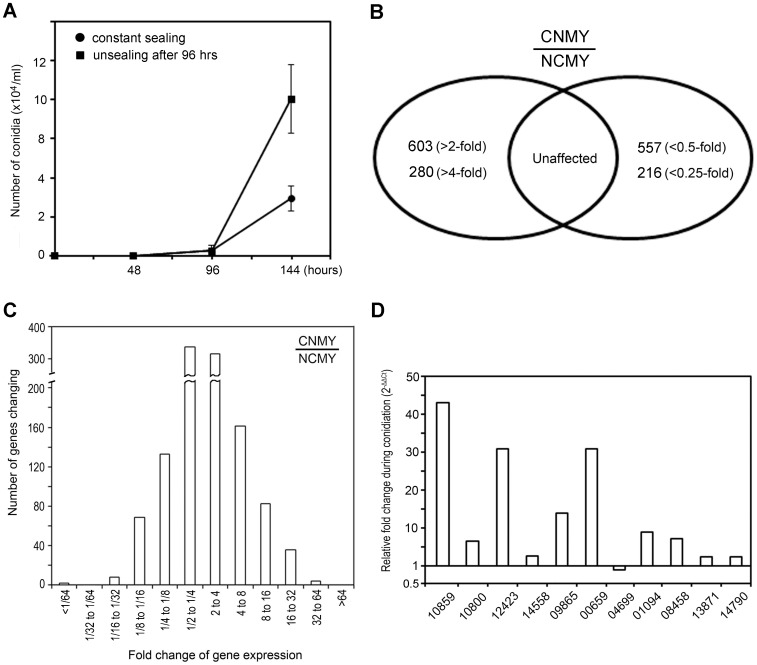
The genome-wide analysis of changes in mRNA abundance during conidiation. (**A**). Measurement of the conidial density of *M. oryzae* on polycarbonate membrane-laid OMA plates at the indicated times. (**B**). The number of genes induced (left) or repressed (right) during conidiation based on the comparison of RNA levels of non-conidiating mycelia (NCMY) with conidiating mycelia (CNMY) of *M. oryzae*. (**C**). The number of genes induced (right) or repressed (left) with fold change values during conidiation of *M. oryzae*. (**D**). Validation of the microarray data by qRT-PCR. Transcript levels of each gene in CNMY were normalized to *β*-tubulin and expressed as relative values, with 1 corresponding to the NCMY. Each of five-digit number on the x-axis indicates the MGG locus number.

To obtain biological materials to investigate the genes that are differentially expressed during conidiation, *M. oryzae* was cultured on polycarbonate membranes laid upon oat meal agar (OMA) plates to prevent hyphal infiltration into substances, thereby facilitating the recovery of fungal tissues. Rather than inoculating conidia on OMA, fragmented hyphae were used as inocula because the Δ*Mohox2* mutant used in subsequent experiments has a defect in conidial production. Conidiation curves showed that the wild-type produced few conidia on the sealed filter-laid plates 76–96 h after inoculation (Figure 1A). However, increased conidiation was observed 96 h after incubation on sealed plates. Moreover, a dramatic increase in conidiation was observed after the seal was removed from the plates after 96 h. Based on these findings, sealed cultures grown for 80 h on polycarbonate membranes laid on OMA were used as non-conidiating mycelia (NCMY) of the wild-type whereas cultures aerated for additional 48 h were used as conidiating mycelia (CNMY).

### Identification of Conidiation-induced Genes from a Microarray Study

Microarray analyses were performed with RNAs extracted from NCMY and CNMY of isolate KJ201 and the Agilent *M. oryzae* Genechips to identify conidiation-induced genes. The error control strategies applied to the microarray analysis included replicate hybridizations with dye-swap experiments, use of RNAs pooled from three independent cultures, and validation of genes identified from microarray analysis by qRT-PCR (described below). Comparing mRNA levels between NCMY and CNMY revealed that many genes were differentially expressed during conidiation. This analysis identified 603 unique genes that were induced (>2-fold) in CNMY compared to NCMY, among which 280 showed a greater than four-fold increase (Figure 1B). A total of 557 genes were downregulated by more than two-fold in CNMY compared to NCMY; 216 genes showed expression decreases of more than four-fold (Figure 1C) shows the number of genes with significant fold changes in NCMY versus CNMY. The genes induced and repressed during conidiation are provided in Tables S3 and S4, respectively. Because we were interested in identifying conidiation-induced genes that are also regulated by the homeobox transcription factor MoHOX2 (described later), qRT-PCR was used to validate genes induced during conidiation based on the microarray data (Figure 1D). Thus, qRT-PCR was performed against 11 genes using the second biological replicate pools of samples on the filter-laid OMA plates incubated for 72–80 h (pre-conidiation) and for an additional 48 h aeration (post-conidiation). Data from qRT-PCR confirmed that most genes were significantly induced during conidiation (Figure 1D). Induction levels of genes quantitatively varied due to technical issues between the microarray and qRT-PCR data [18], as well as variation in sampling times and culture coniditions that can influence conidiation as shown (Figure 1A). For example, the induction levels of genes MGG10859 and MGG10800 were 16.9-fold and 11.5-fold on microarray data, respectively, but 43.0-fold and 6.5-fold in qRT-PCR assay, respectively (Figure 1D). Only 1 (MGG04699) out of 11genes appeared to be a false-positive, for which GeneChip and qRT-PCR indicated 3.9-fold and 0.9-fold ratios in NCMY versus CNMY, respectively; therefore, the qRT-PCR data suggest that there is no significant change in transcriptional expression of the gene. The high degree of validation that we observed with qRT-PCR supports the transcriptional changes in genes observed by microarrays.

**Figure 2:**
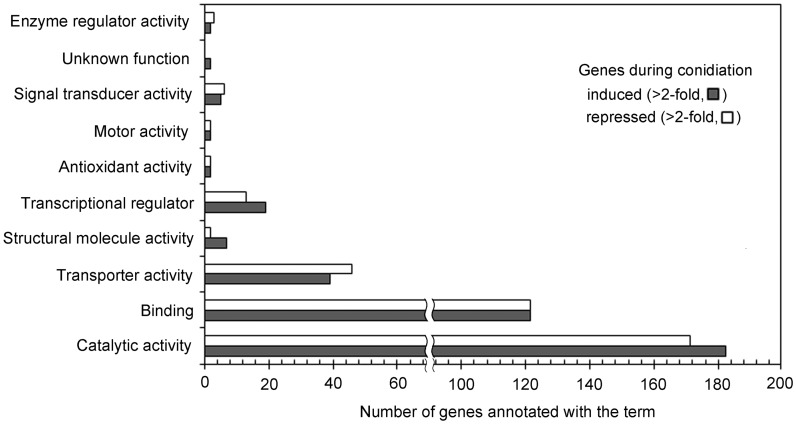
Molecular functions of the genes induced and repressed during conidiation of *M. oryzae* at a two-fold expression threshold based on the Gene Ontology (GO) terms.

To understand the functional relationships between genes differentially expressed at a two-fold threshold during conidiation, enrichment of specific GO terms was analyzed (http://www.geneontology.org/) (Figure 2). The GO term “molecular functions-catalytic activity” was the most predominant in both the induced and repressed genes; this category includes the activities of oxidoreductase, transferase, ligase, hydrolase, and helicase. The second most common GO term was binding, which includes binding of nucleic acid, carbohydrate, cofactor, and nucleotides. GO terms such as transcriptional regulator, structural molecule activity, and catalytic activity were slightly more prevalent in induced genes than in repressed genes during conidiation. As expected, these results confirm that conidiation is accomplished via changes in gene expression.

### Conidiation-specific Expression of *MoHOX2*

**Figure 3:**
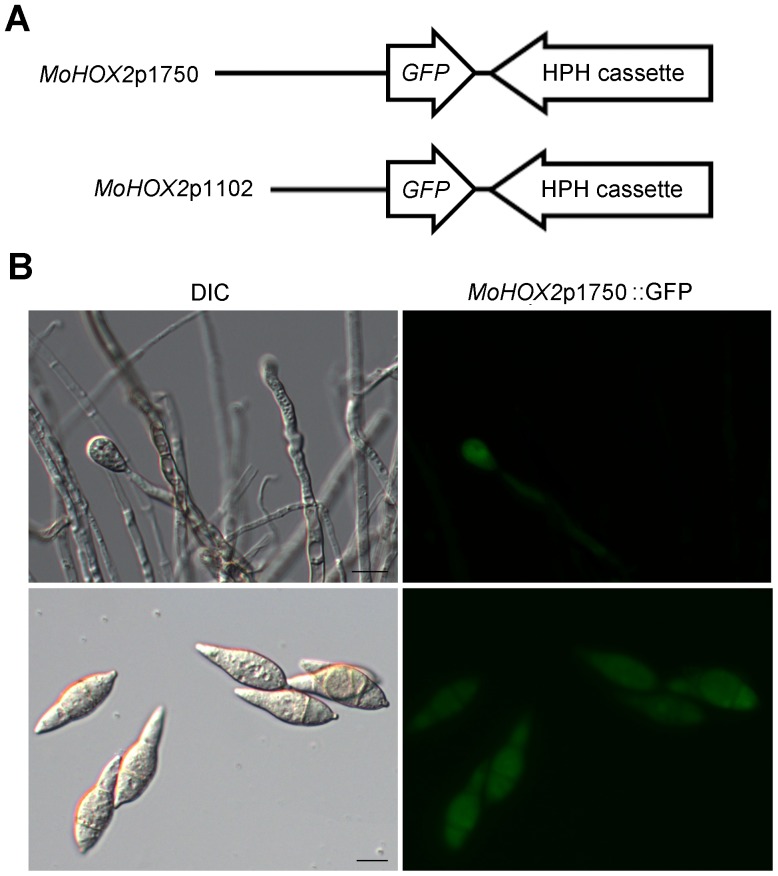
Microscopic analysis of *M. oryzae* transformants expressing a transgene of GFP behind the *MoHOX2* promoter. (**A**). Schematic of the expression constructs. The 1,750 bp and 1,102 bp upstream region of the *MoHOX2* gene were fused to GFP followed by an HPH cassette in pIGPAPA to generate *MoHOX2p*1750::GFP::HPH and *MoHOX2p*1102::GFP::HPH, respectively. (**B**). Microscopic observation of transformants expressing the indicated construct. Differential interference contrast (DIC) images (left) and GFP fluorescence images (right) are shown in conidiating hyphae (upper panel) and detached conidia (lower panel). Scale bars represent 10 µm.

We previously showed that the homeobox transcription factor MoHOX2 plays an essential role in *M. oryzae* conidiation; the deletion mutant Δ*Mohox2* causes a complete failure of asexual reproduction, where conidiophore development is normal, but no conidia form [11]. To better understand when *MoHOX2* is expressed in developmental pathways, a fusion of its promoter (1750 bp upstream of the translation start site) to the green fluorescent protein (GFP) was transformed into *M. oryzae* (Figure 3A). The *MoHOX2*p1750::GFP transgene indicated the spatial and temporal patterns of the expression of the *MoHOX2* gene during the establishment of asexual reproduction (Figure 3B). Examination of seven independent transformants by fluorescence microscopy revealed that GFP expression was absent in hyphae, but present in the conidiophore bearing an immature conidium and mature conidia (Figure 3B). As shown (Figure 3), the fluorescence intensity of GFP was slightly higher in conidia than in conidiophores. An extensive examination revealed that a very faint spot of GFP was visible only in terminal swellings of a few aerial hyphae, which presumably represent conidial initials; however, no detectable GFP was observed in the length of the hyphae (data not shown). In addition, a truncated *MoHOX2* promoter fused to GFP (*MoHOX2*p1102::GFP) also showed the same expression pattern as the *MoHOX2*p1750::GFP, suggesting that a functional promoter for *MoHOX2* expression exists within 1102 bp from its open reading frame (Figure 3A). These observations indicate that *MoHOX2* is transcriptionally regulated in an early stage of conidial formation, which is consistent with the functional role of *MoHOX2* in conidiation.

### Transcriptional Changes in a Deletion Mutant for *MoHOX2*

**Figure 4:**
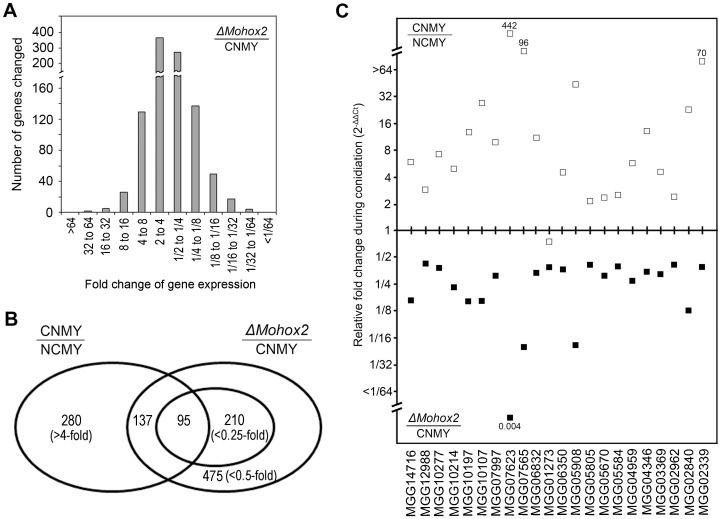
The genome-wide analysis of changes in mRNA abundance in the Δ*Mohox2* mutant during conidiation. (**A**). The number of genes induced (left) or repressed (right) with fold change values by comparing RNA levels in wild type with those in the Δ*Mohox2* mutant during conidiation. (**B**). The number of genes induced in wild-type and repressed in the Δ*Mohox2* mutant during conidiation. (**C**). Validation of microarray data by qRT-PCR. Graph shows the transcript levels of each gene on the same x-axis in the wild-type (white squares) and in the Δ*Mohox2* mutant (black squares) during conidiation.

Based on both the timing of expression and the functional role of MoHOX2, another set of microarray assays was performed to establish the set of genes regulated by MoHOX2 during conidiation. In the second microarray, mRNAs from conidiating cultures of the wild-type and Δ*Mohox2* deletion mutant were compared; mRNAs were prepared under the same conditions as the first array. The data from the second microarray revealed that a large number of genes were differentially expressed in the Δ*Mohox2* strain compared to wild type (Figure 4A). Overall, a total of 475 genes (Table S5) were downregulated (>2-fold) with 210 genes showing a greater than four-fold decrease, based on *t* test analysis (*P*  = 0.05); 524 genes (Table S6) were upregulated (>2-fold) with 161 genes increasing more than four-fold in the Δ*Mohox2* mutant. To determine the core set of conidiation genes that are regulated by MoHOX2, we searched for overlapping genes that are significantly induced in the wild-type and repressed in the Δ*Mohox2* mutant during conidiation by comparing the data from the first and second microarrays. As illustrated in the Venn diagram (Figure 4B), this comparison revealed that 137 of 280 genes that were induced >4-fold during conidiation in the wild-type were significantly downregulated by >2-fold in the mutant; 95 genes were downregulated by >4-fold in the mutant. Therefore, these genes may include downstream targets of MoHOX2, whose functions are associated with establishing conidiation. The descriptions of the 95 genes that were induced in the wild-type and repressed in the Δ*Mohox2* mutant during conidiation are listed (Table S7). The predicted functions of these gene products, based on InterPro domain assignment, include regulation (e.g., transcription factors, phosphatidylinositol kinase), metabolism (e.g., peptidases, hydroxylases, synthetases, transporters, myosin), and cell wall (e.g., sterol methyltransferase, chitin-bind protein).

To further validate the microarray results, we performed qRT-PCR with the third biological replicate pools of the wild-type and the Δ*Mohox2* mutant. Samples were obtained at 72 h (pre-conidiation) and 120 h (post-conidiation) from the filter-laid OMA plates. The results of qRT-PCR were consistent with the microarray data; 21 out of 22 genes in the subset of microarray data were confirmed to be significantly induced in the wild-type and repressed in the Δ*Mohox2* mutant during conidiation (Figure 4C). The one exception was MGG01273, which showed significant repression (0.38-fold) in the mutant, but no induction (0.85-fold) in the wild-type by qRT-PCR analysis; this gene is predicted to encode a hypothetical protein without any known structural domains. Of the 21 genes confirmed by qRT-PCR, MGG07623 was the most highly induced (442-fold) in the wild-type and showed almost no expression (0.004-fold) in the mutant during conidiation (Figure 4C); this gene encodes a small hypothetical protein (122 amino acids) with a chitin-binding domain (IPR001002). Therefore, these assays have identified a subset of genes whose expression during conidiation is dependent on the transcription factor MoHOX2.

### Transcriptional Expression of Conidiation-related Genes

**Figure 5:**
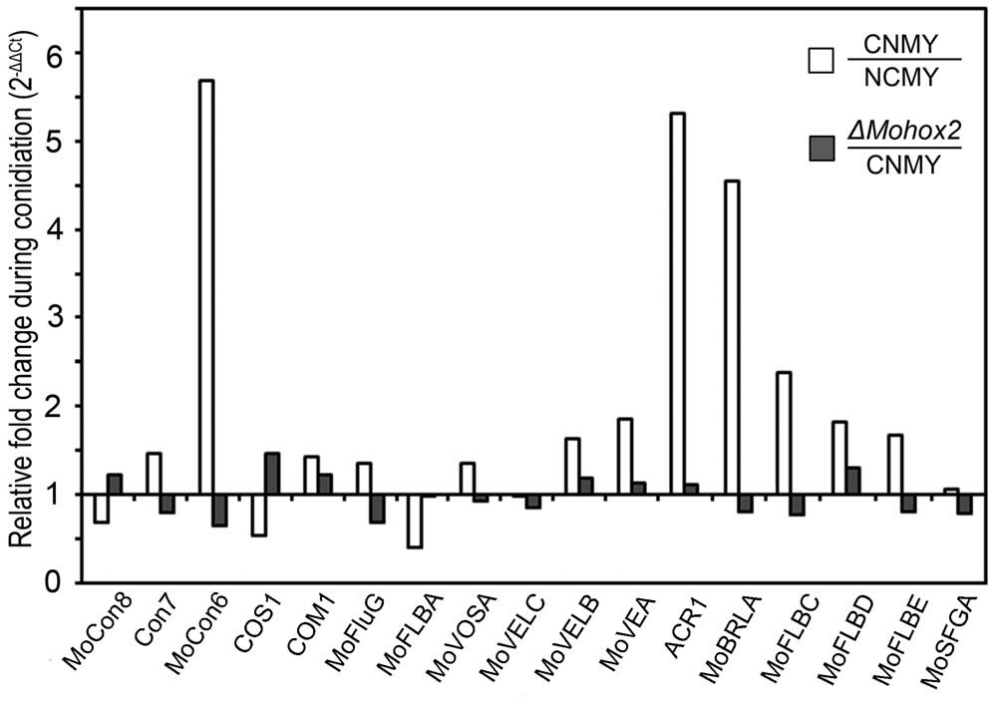
Measurements of the transcripts obtained by qRT-PCR in the wild-type (open) and in the Δ*Mohox2* mutant (gray) during conidiation. The MGG locus number and description of the genes are also shown in Table 1.

To better understand the transcriptional regulation of conidiation-related *M. oryzae* genes and the orthologs of conidiation-related genes in other fungi, we measured their expression levels both in the wild-type and the Δ*Mohox2* mutant during conidiation. The descriptions of the tested genes are shown (Table 1). The expression of *MoCON6, ACR1, MoBRLA, and MoFLBC* was significantly upregulated in wild type, but not in the Δ*Mohox2* mutant during conidiation (Figure 5), indicating that their expression is developmentally regulated in a MoHOX2-independent manner during conidiation. However, the expression of an *M. oryzae* homolog (*MoFLBA*) of *flbA* from *Aspergillus nidulans* was significantly downregulated in the wild-type and was unaffected in the Δ*Mohox2* mutant during conidiation. Expressions of *COS1* and *COM1* were slightly, but not significantly, changed in both the wild-type and the Δ*Mohox2* mutant, which is consistent with previous observations that these genes are constitutively expressed during development [19], [10]. The *M. oryzae* gene *MoVOSA* was not significantly altered during conidiation (Figure 5), but the ortholog *VosA* is highly expressed during conidiation *A. nidulans* [20]; this discrepancy might indicate that morphological differences between fungal species are reflected in variation in gene expression and function. Consistent with this idea, Δ*Movosa* deletion mutants have no defects in conidiation (unpublished data), but *VosA* is a key regulator in *A. nidulans* conidiation [20].

**Table 1:** *Magnaporthe oryzae* genes used in qRT-PCR.

Gene	Locus	Description	Reference
*MoCON8*	MGG00513	Hypothetical protein, ortholog to *con-8* in *N. crassa*	(Madi *et al*., 1994)
*CON7*	MGG05287	C_2_H_2_ zinc finger transcription factor, abnormal conidia	(Odenbach *et al.,* 2007)
*MoCON6*	MGG02246	Hypothetical protein, ortholog to *con-6* in *N. crassa*	(Madi *et al*., 1994)
*COS1*	MGG03394	C_2_H_2_ zinc finger transcription factor, conidiophores stalk-less	(Zhou *et al*., 2009)
*COM1*	MGG01215	Putative transcription factor, abnormal conidia	(Yang *et al*., 2010)
*MoFLUG*	MGG02538	Putative glutamine synthetase, ortholog to *fluG* in *A. nidulans*	(Lee & Adams, 1994)
*MoFLBA*	MGG14517	Putative regulator of G protein signaling, ortholog to *flbA* in *A. nidulans*	(Wieser *et al*., 1994)
*MoVOSA*	MGG00617	Hypothetical protein, ortholog to *VosA* in *A. nidulans*	(Ni & Yu, 2007)
*MoVELC*	MGG14719	Hypothetical protein, ortholog to *VelC* in *A. nidulans*	(Ni & Yu, 2007)
*MoVELB*	MGG01620	Hypothetical protein, ortholog to *VelB* in *A. nidulans*	(*Bayram et al*., 2008)
*MoVEA*	MGG08556	Hypothetical protein, ortholog to *VeA* in *A. nidulans*	(Kafer, 1965, Kim *et al*., 2002)
*ACR1*	MGG09847	Hypothetical protein with a glutamine-rich domain, acropetal conidia	(Lau & Hamer, 1998)
*MoBRLA*	MGG12958	Hypothetical protein, ortholog to *BrlA* in *A. nidulans*	(Adams *et al*. 1998)
*MoFLBC*	MGG04699	Hypothetical protein, ortholog to *FlbC* in *A. nidulans*	(Adams *et al*. 1998)
*MoFLBD*	MGG06898	Hypothetical protein, ortholog to *FlbD* in *A. nidulans*	(Adams *et al*. 1998)
*MoFLBE*	MGG01731	Hypothetical protein, ortholog to *FlbE* in *A. nidulans*	(Adams *et al*. 1998)
*MoSFGA*	MGG07368	Hypothetical protein, ortholog to *SfgA* in *A. nidulans*	(Adams *et al*. 1998)

## Discussion

Although several genes that play a role in conidiation have been described, this is the first report of genome-wide expression profiles during conidiation. As a step toward understanding the events of conidiation in the phytopathogenic fungus *M. oryzae*, we established transcriptional expression profiles on the basis of mRNAs levels of conidiating tissue relative to non-conidiating tissue using microarrays. Our study revealed that 603 and 557 genes were significantly induced and repressed, respectively, at a two-fold expression difference during conidiation. This corresponds to 4.42% and 4.08%, respectively, of the 13,666 genes on the GeneChip [21]. Generally expected, many of the highly induced genes should play significant roles in conidiation; in future studies the gene functions can be revealed by examining the conidiation phenotype of loss-of-function mutants. Genes with less than two-fold expression changes may also have important physiological functions because many genes have multiple roles during the life cycle of *M. oryzae*. The numbers of genes that we have defined in this study may not represent all of the transcriptional changes necessary for conidiation because other environmental factors are able to influence signaling pathways for conidiogenesis. Conversely, differences in aging and respiration between the pre- and post-conidiation samples may also contribute to changes in mRNA abundance that are unrelated to conidiation. Despite these caveats, several independent studies support our conidiation microarray data. The expression of *TRE1* (MGG01261), which encodes trehalase involved in trehalose metabolism, always increases during conidiation [22]; *ACR1*, which plays role in conidiation, is highly expressed during conidiation [7]. In addition, we recently found that two conidiation-induced genes (MGG05343 and MGG09263), predicted to encode putative Zn(II)2Cys6 transcription factors, were significantly expressed during conidiation, and deletion mutants for each gene were defective in conidiation, but mycelial growth of mutants was normal (unpublished data). MGG05343 is one of the genes repressed during conidiation in the Mohox2 mutant, supporting the validity and robustness of our data. Future studies of *M. oryzae* transformants expressing a fluorescent protein behind the promoters of various conidiation genes will help illustrate a temporal hierarchy of the transcriptional regulation of genes at different stages of conidiation. It is expected that conidiation-induced genes may contain a subset of genes that are involved in the development of aerial hyphae. Aerial hyphae are thought to be physiologically distinct from conidiophores because genes only expressed in aerial hyphae have been identified [23].

Based on the predicted functions of the conidiation-induced genes, these genes (except for 198 genes predicted to encode hypothetical proteins) appear to play diverse roles including metabolic, regulatory, and structural functions. For example, structural-class genes include membrane proteins (MGG07565, 09604, 01094, 02884, 04378, 13535, 02692, 14731, 05176, and 08126) and cell wall-related proteins (MGG07997, 07623, 04858, 00659, 01368, 13013, and 04346). MGG04346 encodes a sterol 24-c-methyltransferase that is required for the biosynthesis of ergosterol, the major membrane sterol in most fungi; this gene showed a >20-fold induction during conidiation, suggesting important roles for sterol synthesis and modification in conidiation as well as in pathogenicity [24], Many genes (>20) encoding transporters for amino acids, organic acids, and sugars are induced; such transporters are likely to support conidial development. The majority of conidiation-induced genes represent a diverse array of enzymes; therefore these expression changes are indicative of the dramatic metabolic and physiological changes that occur during conidiation. Unlike conidiation-induced transporters, it is thought that many transporters (>35) induced in appressoria are related to pathogenicity by exporting host-specific and host non-specific metabolites into host cells to suppress the host defense responses or to facilitate disease [16], [25].

In addition to the genome-wide analysis of changes in mRNA abundance during conidiation, we identified a MoHOX2-regulated subset of conidiation-induced genes, as the deletion mutant for the homeodomain transcription factor MoHOX2 blocks conidial development [11]. This investigation revealed that 137 and 95 out of conidiation-induced genes were significantly downregulated in the Δ*Mohox2* mutant by >2-fold and >4-fold, respectively. Predicted functions of this subset of genes include roles in regulation (e.g., transcription factor and phosphatidylinositol kinase), cell wall architecture (e.g., chitin-binding protein, cell wall protein, and sterol 24-c-methyltransferase), metabolism (e.g., cytochrome P450, peroxisome, oxidoreductase, hydrolase, and peptidase), signaling (e.g., G-protein coupled receptor-like protein), stress response (e.g., heat shock protein and stress response protein), and movement (e.g., transporter and myosin). Such diverse classes of MoHOX2-regulated genes suggest that MoHOX2-mediated conidiation is more complex than originally expected. Because *MoHOX2* is highly expressed *de novo* in conidiophores and conidia, and its deletion mutant is defective only in conidial development, we believe that the strategy to compare two array data sets, upregulated genes in the wild type and downregulated genes in the *MoHOX2* deletion mutant during conidiation, is an effective way to find MoHOX2-regulated genes that are required for conidial development. Future studies will employ a combinatorial approach using a chromatin immunoprecipitation assay, such as ChIP-ChIP, with this expression profiling data to determine which genes are directly regulated by MoHOX2 and to identify regulatory sequences for target genes of MoHOX2 at the genome level. Such an approach will provide a unique perspective for dissecting MoHOX2-mediated conidiation pathways.

One of the most highly induced genes during conidiation was MGG07623, which encodes a predicted protein with one chitin-binding domain. Notably, the transcription of this gene is dependent upon *MoHOX2*, suggesting that the failure of the Δ*Mohox2* mutant in conidial production may in part be due to its inability to form cell wall components. In support of this hypothesis, the expression of another gene MGG07997 involved in cell wall biogenesis was also found to be MoHOX2-dependent during conidiation. Previously, *CBP1*, which has two chitin-binding domains and one chitin deacetylase domain, was identified as a germ-tube specific gene from an *M. oryzae* cDNA library; CBP1 plays a role in plant surface recognition during appressorial differentiation [26]. Based on the stage-specific discovery of the two chitin-binding domain-containing genes, a genome-wide search with a chitin-binding domain was performed. This identified a total of 26 genes in *M. oryzae* genome, which vary in terms of their number of chitin-binding domains and enzymatic activity (data not shown). Because one (MGG07623) of the 26 genes was expressed in a MoHOX2-dependent manner during conidiation, we are currently working to uncover the functional role of this gene family in the context of fungal development and pathogenicity.

It is notable that a G protein-coupled receptor (GPCR; MGG07565), which represents one of the largest families of signaling molecules that communicate across membranes, is highly induced during conidiation in a MoHOX2-dependent manner (Table S7). It is tempting to suggest that the GPCR is transcriptionally regulated and that its signal transduction is critical for conidiation. It was previously noted that there are about 76 GPCR proteins in *M. oryzae* [27]. MGG07565 is one of the PTH11 receptors in the GPCR family that contain an extracellular cysteine-rich EGF-like domain at its amino terminus. While the exact mechanisms for GPCRs in *M. oryzae* have not been demonstrated, PTH11 has been implicated in appressorial development and pathogenicity, acting upstream of the cAMP-dependent pathway [28]. As in many fungi, several lines of evidence suggest a variety of signals associated with the culmination of conidiogenesis in *M. oryzae,* which may invoke separate receptor-mediated signaling pathways in the development of conidiation in response to diverse stimuli as a survival strategy. Our previous study suggested that *MoHOX2* expression is dependent on the upstream signaling pathways of adenylate cyclase and phospholipase C as its expression is significantly downregulated in the Δ*mac1* and Δ*Moplc1* mutants, but not in the MAPKKK mutant Δ*mck1* or the MAPK Δ*pmk1* [11]. Consistent with this idea, expression of many conidiation-related genes was not significantly affected in the Δ*Mohox2* mutant although many of them are known to function in conidiation (Figure 5, Table 1). Therefore, it would be interesting to investigate the receptor-initiated crosstalk between the signaling pathways that culminate in conidiogenesis.

In summary, we have presented the changes in transcriptome that occur during conidiation and a subset of conidiation genes that are regulated by the conidiation-specific transcription factor MoHOX2 in the pathogenic fungus *M. oryzae*. Future analyses of these genes may help elucidate how each gene contributes to conidiation and why the contribution has evolved into pathogenic fitness of *M. oryzae*.

## Materials and Methods

### Culture Conditions and RNA Extractions

The wild type strain KJ201 and the Δ*Mohox2* deletion mutant of *M. oryzae*, which were obtained from the Center for Fungal Genetic Resources (CFGR; http://cfgr.snu.ac.kr), were used in this study. The isolates were routinely grown on V8 agar (8% V8 juice (v/v), 1.5% agar (w/v), pH 6.0) or oatmeal agar medium (OMA; 5% oatmeal [w/v], 2% agar [(w/v)) at 25°C in constant light to promote conidial development.

Total RNA was isolated from non-condiating mycelia and conidiating mycelia using the Easy-Spin total RNA extraction kit (Intron Biotechnology, Seongnam, Korea); total RNA was treated with DNase prior to use in microarray or qRT-PCR. For harvesting non-conidiating mycelia, agar plugs (5 mm in diameter) were obtained from the actively growing edge of oatmeal agar (OMA) plates, inoculated into liquid complete medium, and incubated at 28°C on a 250 rpm shaker for 4 days. Then the mycelia mats were washed with sterilized water on two-layer gauze, fragmented into small pieces, and passed through the gauze by pouring over equal amounts of sterilized water. Then an equal volume of the hyphal suspension (200 µl) was spread onto a 0.4 µm pore polycarbonate membrane laid on the surface of the OMA plates. The plates were sealed with plastic wrap, and incubated at 25°C with constant light. The whole tissue grown on the polycarbonate membrane was harvested 72–96 h after inocultion by scraping the surface with a razor blade. A portion of the harvested tissues was vortexed with water and filtered through Miracloth (CalBiochem, La Jolla, CA). The filtrate was examined under a microscope to ensure nonconidiation culture. Conidiating mycelia were obtained from polycarbonate OMA culture, which was aerated for an additional 48 h after removal of the seal on the plates.

### Generation of *M. oryzae* Transformants Expressing GFP Behind the *MoHOX2* Promoter

To generate transformants expressing GFP behind the *MoHOX2* promoter, the double-joint PCR method was applied [29]. A fragment corresponding to approximately 1.7 kb upstream of the MoHOX2 ORF was amplified with primers H2PF1/H2PR (Table S2). A 2.8 kb length of promoter-less GFP and hygromycin phosphotransferase gene (HPH) cassette was amplified with primers GHF and GHR from pIGPAPA [30]. The two fragments were fused using PCR with primers H2PF1/GHR. Protoplasts from KJ201 strain were directly transformed with the purified PCR product (*MoHOX2*p1750::GFP::HPH). Hygromycin-resistant transformants were selected on TB3 (0.3% yeast extract, 0.3% casamino acid, 1% glucose, 20% sucrose) media supplemented with 200 mg/ml hygromycin B (Calbiochem, San Diego, CA, USA). PCR and Southern blot analysis were conducted using genomic DNA from selected transformants to confirm integration of the fusion gene.

### Microarray Hybridization and Data Analysis

The quality of the total RNA (1 µg) pooled from the three biological replicates was evaluated on the 2100 Bioanalyzer (Agilent Technologies, Inc., Wilmington, DE, USA). The synthesis of cRNA incorporated with cyanine 3 or cyanine 5-labeled dCTP was performed using Agilent’s Low RNA Input Linear Amplication Kit (Agilent Technologies), purified with the cRNA Cleanup Module (Agilent Technologies), and quantified on the ND-1000 spectrophotometer (NanoDrop Technologies, Inc., Wilmington, DE) according to the manufacturer’s instructions. The cRNA samples, each labeled with Cy3 or Cy5, were co-hybridized to the *M. oryzae* 60-mer oligonucleotide microarray (G2519F-015060, Agilent Technologies) and were washed and dried as per the manufacturer’s protocols. The hybridized images were immediately scanned with an Agilent DNA microarray scanner, and data were extracted, background subtracted and normalized using the standard procedures of the Agilent Feature Extraction Software. All data normalization and selection of differentially expressed genes were performed using GeneSpringGX 7.3 (Agilent Technology, USA). Intensity-dependent normalization (LOWESS) was performed, where the ratio was reduced to the residual of the Lowess fit of the intensity versus ratio curve. The averages of the normalized ratios were calculated by dividing the average of the normalized signal channel intensity by the average of normalized control channel intensity. Genes that were differentially expressed were identified by filtering the mean ratios of fold-changes from replicates using *P*-value thresholds (where *P*<0.05 was considered to be significant) based on a *t-*test analysis.

### Quantitative Real Time PCR (qRT-PCR)

First-strand cDNA was synthesized from 2 µg RNA with an oligo (dT) primer using an Improm II RT-PCR kit (Promega, Madison, WI). The qRT-PCR reactions were performed following previously established procedures [31], [32]. Each qRT-PCR mixture (final volume 10 µl), which contained 5 µl Power SYBER Green PCR Master Mix (Applied Biosystems, Foster City, CA, USA), 3 µl forward and reverse primers (5 µM concentrations for each), and 2 µl of cDNA template (12.5 ng/ml), was subjected to PCR in the AB7500 Real-Time PCR system (Applied Biosystems). Reactions were performed using the following conditions: 1 cycle of 95°C for 3 min, and 40 cycles of 95°C for 15 s, 60°C for 30 s, and 72°C for 30s. To compare the relative expression levels of a target gene, the average threshold cycle (Ct) was normalized to that of *β*-tubulin (MGG00604) for each of the treated samples calculated as 2^−ΔCt^, where −ΔC_t_ = (C_t_, _target gene_−C_t_, _β-tubulin_). Fold changes during fungal development were calculated as 2^−ΔΔCt^, where −ΔΔC_t_  =  (C_t_, _target gene_−C_t_, _β-tubulin_) _test condition_ −(C_t_, _target gene_−C_t_, _β-tubulin_) _control_ [33]. The qRT-PCR was performed with three independent pools of tissues in two sets of experimental replicates. The primer pairs listed in Table S1 were independently designed for qRT-PCR to assess the relative expression levels of selected genes.

## Supporting Information

Table S1 Primer sequences used for qRT-PCR experiments.
(DOCX)

Table S2 Primer sequences used for generation of transformants expressing GFP behind *MoHOX2* promoter.
(DOCX)

Table S3 Genes induced during conidiation of *M. oryzae.*
(DOCX)

Table S4 Genes repressed during conidiation of *M. oryzae.*
(DOCX)

Table S5 Genes repressed during conidiation in the Δ*Mohox2* mutant.
(DOCX)

Table S6 Genes induced during conidiation in the Δ*Mohox2* mutant.
(DOCX)

Table S7 Genes induced in the wild type and repressed in the *MoHOX2* deletion mutant during conidiation.
(DOCX)
