# Supplementary material for: Gene Expression Profiling during Conidiation in the Rice Blast Pathogen Magnaporthe oryzae
Source: PLoS One. 2012 Aug 21;7(8):e43202. doi: 10.1371/journal.pone.0043202 (PMC3424150; doi:10.1371/journal.pone.0043202)
Supplement: Table S1 — Primer sequences used for qRT-PCR experiments. (DOCX) [file pone.0043202.s001.docx]

Table S1. Primer sequences used for qRT-PCR experiments

| Name | Sequence (5’ to 3’) |
| --- | --- |
| MGG10859F  MGG10859R  MGG10800F  MGG10800R  MGG12423F  MGG12423R  MGG14558F  MGG14558R  MGG09865F  MGG09865R  MGG00659F  MGG00659R  MGG01094F  MGG01094R  MGG08458F  MGG08458R  MGG13871F  MGG13871R  MGG14790F  MGG14790R  MGG14716F  MGG14716R  MGG12988F  MGG12988R  MGG10277F  MGG10277R  MGG10214F  MGG10214R  MGG10197F  MGG10197R  MGG10107F  MGG10107R  MGG07997F  MGG07997R  MGG07623F  MGG07623R  MGG07565F  MGG07565R  MGG06832F  MGG06832R  MGG06350F  MGG06350R  MGG05908F  MGG05908R  MGG05805F  MGG05805R  MGG05670F  MGG05670R  MGG05584F  MGG05584R  MGG04959F  MGG04959R  MGG 04346F  MGG 04346R  MGG03369F  MGG03369R  MGG02962F  MGG02962R  MGG02840F  MGG02840R  MGG02339F  MGG02339R  MGG03394F  MGG03394R  MGG05287F  MGG05287R  MGG01215F  MGG01215R  MGG09847F  MGG09847R  MGG02246F  MGG02246R  MGG00513F  MGG00513R  MGG02538F  MGG02538R  MGG14517F  MGG14517R  MGG00617F  MGG00617R  MGG14719F  MGG14719R  MGG01620F  MGG01620R  MGG08556F  MGG08556R  MGG12958F  MGG12958R  MGG04699F  MGG04699R  MGG06898F  MGG06898R  MGG01731F  MGG01731R  MGG07368F  MGG07368R | GGCCAACGTCAAGCTCATCAACAA  TCATGGTCTTGCCGTAGATGGACA  AGCTCCGACTTTATCATCTCGCCT  GTTGAGCATCTGCATCACGCACTT  TGAACGTCTCACCGCACTTCTTCA  AGTTCTTGTTGCGGTACACCTCGT  AATACCTGCGCAACACTCCGACTA  TGTCTGGGTACGCTTATGTGCTGT  CATTGCAACCACGAGGGACTCAAA  GAGCTTGCCCAGACAAAGCTGAAT  AATGCTCTGCAGAACGCCATCAAC  TGATGGTGCCAGTCACTCGGTAAA  CGGTGGGATTTGAGCAACGACAAA  AACACTGCAAAGTGGTGCGTCTTC  TGCAGGTCAACAACATTACGCACG  TCTGCAGATCGGCATACTCGTCAA  AGTTTGGCAACAACAGCAGCCA  GATGCCGGTCGTGAAGAAGAACAT  TTGCTGACCAGCTACCGGATGATT  ACGTCCACCGGCTGTTCTTCTAAA  ATGTCAACTGTAGAGGCAGCTGGT  AGAATTGAAGAGGAGAGCCATGCG  TCATCGGCATTTACTTTGCGCCAG  ATCATAGCGAGTGTCTCATCCGCA  GCGAGAACAAGGCCAAGATCTACA  AGCTCAAAGAGCACGACCATGAGA  AGGCAACCAAACTTCAAGCCAAGG  TGATGTGCTTTGCGTAGTTCAGGC  ATCGCGATGGTTTGGAGTGCAATG  TAAGCACGATGAGAATGGCCGAGT  TGATTTGGCCGCAAGACACCTTTG  TGCCGTGGATGATGAAGACGGAAA  TTGTAACAACAACGATCGTGCGCC  ATGTGCTTGAACTTGTCCATCGCC  AGCATCCGAGATATCTACGCCAGT  CAGGTCGTGCCGTTCTTGAAGATT  GATTGTGTGCGCGCTACGAATCAA  TGTTGGACCATAACGCAGTCCAGA  AGCGACGACGAAGAACTGTCAAGT  ACTGAGCTCAAGGGCTTCCTCTTT  ATCAGCACTCGCCTCAAACCTACA  AGTATTGCTGCTTGGCCTGTAAGC  ACCTACCTACGAGGACATTCGCAA  AGCGTCGTGTCTATGGATGCTTCT  ACGCAGTGCATGAAGCACGACT  AGTTGGCGCACAGGATGTTGATAC  TGACGAGGACGATGAAGAACACGA  GAGGACGTCCCATTGAGCAAATGA  GGTTGTCGAAATTCCATGTGCGGT  AAGCCCATGTCGTTATTCGGGTTG  CGGCTACACGCACTATGAGCTAAA  AAGATCGTCGGGAACCTTGTCTGT  AAGAAACCTTCGACGCCGTGTACT  TCATGACCCATTCGTACAGCCCAA  TGTGGTTCAGGATTACACTGGCGA  AGGAGCAGTTTGTCTGGTCGTTCT  AGCGCTCTCATTGACCACAAGTCT  AGTCGTCCGAGCATTGAGTTGTCA  TGGATACGCCGGTCAGAACATCAA  GGCGTTGGCAGTCAAGAAACTGAA  TTGCTTGCTCCTGCTTCGATCAGT  AACATCCGAGCACACCATCTTTGC  CTCAGCCCACATACAACTACCAGC  GACGACGATGATGATGGCGATG  CCAGGCTGGTTCGGATGTATCTC  CCTGGTTGGACCCTCCGT  AGCAGCAACGTACAAACTTC  GAAAGCTCATAGTGCAGTCG  CGACATGCTCAGCTCTTACAGG  TAGTCGTATCGCTCCGTGCCAT  CACAAGGCCAACCTCAA  TCTCCATCTCCTCGAGAC  GATTCATCCGCAGCAAA  CACATCTTGCCAAACAGG  AGCTCGAGATTCCTTGCCCA  CAATCCGTTACAGCCTCATCCC  AGCGCTGGATGGAGTCAAAGAG  TACGGCGTTTTGTGCATCCTC  GTACATGCACGGACAACAAG  CTGATCAGCTGGACAATGG  CGTGCACTGCTCCATCTTAC  CTCGTTTCGCCTCAAGTTC  GCACCCTCCTCTACTACTGAAG  GTGACGGACAAGATTTACCTC  GCCGGTTACTTCCTATTCC  CCGACTTGATCTCCATTCTG  CAACAATACCCCATGCCCTCTGA  CGTGCCCTGCGGACAAC  AGCCGACGGTTTATCTTTCTCTGG  TTCTTGCAAGGCTCGTAGGGGTAG  CGTAGGGGCGGCTTCTCTT  TCAACCCGACCCCGATACTCC  GCACACCAAAGGCCCATCTGA  GCTGCCGGTTCCCTTTGTAGTAG  GGTCAACCCTCAGCTCCAACAAG  CTCGGCGCGGTCCAGAAT |
