# Supplementary material for: Gene Expression Profiling during Conidiation in the Rice Blast Pathogen Magnaporthe oryzae
Source: PLoS One. 2012 Aug 21;7(8):e43202. doi: 10.1371/journal.pone.0043202 (PMC3424150; doi:10.1371/journal.pone.0043202)
Supplement: Table S2 — Primer sequences used for generation of transformants expressing GFP behind MoHOX2 promoter. (DOCX) [file pone.0043202.s002.docx]

**Table S2.** Primer sequences used for generation of transformants expressing GFP behind *MoHOX2* promoter

| Name | Sequence (5’ to 3’) |
| --- | --- |
| H2PF1 | TGGAGATAAGCTCACAACGAGAGAGG |
| H2PF2 | CCAAGATGCTAGCTAGGGTTGCAC |
| H2PR | AGCTCCTCGCCCTTGCTCACCATGTAGTCCATGCTTTTTTTGCTGTCGC |
| GHF | ACAGCAAAAAAAGCATGGACTACATGGTGAGCAAGGGCGAGGAGCTGTT |
| GHR | GGGTTCCGCGCACATTTCC |
