# Supplementary material for: Gene Expression Profiling during Conidiation in the Rice Blast Pathogen Magnaporthe oryzae
Source: PLoS One. 2012 Aug 21;7(8):e43202. doi: 10.1371/journal.pone.0043202 (PMC3424150; doi:10.1371/journal.pone.0043202)
Supplement: Table S3 — Genes induced during conidiation of M. oryzae. (DOCX) [file pone.0043202.s003.docx]

**Table S3.** Genes induced during conidiation of *M. oryzae*

| **Locus** | **Fold-induction during conidiation in the wild-type^a^** | **Annotation** | **InterPro domain search** |
| --- | --- | --- | --- |
| MGG 02371.6 | 35.34 | alcohol dehydrogenase | IPR000172: Glucose-methanol-choline oxidoreductase, N-terminal |
| MGG 02840.6 | 35.33 | conserved hypothetical protein | IPR011701: Major facilitator superfamily MFS-1 |
| MGG 09847.6 | 32.42 | MoACR1 | No defined Interpro term |
| MGG 01094.6 | 31.92 | conserved hypothetical protein | IPR000008: C2 calcium-dependent membrane targeting region, CaLB |
| MGG10197.6 | 28.60 | conserved hypothetical protein | IPR000054: Ribosomal protein L31e |
| MGG07565.6 | 27.65 | conserved hypothetical protein | IPR008427: Extracellular membrane protein, 8-cysteine region, CFEM |
| MGG02339.6 | 27.29 | hypothetical protein | IPR011058: Cyanovirin-N |
| MGG10257.6 | 25.89 | conserved hypothetical protein | No defined Interpro term |
| MGG07997.6 | 25.42 | covalently-linked cell wall protein | IPR000420: Yeast PIR protein repeat |
| MGG05109.6 | 23.35 | conserved hypothetical protein | No defined Interpro term |
| MGG09055.6 | 23.21 | hypothetical protein | No defined Interpro term |
| MGG15378.6 | 23.16 | hypothetical protein | No defined Interpro term |
| MGG03085.6 | 23.05 | conserved hypothetical protein | IPR001283: Allergen V5/Tpx-1 related, IPR014044 : SCP-like extracellular |
| MGG03409.6 | 22.98 | conserved hypothetical protein | IPR011701: Major facilitator superfamily MFS-1 |
| MGG06446.6 | 22.74 | 5-aminolevulinate synthase, mitochondrial precursor | IPR001917: Aminotransferase, class-II, IPR015424: Pyridoxal phosphate-dependent transferase |
| MGG05366.6 | 22.64 | feruloyl esterase B | IPR011118: Tannase and feruloyl esterase |
| MGG14558.6 | 21.70 | conserved hypothetical protein | IPR001005: SANT, DNA-binding, IPR009057 : Homeodomain-like |
| MGG10107.6 | 21.09 | caleosin domain-containing protein | IPR007736: Caleosin related |
| MGG10571.6 | 21.01 | conserved hypothetical protein | No defined Interpro term |
| MGG04346.6 | 20.26 | sterol 24-C-methyltransferase | IPR013216: Methyltransferase type 11, IPR013705: Sterol methyltransferase C-terminal |
| MGG00659.6 | 19.67 | glucan 1,3-beta-glucosidase | IPR011050: Pectin lyase fold/virulence factor |
| MGG05805.6 | 19.49 | hypothetical protein | No defined Interpro term |
| MGG10792.6 | 19.44 | conserved hypothetical protein | IPR006076: FAD dependent oxidoreductase |
| MGG03793.6 | 18.98 | 2,3-dihydroxybenzoic acid decarboxylase | IPR006992: Amidohydrolase 2 |
| MGG00334.6 | 18.74 | hypothetical protein | No defined Interpro term |
| MGG09604.6 | 18.36 | conserved hypothetical protein | IPR008427: Extracellular membrane protein, 8-cysteine region, CFEM |
| MGG08412.6 | 18.26 | conserved hypothetical protein | No defined Interpro term |
| MGG07623.6 | 17.67 | hypothetical protein | IPR001002: Chitin-binding, type 1, |
| MGG12421.6 | 17.55 | aminomethyltransferase | IPR006076: FAD dependent oxidoreductase, IPR006222: Glycine cleavage T-protein |
| MGG01046.6 | 17.42 | methionyl-tRNA synthetase | IPR001412: Aminoacyl-tRNA synthetase, class I, conserved site, IPR002304 : Methionyl-tRNA synthetase, class Ia |
| MGG02884.6 | 17.40 | beta-Ig-H3/Fasciclin | IPR000782: FAS1 domain,IPR000782 : FAS1 domain |
| MGG04959.6 | 17.29 | hypothetical protein | No defined Interpro term |
| MGG10859.6 | 16.89 | linoleate diol synthase | IPR001128: Cytochrome P450, IPR010255 : Haem peroxidase |
| MGG03466.6 | 16.83 | conserved hypothetical protein | IPR010895: CHRD |
| MGG06832.6 | 16.48 | conserved hypothetical protein | IPR001138: Fungal transcriptional regulatory protein |
| MGG04378.6 | 16.25 | integral membrane protein | No defined Interpro term |
| MGG06567.6 | 16.06 | conserved hypothetical protein | No defined Interpro term |
| MGG09116.6 | 16.04 | xylulose-5-phosphate/fructose-6-phosphate phosphoketolase | IPR005593: D-xylulose 5-phosphate/D-fructose 6-phosphate phosphoketolase, IPR009014: Transketolase, C-terminal/Pyruvate-ferredoxin oxidoreductase, domain II |
| MGG05327.6 | 15.01 | hypothetical protein | No defined Interpro term |
| MGG03403.6 | 14.37 | hypothetical protein | No defined Interpro term |
| MGG05573.6 | 14.26 | conserved hypothetical protein | No defined Interpro term |
| MGG09985.6 | 14.08 | hypothetical protein | No defined Interpro term |
| MGG13013.6 | 14.07 | chitin synthase 8 | IPR001199: Cytochrome b5, IPR001609 : Myosin head, IPR004835: Fungal chitin synthase |
| MGG05574.6 | 13.99 | conserved hypothetical protein | IPR006076: FAD dependent oxidoreductase |
| MGG09865.6 | 13.82 | integral membrane protein | No defined Interpro term |
| MGG03369.6 | 13.72 | conserved hypothetical protein | IPR012674: Calycin |
| MGG05584.6 | 13.71 | conserved hypothetical protein | IPR004854: Ubiquitin fusion degradation protein UFD1 |
| MGG05908.6 | 13.71 | cytochrome P450 52A11 | IPR002974: Cytochrome P450, E-class, CYP52 |
| MGG00244.6 | 13.64 | 15-hydroxyprostaglandin dehydrogenase | IPR002198: Short-chain dehydrogenase/reductase SDR, IPR002347: Glucose/ribitol dehydrogenase, IPR016040: NAD(P)-binding,IPR016040 : NAD(P)-binding |
| MGG02837.6 | 13.62 | conserved hypothetical protein | No defined Interpro term |
| MGG07924.6 | 13.50 | hypothetical protein | No defined Interpro term |
| MGG00290.6 | 13.44 | hypothetical protein | No defined Interpro term |
| MGG10214.6 | 13.08 | fumarylacetoacetate hydrolase domain-containing protein 2 | IPR002529: Fumarylacetoacetase, C-terminal-like |
| MGG00359.6 | 13.08 | Delta(3,5)-Delta(2,4)-dienoyl-CoA isomerase | IPR001753: Crotonase, core |
| MGG01932.6 | 13.07 | hypothetical protein | No defined Interpro term |
| MGG12988.6 | 12.98 | alpha-glucoside transport protein | IPR003663: Sugar/inositol transporter, IPR005828: General substrate transporter, IPR016196 : Major facilitator superfamily, general substrate transporter |
| MGG09102.6 | 12.59 | laccase-1 | IPR001117: Multicopper oxidase, type 1, IPR002355: Multicopper oxidase, copper-binding site |
| MGG07311.6 | 12.48 | hypothetical protein | No defined Interpro term |
| MGG00748.6 | 12.37 | myosin-5 | IPR000048: IQ calmodulin-binding region, IPR001452: Src homology-3 domain, IPR001609: Myosin head, motor region, IPR010926: Myosin tail 2 |
| MGG05398.6 | 12.23 | hypothetical protein |  |
| MGG06315.6 | 12.18 | transcription regulatory protein SNF5 | IPR006939: SNF5/SMARCB1/INI1 |
| MGG12750.6 | 11.96 | conserved hypothetical protein | IPR000637: HMG-I and HMG-Y, DNA-binding, conserved site |
| MGG04575.6 | 11.89 | conserved hypothetical protein | No defined Interpro term |
| MGG01856.6 | 11.70 | conserved hypothetical protein | IPR006598: Lipopolysaccharide-modifying protein |
| MGG00635.6 | 11.51 | conserved hypothetical protein | No defined Interpro term |
| MGG10800.6 | 11.46 | sarcosine oxidase | IPR006076: FAD dependent oxidoreductase |
| MGG08846.6 | 11.45 | conserved hypothetical protein | IPR000759: Adrenodoxin reductase, IPR013027: FAD-dependent pyridine nucleotide-disulphide oxidoreductase, |
| MGG06489.6 | 11.31 | NADP-dependent alcohol dehydrogenase 6 | IPR002085: Alcohol dehydrogenase superfamily, zinc-containing, IPR016040: NAD(P)-binding |
| MGG04911.6 | 11.28 | cytochrome P450 3A5 | IPR001128: Cytochrome P450, IPR002401: Cytochrome P450, E-class, group I |
| MGG08458.6 | 11.28 | chitinase 1 precursor | IPR001223: Glycoside hydrolase, family 18, catalytic domain, IPR001579: Glycoside hydrolase, chitinase active site, IPR011583: Chitinase II, |
| MGG03482.6 | 11.16 | hypothetical protein | No defined Interpro term |
| MGG02611.6 | 11.15 | L-aminoadipate-semialdehyde dehydrogenase large subunit | IPR00087 : AMP-dependent synthetase and ligase, IPR006163: Phosphopantetheine-binding, IPR010071: Amino acid adenylation, IPR010080: Thioester reductase, IPR013120: Male sterility, NAD-binding, IPR014397: L-aminoadipate-semialdehyde dehydrogenase, large subunit, IPR016040: NAD(P)-binding, |
| MGG02612.6 | 11.14 | 3-oxoacyl-[acyl-carrier-protein] reductase | IPR002198: Short-chain dehydrogenase/reductase SDR, IPR016040: NAD(P)-binding |
| MGG08926.6 | 11.14 | conserved hypothetical protein | No defined Interpro term |
| MGG03558.6 | 11.12 | PH domain-containing protein | No defined Interpro term |
| MGG05035.6 | 10.97 | hypothetical protein | No defined Interpro term |
| MGG07639.6 | 10.92 | excitatory amino acid transporter 2 | IPR001991: Sodium:dicarboxylate symporter |
| MGG06877.6 | 10.84 | DUF618 domain-containing protein | IPR006569: Regulation of nuclear pre-mRNA protein, IPR006903: Protein of unknown function DUF618, IPR008942: ENTH/VHS |
| MGG08758.6 | 10.81 | aminopeptidase Y | IPR003137: Protease-associated PA, IPR007484: Peptidase M28 |
| MGG12522.6 | 10.56 | hypothetical protein | No defined Interpro term |
| MGG06298.6 | 10.50 | hypothetical protein | No defined Interpro term |
| MGG04774.6 | 10.39 | conserved hypothetical protein | IPR005645: Protein of unknown function DUF341 |
| MGG05733.6 | 10.34 | hypothetical protein | No defined Interpro term |
| MGG15028.6 | 10.19 | hypothetical protein | No defined Interpro term |
| MGG11271.6 | 10.13 | hypothetical protein | No defined Interpro term |
| MGG12423.6 | 10.10 | choline monooxygenase |  |
| MGG04925.6 | 9.97 | hypothetical protein | No defined Interpro term |
| MGG01910.6 | 9.87 | hypothetical protein | No defined Interpro term |
| MGG07289.6 | 9.86 | glycogen synthase | IPR008631: Glycogen synthase |
| MGG09575.6 | 9.82 | conserved hypothetical protein | IPR001128: Cytochrome P450, IPR011041: Soluble quinoprotein glucose dehydrogenase, IPR011042: Six-bladed beta-propeller, TolB-like |
| MGG03336.6 | 9.80 | LEA domain-containing protein | No defined Interpro term |
| MGG00225.6 | 9.79 | hypothetical protein | No defined Interpro term |
| MGG09107.6 | 9.78 | conserved hypothetical protein | IPR001202: WW/Rsp5/WWP, IPR010730: Heterokaryon incompatibility |
| MGG04525.6 | 9.77 | conserved hypothetical protein | No defined Interpro term |
| MGG14693.6 | 9.74 | conserved hypothetical protein | No defined Interpro term |
| MGG01458.6 | 9.71 | hypothetical protein | No defined Interpro term |
| MGG14790.6 | 9.64 | hypothetical protein | IPR002114: Phosphotransferase system, HPr serine phosphorylation site |
| MGG08794.6 | 9.64 | conserved hypothetical protein | IPR002110: Ankyrin |
| MGG09569.6 | 9.63 | hypothetical protein | No defined Interpro term |
| MGG07150.6 | 9.62 | conserved hypothetical protein | No defined Interpro term |
| MGG13405.6 | 9.55 | geranylgeranyl pyrophosphate synthetase | IPR000092: Polyprenyl synthetase, IPR008949: Terpenoid synthase, IPR017446: Polyprenyl synthetase-related |
| MGG07088.6 | 9.54 | hypothetical protein | No defined Interpro term |
| MGG08122.6 | 9.51 | DNA replication licensing factor mcm2 | IPR001208: DNA-dependent ATPase MCM, IPR008045: MCM protein 2, IPR016027: Nucleic acid-binding, OB-fold-like |
| MGG02403.6 | 9.38 | hypothetical protein | No defined Interpro term |
| MGG08332.6 | 9.20 | conserved hypothetical protein | IPR000194: ATPase, F1/V1/A1 complex, alpha/beta subunit, nucleotide-binding, IPR000568: ATPase, F0 complex, subunit A, IPR008010: Membrane protein,Tapt1/CMV receptor, |
| MGG01366.6 | 9.12 | hypothetical protein | No defined Interpro term |
| MGG11761.6 | 9.06 | conserved hypothetical protein | No defined Interpro term |
| MGG04594.6 | 9.04 | conserved hypothetical protein | IPR002523: Mg2+ transporter protein, CorA-like |
| MGG08486.6 | 8.99 | beta-lactamase family protein | IPR000871: Beta-lactamase |
| MGG05433.6 | 8.92 | conserved hypothetical protein | IPR000175: Sodium:neurotransmitter symporter |
| MGG10277.6 | 8.86 | brefeldin A resistance protein | IPR003593: ATPase, AAA+ type, core, IPR010929: CDR ABC transporter, IPR013525: ABC-2 type transporter, IPR017871: ABC transporter, conserved site, |
| MGG10710.6 | 8.71 | oxidoreductase | IPR006076: FAD dependent oxidoreductase, |
| MGG10031.6 | 8.66 | hypothetical protein | No defined Interpro term |
| MGG05670.6 | 8.46 | conserved hypothetical protein | No defined Interpro term |
| MGG14184.6 | 8.53 | conserved hypothetical protein | No defined Interpro term |
| MGG07166.6 | 8.53 | conserved hypothetical protein | IPR000408: Regulator of chromosome condensation, RCC1, IPR009091: Regulator of chromosome condensation/beta-lactamase-inhibitor protein II |
| MGG12770.6 | 8.52 | conserved hypothetical protein | No defined Interpro term |
| MGG08459.6 | 8.50 | ent-kaurene oxidase | IPR001128 : Cytochrome P450 |
| MGG03279.6 | 8.37 | hypothetical protein | No defined Interpro term |
| MGG02775.6 | 8.28 | conserved hypothetical protein | IPR007087: Zinc finger, C2H2-type |
| MGG11816.6 | 8.24 | NADPH-dependent 1-acyldihydroxyacetone phosphate reductase | IPR002198: Short-chain dehydrogenase/reductase SDR, IPR002347: Glucose/ribitol dehydrogenase, IPR016040: NAD(P)-binding |
| MGG09650.6 | 8.15 | conserved hypothetical protein | No defined Interpro term |
| MGG00346.6 | 7.98 | pi-transporter A-1 | IPR005828: General substrate transporter, IPR016196: Major facilitator superfamily, general substrate transporter |
| MGG04173.6 | 7.93 | conserved hypothetical protein | No defined Interpro term |
| MGG11663.6 | 7.86 | zinc-binding oxidoreductase CipB | IPR002085: Alcohol dehydrogenase superfamily, zinc-containing, IPR011032: GroES-like |
| MGG06144.6 | 7.86 | hypothetical protein | No defined Interpro term |
| MGG10245.6 | 7.80 | conserved hypothetical protein | No defined Interpro term |
| MGG04554.6 | 7.77 | conserved hypothetical protein | No defined Interpro term |
| MGG02979.6 | 7.77 | conserved hypothetical protein |  |
| MGG06538.6 | 7.75 | Bys1 family protein | No defined Interpro term |
| MGG12552.6 | 7.73 | hypothetical protein | No defined Interpro term |
| MGG07771.6 | 7.68 | laccase-2 | IPR001117: Multicopper oxidase, type 1, IPR008972: Cupredoxin, IPR011706: Multicopper oxidase |
| MGG10244.6 | 7.58 | hypothetical protein | No defined Interpro term |
| MGG01485.6 | 7.53 | conserved hypothetical protein | IPR011701: Major facilitator superfamily MFS-1, IPR016196: Major facilitator superfamily, general substrate transporter |
| MGG02918.6 | 7.37 | hypothetical protein | IPR001064 : Beta and gamma crystallin |
| MGG09922.6 | 7.36 | beta-hexosaminidase beta chain | IPR015882: Beta-N-acetylhexosaminidase-like, IPR015883: Glycoside hydrolase, family 20, catalytic core |
| MGG02043.6 | 7.35 | BTB/POZ domain-containing protein | No defined Interpro term |
| MGG07901.6 | 7.31 | conserved hypothetical protein | No defined Interpro term |
| MGG02879.6 | 7.14 | hypothetical protein | IPR001138: Fungal transcriptional regulatory protein |
| MGG08296.6 | 7.13 | hypothetical protein | No defined Interpro term |
| MGG06254.6 | 7.10 | chalcone synthase | IPR001099: Chalcone/stilbene synthase, N-terminal, IPR011141: Polyketide synthase, type III, IPR0123 : Chalcone and stilbene synthases, C-terminal |
| MGG12469.6 | 7.08 | hypothetical protein | No defined Interpro term |
| MGG05987.6 | 7.07 | hypothetical protein | IPR002125: CMP/dCMP deaminase, zinc-binding |
| MGG09802.6 | 7.02 | conserved hypothetical protein | No defined Interpro term |
| MGG14662.6 | 6.98 | hypothetical protein | No defined Interpro term |
| MGG04698.6 | 6.97 | PQ-loop repeat-containing protein 2 | IPR006603: Cystinosin/ERS1p repeat |
| MGG00846.6 | 6.96 | L-fucose permease Glucose/galactose transporter | IPR011701: Major facilitator superfamily MFS-1, IPR016196: Major facilitator superfamily, general substrate transporter |
| MGG02778.6 | 6.92 | hypothetical protein | No defined Interpro term |
| MGG00722.6 | 6.86 | hydantoinase |  |
| MGG04778.6 | 6.85 | conserved hypothetical protein | No defined Interpro term |
| MGG09863.6 | 6.79 | conserved hypothetical protein | No defined Interpro term |
| MGG05165.6 | 6.74 | conserved hypothetical protein | No defined Interpro term |
| MGG02069.6 | 6.73 | glyoxalase/bleomycin resistance protein/dioxygenase | IPR004360: Glyoxalase/bleomycin resistance protein/dioxygenase |
| MGG02641.6 | 6.70 | conserved hypothetical protein | No defined Interpro term |
| MGG04595.6 | 6.69 | hypothetical protein | No defined Interpro term |
| MGG07002.6 | 6.67 | hypothetical protein | No defined Interpro term |
| MGG08941.6 | 6.66 | hypothetical protein | IPR011058: Cyanovirin-N |
| MGG02109.6 | 6.63 | conserved hypothetical protein | No defined Interpro term |
| MGG05100.6 | 6.62 | hypothetical protein | IPR001283: Allergen V5/Tpx-1 related, IPR014044 : SCP-like extracellular |
| MGG07402.6 | 6.59 | conserved hypothetical protein | IPR015075: Protein of unknown function DUF1857 |
| MGG08917.6 | 6.59 | conserved hypothetical protein | IPR001138: Fungal transcriptional regulatory protein, N-terminal |
| MGG01257.6 | 6.58 | tRNA-dihydrouridine synthase 4 | IPR001269: tRNA-dihydrouridine synthase, IPR013785: Aldolase-type TIM barrel, |
| MGG05075.6 | 6.57 | hypothetical protein | No defined Interpro term |
| MGG09954.6 | 6.48 | conserved hypothetical protein | IPR000219: Dbl homology (DH) domain |
| MGG14995.6 | 6.44 | hypothetical protein | No defined Interpro term |
| MGG01436.6 | 6.38 | conserved hypothetical protein | IPR006034: Asparaginase/glutaminase |
| MGG06225.6 | 6.38 | hypothetical protein | No defined Interpro term |
| MGG08429.6 | 6.19 | serin endopeptidas | IPR000209: Peptidase S8 and S53, subtilisin, kexin, sedolisin |
| MGG03000.6 | 6.15 | conserved hypothetical protein | No defined Interpro term |
| MGG13667.6 | 6.15 | hypothetical protein | No defined Interpro term |
| MGG03414.6 | 6.15 | short chain dehydrogenase/reductase family | IPR002198: Short-chain dehydrogenase/reductase SDR, IPR002347: Glucose/ribitol dehydrogenase, IPR016040: NAD(P)-binding |
| MGG11537.6 | 6.14 | conserved hypothetical protein | IPR002573: Choline/ethanolamine kinase, IPR008266: Tyrosine protein kinase, active site |
| MGG07774.6 | 6.10 | hypothetical protein | No defined Interpro term |
| MGG14694.6 | 6.09 | hypothetical protein | No defined Interpro term |
| MGG05342.6 | 6.01 | ribonuclease Trv | IPR001568: Ribonuclease T2 |
| MGG00176.6 | 5.96 | hypothetical protein | No defined Interpro term |
| MGG07870.6 | 5.94 | conserved hypothetical protein | No defined Interpro term |
| MGG07710.6 | 5.92 | conserved hypothetical protein | No defined Interpro term |
| MGG00450.6 | 5.92 | phosphoenolpyruvate carboxykinase | IPR001272: Phosphoenolpyruvate carboxykinase, ATP-utilising |
| MGG00888.6 | 5.91 | hypothetical protein | No defined Interpro term |
| MGG02329.6 | 5.87 | isotrichodermin C-15 hydroxylase | IPR001128: Cytochrome P450, IPR001209: Ribosomal protein S14, IPR002401: Cytochrome P450, E-class, group I |
| MGG08703.6 | 5.86 | hypothetical protein | No defined Interpro term |
| MGG13179.6 | 5.80 | hypothetical protein | No defined Interpro term |
| MGG04557.6 | 5.78 | conserved hypothetical protein | IPR002935: O-methyltransferase, family 3 |
| MGG04956.6 | 5.63 | long-chain-fatty-acid-CoA ligase 1 | IPR000873: AMP-dependent synthetase and ligase |
| MGG04880.6 | 5.63 | conserved hypothetical protein | No defined Interpro term |
| MGG14555.6 | 5.60 | hypothetical protein | No defined Interpro term |
| MGG06396.6 | 5.56 | conserved hypothetical protein | IPR003890: MIF4G-like, type 3, IPR016021: MIF4-like, type 1/2/3, IPR016024: Armadillo-type fold |
| MGG03349.6 | 5.47 | auxin Efflux Carrier superfamily | IPR004776 : Auxin efflux carrier |
| MGG02817.6 | 5.42 | glutamate decarboxylase | IPR000760: Inositol monophosphatase, IPR002129: Pyridoxal phosphate-dependent decarboxylase, IPR010107: Glutamate decarboxylase,IPR015421 : Pyridoxal phosphate-dependent transferase, major region, subdomain 1 |
| MGG00655.6 | 5.40 | peroxisomal biogenesis factor 2 | IPR001841: Zinc finger, RING-type, IPR006845: Pex, N-terminal, IPR013083: Zinc finger, RING/FYVE/PHD-type |
| MGG06239.6 | 5.40 | conserved hypothetical protein | IPR016624: Uncharacterised conserved protein UCP014753, |
| MGG09930.6 | 5.36 | chromodomain-helicase-DNA-binding protein 3 | IPR000330: SNF2-related, IPR001650: DNA/RNA helicase, C-terminal, IPR014021: Helicase, superfamily 1 and 2, ATP-binding |
| MGG05295.6 | 5.35 | conserved hypothetical protein | There No defined Interpro term |
| MGG07789.6 | 5.34 | 3-phytase | IPR000742: EGF-like, type 3, IPR003431: Phytase |
| MGG07198.6 | 5.33 | conserved hypothetical protein | IPR001680: WD40 repeat |
| MGG06572.6 | 5.30 | phosphatidylinositol-4-phosphate 5-kinase its3 (PtdIns(4)P-5-kinase) | IPR002498: Phosphatidylinositol-4-phosphate 5-kinase, core |
| MGG11285.6 | 5.29 | hypothetical protein | No defined Interpro term |
| MGG01933.6 | 5.29 | conserved hypothetical protein | No defined Interpro term |
| MGG05857.6 | 5.25 | histone deacetylase RPD3 | IPR000286: Histone deacetylase superfamily |
| MGG08487.6 | 5.25 | cellobiose dehydrogenase | IPR000172: Glucose-methanol-choline oxidoreductase |
| MGG09138.6 | 5.25 | glutathione S-transferase II | IPR004045: Glutathione S-transferase, N-terminal, IPR004046: Glutathione S-transferase, C-terminal, IPR012335: Thioredoxin fold |
| MGG00585.6 | 5.24 | hypothetical protein | No defined Interpro term |
| MGG15343.6 | 5.22 | hypothetical protein | No defined Interpro term |
| MGG08110.6 | 5.22 | hypothetical protein | No defined Interpro term |
| MGG00841.6 | 5.21 | exonuclease 1 |  |
| MGG06347.6 | 5.19 | conserved hypothetical protein | No defined Interpro term |
| MGG04469.6 | 5.16 | cytochrome P450 97B3 | IPR001128: Cytochrome P450 |
| MGG04487.6 | 5.13 | conserved hypothetical protein | No defined Interpro term |
| MGG02294.6 | 5.11 | ent-kaurene oxidase | IPR001128: Cytochrome P450 |
| MGG01261.6 | 5.11 | trehalase | IPR00166: Glycoside hydrolase, family 37, IPR008928: Six-hairpin glycosidase-like |
| MGG00369.6 | 5.09 | cell division cycle protein 123 | IPR009772: D123 |
| MGG14716.6 | 5.04 | hypothetical protein | No defined Interpro term |
| MGG01367.6 | 5.02 | conserved hypothetical protein | IPR015141: Phospholipase A2, prokaryotic/fungal, IPR016090: Phospholipase A2 |
| MGG04166.6 | 5.01 | salicylate hydroxylase | IPR003042: Aromatic-ring hydroxylase-like, IPR013027: FAD-dependent pyridine nucleotide-disulphide oxidoreductase |
| MGG09533.6 | 4.99 | hypothetical protein | No defined Interpro term |
| MGG04353.6 | 4.98 | hypothetical protein | No defined Interpro term |
| MGG03287.6 | 4.96 | alpha-amylase | IPR006047: Glycosyl hydrolase, family 13, catalytic region, IPR013776: Alpha-amylase, thermostable |
| MGG07157.6 | 4.96 | MFS hexose transporter | IPR003663: Sugar/inositol transporter, IPR005828: General substrate transporter, IPR005829: Sugar transporter, conserved site |
| MGG06323.6 | 4.94 | solute carrier family 35 member E3 | IPR004853: Protein of unknown function DUF250 |
| MGG12598.6 | 4.89 | pantothenate kinase | IPR004567: Eukaryotic pantothenate kinase, IPR011602: Fumble |
| MGG00607.6 | 4.88 | conserved hypothetical protein | IPR002575: Aminoglycoside phosphotransferase, IPR011009: Protein kinase-like |
| MGG01391.6 | 4.87 | ent-kaurene oxidase | IPR001128: Cytochrome P450, IPR002403: Cytochrome P450, E-class, group IV |
| MGG05938.6 | 4.85 | 2,2-dialkylglycine decarboxylase | IPR005814: Aminotransferase class-III, IPR015421: Pyridoxal phosphate-dependent transferase, major region, subdomain 1 |
| MGG02070.6 | 4.84 | hypothetical protein | No defined Interpro term |
| MGG03689.6 | 4.83 | 3-oxoacyl-[acyl-carrier-protein] reductase | IPR002198: Short-chain dehydrogenase/reductase SDR, IPR016040: NAD(P)-binding |
| MGG12176.6 | 4.80 | conserved hypothetical protein | No defined Interpro term |
| MGG09362.6 | 4.77 | hypothetical protein | No defined Interpro term |
| MGG09576.6 | 4.75 | hypothetical protein | No defined Interpro term |
| MGG06926.6 | 4.74 | cyclopropane-fatty-acyl-phospholipid synthase | IPR013216: Methyltransferase type 11, |
| MGG08810.6 | 4.73 | 2,5-diketo-D-gluconic acid reductase A | IPR001395: Aldo/keto reductase |
| MGG03826.6 | 4.72 | kelch repeat protein | IPR006652: Kelch repeat type 1, IPR011043: Galactose oxidase/kelch, beta-propeller |
| MGG03889.6 | 4.69 | threonine synthase | IPR001926: Pyridoxal phosphate-dependent enzyme, beta subunit |
| MGG05357.6 | 4.68 | conserved hypothetical protein | IPR006683: Thioesterase superfamily |
| MGG08768.6 | 4.68 | methyltransferase-UbiE family protein | IPR013216: Methyltransferase type 11 |
| MGG01883.6 | 4.67 | conserved hypothetical protein | IPR011701: Major facilitator superfamily MFS-1, IPR016196 : Major facilitator superfamily, general substrate transporter |
| MGG08161.6 | 4.67 | conserved hypothetical protein | IPR013217: Methyltransferase type 12 |
| MGG07033.6 | 4.66 | ATP-dependent RNA helicase ded-1 |  |
| MGG03041.6 | 4.66 | glucokinase | IPR001312: Hexokinase |
| MGG00981.6 | 4.65 | aspartic endopeptidase | IPR001461: Peptidase A1, IPR009007 : Peptidase aspartic, catalytic |
| MGG02849.6 | 4.62 | candidapepsin-8 | IPR001461: Peptidase A1, IPR001969: Peptidase aspartic, active site |
| MGG05202.6 | 4.56 | hypothetical protein | No defined Interpro term |
| MGG02962.6 | 4.56 | C6 zinc finger domain-containing protein | IPR001138: Fungal transcriptional regulatory protein, N-terminal |
| MGG03896.6 | 4.53 | conserved hypothetical protein | No defined Interpro term |
| MGG09840.6 | 4.50 | acetylxylan esterase 2 | IPR000675: Cutinase |
| MGG02955.6 | 4.48 | IMP-specific 5'-nucleotidase 1 | IPR009453: IMP-specific 5-nucleotidase |
| MGG14006.6 | 4.47 | hypothetical protein | No defined Interpro term |
| MGG09726.6 | 4.44 | arabinogalactan endo-1,4-beta-galactosidase | IPR011683: Glycosyl hydrolase 53, IPR013781: Glycoside hydrolase, subgroup, catalytic core |
| MGG01988.6 | 4.41 | conserved hypothetical protein | IPR003121: SWIB/MDM2, IPR014876: DEK, C-terminal |
| MGG07872.6 | 4.39 | conserved hypothetical protein | No defined Interpro term |
| MGG09263.6 | 4.36 | C6 zinc finger domain-containing protein | IPR001138: Fungal transcriptional regulatory protein, N-terminal, IPR007219 : Fungal specific transcription factor |
| MGG02557.6 | 4.35 | hypothetical protein | No defined Interpro term |
| MGG15447.6 | 4.32 | conserved hypothetical protein | No defined Interpro term |
| MGG13871.6 | 4.32 | hypothetical protein | IPR006629: LPS-induced tumor necrosis factor alpha factor |
| MGG01752.6 | 4.31 | conserved hypothetical protein | IPR011011: Zinc finger, FYVE/PHD-type |
| MGG01102.6 | 4.31 | ornithine carbamoyltransferase | IPR002292: Ornithine carbamoyltransferase, IPR006131: Aspartate/ornithine carbamoyltransferase, Asp/Orn-binding region |
| MGG06519.6 | 4.25 | WD domain-containing protein | IPR001680: WD40 repeat |
| MGG10597.6 | 4.25 | RTA1 domain-containing protein | No defined Interpro term |
| MGG01552.6 | 4.23 | conserved hypothetical protein | No defined Interpro term |
| MGG00636.6 | 4.22 | GPI mannosyltransferase 2 | IPR007315: Mannosyltransferase, PIG-V |
| MGG08994.6 | 4.21 | conserved hypothetical protein | IPR000868: Isochorismatase hydrolase, |
| MGG14821.6 | 4.20 | hypothetical protein | No defined Interpro term |
| MGG06605.6 | 4.19 | DUF895 domain membrane protein | IPR010291: Protein of unknown function DUF895, eukaryotic, IPR016196: Major facilitator superfamily, general substrate transporter, |
| MGG09988.6 | 4.18 | glycosyl hydrolase family 43 protein | IPR006710: Glycoside hydrolase, family 43 |
| MGG12383.6 | 4.17 | conserved hypothetical protein | No defined Interpro term |
| MGG04345.6 | 4.16 | cytochrome P450 17A1 | IPR001128: Cytochrome P450 |
| MGG04556.6 | 4.14 | alcohol dehydrogenase 1 | IPR002085: Alcohol dehydrogenase superfamily, zinc-containing, IPR016040: NAD(P)-binding |
| MGG06534.6 | 4.13 | retinol dehydrogenase 12 | IPR002198: Short-chain dehydrogenase/reductase SDR, IPR002347: Glucose/ribitol dehydrogenase, IPR016040 : NAD(P)-binding |
| MGG01143.6 | 4.13 | hypothetical protein | No defined Interpro term |
| MGG01867.6 | 4.11 | conserved hypothetical protein | No defined Interpro term |
| MGG05717.6 | 4.10 | conserved hypothetical protein | No defined Interpro term |
| MGG03597.6 | 4.08 | conserved hypothetical protein | No defined Interpro term |
| MGG02110.6 | 4.07 | caleosin domain-containing protein | IPR007736: Caleosin related |
| MGG05356.6 | 4.07 | conserved hypothetical protein | No defined Interpro term |
| MGG08193.6 | 4.05 | hypothetical protein | IPR013838: Beta tubulin, autoregulation binding site |
| MGG09474.6 | 4.05 | hypothetical protein | No defined Interpro term |
| MGG14825.6 | 4.04 | hypothetical protein | No defined Interpro term |
| MGG08194.6 | 4.04 | hypothetical protein | No defined Interpro term |
| MGG08920.6 | 4.03 | conserved hypothetical protein | No defined Interpro term |
| MGG13535.6 | 4.03 | nucleoporin POM152 | No defined Interpro term |
| MGG00882.6 | 4.02 | negative regulator of the PHO system | IPR013781: Glycoside hydrolase, subgroup, catalytic core |
| MGG08111.6 | 3.98 | conserved hypothetical protein | No defined Interpro term |
| MGG08781.6 | 3.97 | cupin domain-containing protein | IPR011051 : Cupin, RmlC-type, IPR013096 : Cupin 2, conserved barrel |
| MGG08173.6 | 3.96 | NADP-dependent malic enzyme | IPR012301: Malic enzyme, N-terminal, IPR012302: Malic enzyme, NAD-binding, IPR016040: NAD(P)-binding |
| MGG03597.6 | 3.95 | conserved hypothetical protein | IPR001680: WD40 repeat |
| MGG13635.6 | 3.95 | hypothetical protein | IPR017441: Protein kinase, ATP binding site |
| MGG11211.6 | 3.89 | GRAM domain-containing protein YSP2 | IPR004182: GRAM |
| MGG01753.6 | 3.88 | hypothetical protein | No defined Interpro term |
| MGG02692.6 | 3.88 | hypothetical protein | No defined Interpro term |
| MGG10928.6 | 3.87 | conserved hypothetical protein | No defined Interpro term |
| MGG02766.6 | 3.86 | succinate-semialdehyde dehydrogenase | IPR015590: Aldehyde dehydrogenase, IPR016161: Aldehyde/histidinol dehydrogenase |
| MGG02188.6 | 3.85 | glutamyl-tRNA(Gln) amidotransferase subunit A | IPR000120: Amidase signature enzyme |
| MGG07714.6 | 3.83 | conserved hypothetical protein | IPR017210: Uncharacterised conserved protein UCP037464, actin patch protein 1 |
| MGG15113.6 | 3.78 | pyridoxal reductase | IPR001395: Aldo/keto reductase |
| MGG08360.6 | 3.77 | DUF341 domain-containing protein | No defined Interpro term |
| MGG01473.6 | 3.76 | oxidoreductase 2-nitropropane dioxygenase family | IPR004136: 2-nitropropane dioxygenase, NPD |
| MGG00130.6 | 3.76 | conserved hypothetical protein | No defined Interpro term |
| MGG07053.6 | 3.76 | conserved hypothetical protein | IPR005654 : ATPase, AFG1-like |
| MGG13220.6 | 3.74 | hypothetical protein | No defined Interpro term |
| MGG07946.6 | 3.71 | conserved hypothetical protein | No defined Interpro term |
| MGG01357.6 | 3.71 | conserved hypothetical protein | No defined Interpro term |
| MGG11636.6 | 3.71 | conserved hypothetical protein | IPR000719 : Protein kinase, core, IPR001245 : Tyrosine protein kinase, IPR002110 : Ankyrin, IPR002290 : Serine/threonine protein kinase, IPR008271 : Serine/threonine protein kinase, active site, IPR011009 : Protein kinase-like, IPR017442 : Serine/threonine protein kinase-related |
| MGG09072.6 | 3.70 | alcohol oxidase | IPR012132 : Glucose-methanol-choline oxidoreductase |
| MGG07022.6 | 3.70 | hypothetical protein | IPR000210: BTB/POZ-like |
| MGG00126.6 | 3.67 | conserved hypothetical protein | IPR011043: Galactose oxidase/kelch, beta-propeller, IPR015915 : Kelch-type beta propeller |
| MGG01380.6 | 3.67 | 26S protease regulatory subunit 8 | IPR003593: ATPase, AAA+ type, core, IPR003959: ATPase, AAA-type, core, IPR005937: 26S proteasome subunit P45 |
| MGG03470.6 | 3.63 | hypothetical protein | No defined Interpro term |
| MGG01328.6 | 3.62 | endoglucanase | IPR000334: Glycoside hydrolase, family 45, IPR009009: Barwin-related endoglucanase |
| MGG01815.6 | 3.62 | spindle pole body component alp6 | IPR007259: Spc97/Spc98, IPR015697: Gamma tubulin complex protein 3 |
| MGG01725.6 | 3.62 | deoxyhypusine hydroxylase | IPR004155: PBS lyase HEAT-like repeat, IPR016024: Armadillo-type fold |
| MGG00786.6 | 3.62 | conserved hypothetical protein | No defined Interpro term |
| MGG14640.6 | 3.61 | mating-type switching protein swi10 | IPR003903: Ubiquitin interacting motif, IPR004579: DNA repair protein rad10 |
| MGG11286.6 | 3.60 | oxidoreductase | IPR006620: Prolyl 4-hydroxylase, alpha subunit |
| MGG11355.6 | 3.60 | hypothetical protein | No defined Interpro term |
| MGG01748.6 | 3.59 | conserved hypothetical protein | IPR001461: Peptidase A1, IPR009007: Peptidase aspartic, catalytic |
| MGG02614.6 | 3.59 | hypothetical protein | IPR011058: Cyanovirin-N |
| MGG02625.6 | 3.59 | superoxide dismutase | IPR001424: Superoxide dismutase, copper/zinc binding |
| MGG01771.6 | 3.58 | peroxisome assembly protein 12 | IPR006845: Pex, N-terminal, IPR013083 : Zinc finger, RING/FYVE/PHD-type |
| MGG13168.6 | 3.58 | conserved hypothetical protein | No defined Interpro term |
| MGG01356.6 | 3.57 | hypothetical protein | IPR013838: Beta tubulin, autoregulation binding site |
| MGG00332.6 | 3.57 | conserved hypothetical protein | IPR012479: HCNGP-like |
| MGG08883.6 | 3.56 | conserved hypothetical protein | No defined Interpro term |
| MGG10551.6 | 3.56 | hypothetical protein | No defined Interpro term |
| MGG04481.6 | 3.55 | hypothetical protein | No defined Interpro term |
| MGG07269.6 | 3.54 | conserved hypothetical protein | IPR007087: Zinc finger, C2H2-type, IPR013087: Zinc finger, C2H2-type/integrase, DNA-binding |
| MGG04234.6 | 3.52 | hexose transporter protein | IPR005828: General substrate transporter, IPR016196: Major facilitator superfamily, general substrate transporter |
| MGG02798.6 | 3.52 | short-chain dehydrogenase/reductase SDR | IPR002198: Short-chain dehydrogenase/reductase SDR, IPR016040: NAD(P)-binding |
| MGG09095.6 | 3.51 | alpha-L-arabinofuranosidase | IPR005193: Glycoside hydrolase, family 62, arabinosidase |
| MGG00273.6 | 3.51 | conserved hypothetical protein | No defined Interpro term |
| MGG01806.6 | 3.50 | conserved hypothetical protein | No defined Interpro term |
| MGG11813.6 | 3.49 | hypothetical protein | No defined Interpro term |
| MGG02797.6 | 3.48 | conserved hypothetical protein | No defined Interpro term |
| MGG07162.6 | 3.48 | sphingosine-1-phosphate lyase | IPR002129: Pyridoxal phosphate-dependent decarboxylase, IPR015421: Pyridoxal phosphate-dependent transferase, major region, subdomain 1 |
| MGG05890.6 | 3.46 | beta-galactosidase | IPR004199: Glycoside hydrolase, family 42, domain 5, TIM barrel, IPR011013: Glycoside hydrolase-type carbohydrate-binding, IPR013781: Glycoside hydrolase, subgroup, catalytic core |
| MGG03298.6 | 3.45 | conserved hypothetical protein | IPR011701 : Major facilitator superfamily MFS-1, IPR011701 : Major facilitator superfamily MFS-1, IPR016196 : Major facilitator superfamily, general substrate transporter, |
| MGG15100.6 | 3.45 | polyketide synthase | IPR013968: Polyketide synthase, KR, IPR000794: Beta-ketoacyl synthase, IPR001227: Acyl transferase region, IPR001242: Condensation domain, IPR006163: Phosphopantetheine-binding, IPR013217: Methyltransferase type 12 |
| MGG12141.6 | 3.44 | COPII coat assembly protein SEC16 | No defined Interpro term |
| MGG01272.6 | 3.43 | CORD and CS domain-containing protein | IPR007051: CHORD, IPR007052: CS domain |
| MGG09489.6 | 3.42 | conserved hypothetical protein | No defined Interpro term |
| MGG02246.6 | 3.42 | conserved hypothetical protein | No defined Interpro term |
| MGG00577.6 | 3.41 | conserved hypothetical protein | IPR011041: Soluble quinoprotein glucose dehydrogenase |
| MGG02698.6 | 3.39 | hypothetical protein | No defined Interpro term |
| MGG07245.6 | 3.39 | hypothetical protein | No defined Interpro term |
| MGG02413.6 | 3.39 | hypothetical protein | No defined Interpro term |
| MGG12521.6 | 3.38 | hypothetical protein | No defined Interpro term |
| MGG03176.6 | 3.37 | hypothetical protein | No defined Interpro term |
| MGG09461.6 | 3.37 | UV-damage endonuclease | IPR004601: UV-endonuclease UvdE |
| MGG09970.6 | 3.32 | hypothetical protein | No defined Interpro term |
| MGG01769.6 | 3.29 | conserved hypothetical protein | IPR007632: Protein of unknown function DUF590 |
| MGG00045.6 | 3.28 | general alpha-glucoside permease | IPR003663: Sugar/inositol transporter, IPR005828: General substrate transporter, IPR005829: Sugar transporter, conserved site, IPR016196: Major facilitator superfamily, general substrate transporter |
| MGG08741.6 | 3.28 | tRNA selenocysteine-associated protein 1 | IPR000504: RNA recognition motif, RNP-1, IPR012677: Nucleotide-binding, alpha-beta plait |
| MGG14633.6 | 3.27 | KpsF/GutQ family protein | IPR001199: Cytochrome b5, IPR001347 : Sugar isomerase (SIS) |
| MGG07265.6 | 3.27 | endosulphine family protein | IPR006760: cAMP-regulated phosphoprotein/endosulphine conserved region |
| MGG05959.6 | 3.27 | bZIP transcription factor | IPR004827: Basic-leucine zipper (bZIP) transcription factor |
| MGG00177.6 | 3.24 | conserved hypothetical protein | No defined Interpro term |
| MGG02610.6 | 3.22 | conserved hypothetical protein | IPR013094: Alpha/beta hydrolase fold-3 |
| MGG01843.6 | 3.20 | phosphatidylethanolamine-binding protein | IPR008914: Phosphatidylethanolamine-binding protein PEBP |
| MGG01256.6 | 3.19 | phosphoribosylaminoimidazole carboxylase | IPR016301: Phosphoribosylaminoimidazole carboxylase IPR000031: 1-(5-Phosphoribosyl)-5-amino-4-imidazole-carboxylate (AIR) carboxylase, IPR003135: ATP-grasp fold, ATP-dependent carboxylate-amine ligase-type, IPR011054: Rudiment single hybrid motif, IPR013816: ATP-grasp fold, subdomain 2, |
| MGG00184.6 | 3.19 | MoHOX2 | IPR001356: Homeobox |
| MGG04601.6 | 3.18 | conserved hypothetical protein | No defined Interpro term |
| MGG09740.6 | 3.17 | hypothetical protein | IPR011058: Cyanovirin-N |
| MGG00679.6 | 3.16 | conserved hypothetical protein | IPR013952: Protein of unknown function DUF1776, fungi IPR016040: NAD(P)-binding |
| MGG15053.6 | 3.15 | histone-lysine N-methyltransferase | IPR000834: Peptidase M14, carboxypeptidase A, IPR003616: Post-SET zinc-binding region, IPR012677: Nucleotide-binding, alpha-beta plait, IPR015722: MLL Transcription Factor |
| MGG10170.6 | 3.15 | conserved hypothetical protein | IPR010721: Protein of unknown function DUF1295 |
| MGG03326.6 | 3.14 | conserved hypothetical protein | No defined Interpro term |
| MGG11784.6 | 3.14 | conserved hypothetical protein | IPR001895: Guanine-nucleotide dissociation stimulator CDC25, IPR008937: Ras guanine nucleotide exchange factor |
| MGG05434.6 | 3.13 | acriflavine sensitivity control protein acr-2 | IPR001138: Fungal transcriptional regulatory protein, N-terminal |
| MGG13736.6 | 3.13 | hypothetical protein | No defined Interpro term |
| MGG10736.6 | 3.13 | hypothetical protein | No defined Interpro term |
| MGG06373.6 | 3.10 | oxidoreductase | IPR003042: Aromatic-ring hydroxylase-like |
| MGG08764.6 | 3.10 | hypothetical protein | No defined Interpro term |
| MGG15094.6 | 3.09 | acetylcholinesterase | IPR002018: Carboxylesterase, type B |
| MGG08254.6 | 3.09 | conserved hypothetical protein | IPR005103: Glycoside hydrolase, family 61 |
| MGG04441.6 | 3.08 | conserved hypothetical protein | No defined Interpro term |
| MGG02509.6 | 3.08 | juvenile hormone epoxide hydrolase 2 | IPR000073: Alpha/beta hydrolase fold-1, IPR016292: Epoxide hydrolase |
| MGG09359.6 | 3.07 | alcohol dehydrogenase | IPR002085: Alcohol dehydrogenase superfamily, zinc-containing, IPR011032: GroES-like, IPR016040: NAD(P)-binding |
| MGG07519.6 | 3.07 | hypothetical protein | No defined Interpro term |
| MGG06297.6 | 3.06 | conserved hypothetical protein | IPR001680: WD40 repeat |
| MGG06944.6 | 3.04 | zinc finger protein RTS2 | IPR007087: Zinc finger, C2H2-type |
| MGG06350.6 | 3.04 | hypothetical protein | IPR000637: HMG-I and HMG-Y, DNA-binding, conserved site |
| MGG02950.6 | 3.04 | conserved hypothetical protein | IPR009053: Prefoldin |
| MGG06103.6 | 3.04 | conserved hypothetical protein | No defined Interpro term |
| MGG03341.6 | 3.03 | galactose-proton symporter | IPR005828: General substrate transporter, IPR016196: Major facilitator superfamily, general substrate transporter |
| MGG01130.6 | 3.02 | conserved hypothetical protein | IPR001005: SANT, DNA-binding |
| MGG10362.6 | 3.02 | NAD/NADP octopine/nopaline dehydrogenase | IPR003421: Opine dehydrogenase, IPR011128: NAD-dependent glycerol-3-phosphate dehydrogenase, N-terminal, IPR013328: Dehydrogenase, multihelical, IPR016040: NAD(P)-binding |
| MGG08864.6 | 3.02 | hypothetical protein | IPR006058: 2Fe-2S ferredoxin, iron-sulphur binding site |
| MGG05318.6 | 3.02 | conserved hypothetical protein | IPR013925: Spindle pole body interacting protein, |
| MGG01659.6 | 3.02 | conserved hypothetical protein-SSY-ALL NORMANL | IPR000637: HMG-I and HMG-Y, DNA-binding, conserved site |
| MGG07136.6 | 3.01 | nucleolar GTP-binding protein 1 | IPR005225: Small GTP-binding protein, IPR006073: GTP1/OBG, IPR010674 : Nucleolar GTP-binding 1, IPR012973: NOG, C-terminal |
| MGG05287.6 | 3.01 | conserved hypothetical protein (CON7) |  |
| MGG03307.6 | 3.00 | hypothetical protein | IPR002482: Peptidoglycan-binding Lysin subgroup, IPR011058: Cyanovirin-N |
| MGG01331.6 | 2.99 | vacuolar protein sorting 53 | IPR007234: Vps53-like, N-terminal |
| MGG04312.6 | 2.98 | conserved hypothetical protein | No defined Interpro term |
| MGG09115.6 | 2.98 | hypothetical protein | No defined Interpro term |
| MGG02518.6 | 2.98 | ATP-dependent RNA helicase DHX8 | IPR001650: DNA/RNA helicase, C-terminal, IPR002464: DNA/RNA helicase, ATP-dependent, DEAH-box type, conserved site, IPR007502: Helicase-associated region, IPR014021: Helicase, superfamily 1 and 2, ATP-binding |
| MGG04126.6 | 2.98 | sulfate permease 2 | IPR001902: Sulphate anion transporter, IPR011547: Sulphate transporter |
| MGG08079.6 | 2.97 | rRNA processing protein Rrp17 | No defined Interpro term |
| MGG14655.6 | 2.97 | hypothetical protein | No defined Interpro term |
| MGG02783.6 | 2.96 | lactose permease | IPR005828: General substrate transporter, IPR005829: Sugar transporter, conserved site, IPR016196: Major facilitator superfamily, general substrate transporter |
| MGG07473.6 | 2.94 | glycosyl hydrolase |  |
| MGG10606.6 | 2.93 | aminopeptidase 2 | IPR001930: Peptidase M1, membrane alanine aminopeptidase, IPR014782: Peptidase M1, membrane alanine aminopeptidase, N-terminal, |
| MGG14731.6 | 2.93 | peroxisomal membrane protein 4 | IPR014467: Peroxisomal membrane protein 4 |
| MGG05328.6 | 2.92 | peroxin 8 | IPR008984: SMAD/FHA domain |
| MGG01390.6 | 2.91 | MFS hexose transporter | IPR003663: Sugar/inositol transporter, IPR005828: General substrate transporter, IPR005829: Sugar transporter, conserved site, IPR016196: Major facilitator superfamily, general substrate transporter |
| MGG06898.6 | 2.90 | Transcription factor | IPR015495: Myb transcription factor, IPR001005: SANT, DNA-binding, IPR017930: Myb-type HTH DNA-binding domain |
| MGG00618.6 | 2.90 | pectinesterase | IPR000070: Pectinesterase, catalytic, IPR011050: Pectin lyase fold/virulence factor |
| MGG05695.6 | 2.90 | conserved hypothetical protein | IPR000223: Peptidase S26A, signal peptidase I |
| MGG07764.6 | 2.89 | conserved hypothetical protein | IPR011701: Major facilitator superfamily MFS-1, IPR016196: Major facilitator superfamily, general substrate transporter |
| MGG05437.6 | 2.89 | conserved hypothetical protein | No defined Interpro term |
| MGG01925.6 | 2.88 | bifunctional P-450 NADPH-P450 reductase | IPR001128: Cytochrome P450, IPR001433: Oxidoreductase FAD/NAD(P)-binding, IPR003097: FAD-binding, type 1, IPR008254: Flavodoxin/nitric oxide synthase, IPR015702: NADPH Cytochrome P450 Reductase, IPR017927: Ferredoxin reductase-type FAD-binding domain |
| MGG12005.6 | 2.88 | cation-transporting ATPase 4 | IPR001757: ATPase, P-type, K/Mg/Cd/Cu/Zn/Na/Ca/Na/H-transporter, IPR005834: Haloacid dehalogenase-like hydrolase, IPR008250: ATPase, P-type, ATPase-associated region |
| MGG10067.6 | 2.88 | conserved hypothetical protein | No defined Interpro term |
| MGG09129.6 | 2.87 | conserved hypothetical protein | No defined Interpro term |
| MGG00951.6 | 2.87 | mannan endo-1,6-alpha-mannosidase DCW1 | IPR005198: Glycoside hydrolase, family 76, IPR014480: Mannan endo-1,6-alpha-mannosidase |
| MGG09512.6 | 2.87 | 3-ketoacyl-CoA thiolase | IPR002155: Thiolase,IPR002155 : Thiolase |
| MGG02376.6 | 2.86 | hormone-sensitive lipase | IPR002168: Lipase, GDXG, active site, IPR013094: Alpha/beta hydrolase fold-3 |
| MGG09199.6 | 2.84 | repressible acid phosphatase | IPR000560: Histidine acid phosphatase |
| MGG03705.6 | 2.84 | SH3 domain-containing protein | IPR001452: Src homology-3 domain |
| MGG03555.6 | 2.84 | hypothetical protein | No defined Interpro term |
| MGG07214.6 | 2.83 | conserved hypothetical protein |  |
| MGG09428.6 | 2.82 | conserved hypothetical protein | No defined Interpro term |
| MGG12108.6 | 2.82 | leucine Rich Repeat domain-containing protein |  |
| MGG01916.6 | 2.82 | hypothetical protein | No defined Interpro term |
| MGG01922.6 | 2.81 | polysaccharide deacetylase family protein | IPR002509: Polysaccharide deacetylase, IPR011330: Glycoside hydrolase/deacetylase, beta/alpha-barrel |
| MGG03585.6 | 2.81 | hypothetical protein | No defined Interpro term |
| MGG01364.6 | 2.80 | conserved hypothetical protein | No defined Interpro term |
| MGG03436.6 | 2.80 | hypothetical protein | No defined Interpro term |
| MGG13465.6 | 2.79 | conserved hypothetical protein | No defined Interpro term |
| MGG10004.6 | 2.78 | hypothetical protein | No defined Interpro term |
| MGG10868.6 | 2.77 | conserved hypothetical protein | No defined Interpro term |
| MGG08043.6 | 2.76 | hypothetical protein | No defined Interpro term |
| MGG02089.6 | 2.76 | hypothetical protein | No defined Interpro term |
| MGG00322.6 | 2.76 | chromosome 9 open reading frame 41 | IPR012901: N2227-like |
| MGG11611.6 | 2.75 | conserved hypothetical protein | IPR002293: Amino acid/polyamine transporter I, IPR004841 : Amino acid permease-associated region |
| MGG08500.6 | 2.75 | pisatin demethylase | IPR001128: Cytochrome P450 |
| MGG00501.6 | 2.74 | C2H2 zinc finger | IPR007087: Zinc finger, C2H2-type, IPR013087: Zinc finger, C2H2-type/integrase, DNA-binding |
| MGG08143.6 | 2.73 | thiamine-repressible mitochondrial transport protein THI74 | IPR000620: Protein of unknown function DUF6, transmembrane |
| MGG01701.6 | 2.73 | geranylgeranyl pyrophosphate synthetase | IPR000092: Polyprenyl synthetase, IPR008949: Terpenoid synthase |
| MGG03130.6 | 2.72 | pps1 dual specificty phosphatase | IPR000340: Protein-tyrosine phosphatase, dual specificity, IPR000387: Protein-tyrosine phosphatase |
| MGG07654.6 | 2.72 | prolyl peptidase | IPR000073: Alpha/beta hydrolase fold-1 |
| MGG06168.6 | 2.71 | conserved hypothetical protein | No defined Interpro term |
| MGG07781.6 | 2.70 | quinate dehydrogenase | IPR006151: Quinate/shikimate 5-dehydrogenase/glutamyl-tRNA reductase, IPR013708: Shikimate dehydrogenase substrate binding, N-terminal IPR016040: NAD(P)-binding |
| MGG13933.6 | 2.70 | hypothetical protein | No defined Interpro term |
| MGG05206.6 | 2.70 | conserved hypothetical protein | IPR000194: ATPase, F1/V1/A1 complex, alpha/beta subunit, nucleotide-binding |
| MGG05022.6 | 2.69 | conserved hypothetical protein | IPR008030: NmrA-like, IPR016040: NAD(P)-binding |
| MGG15294.6 | 2.66 | proline iminopeptidase | IPR000073: Alpha/beta hydrolase fold-1, IPR010497: Epoxide hydrolase, N-terminal, IPR016292: Epoxide hydrolase |
| MGG08815.6 | 2.64 | conserved hypothetical protein | IPR007248: Mpv17/PMP22, |
| MGG00886.6 | 2.63 | hypothetical protein | No defined Interpro term |
| MGG13511.6 | 2.63 | transcription elongation factor S-II | IPR001222: Zinc finger, TFIIS-type |
| MGG_03516.6 | 2.63 | transcription factor prr1 | IPR000232: Heat shock factor (HSF)-type, DNA-binding, IPR001789: Signal transduction response regulator, receiver region, IPR011006: CheY-like, IPR011991: Winged helix repressor DNA-binding |
| MGG15143.6 | 2.63 | hypothetical protein | No defined Interpro term |
| MGG04662.6 | 2.62 | conserved hypothetical protein | IPR001993: Mitochondrial substrate carrier |
| MGG06285.6 | 2.61 | MoHOX4 | IPR001356: Homeobox |
| MGG05802.6 | 2.61 | conserved hypothetical protein | No defined Interpro term |
| MGG04605.6 | 2.61 | cytochrome P450 3A12 | IPR001128: Cytochrome P450 |
| MGG09798.6 | 2.61 | conserved hypothetical protein | No defined Interpro term |
| MGG12243.6 | 2.60 | conserved hypothetical protein | No defined Interpro term |
| MGG05525.6 | 2.59 | conserved hypothetical protein | IPR007913: Uncharacterised protein family UPF0187 |
| MGG06005.6 | 2.58 | RNA exonuclease 3 | IPR006055: Exonuclease, IPR012337: Polynucleotidyl transferase, Ribonuclease H fold, IPR013520: Exonuclease, RNase T and DNA polymerase III |
| MGG03603.6 | 2.57 | conserved hypothetical protein | IPR004127: Prefoldin alpha-like, IPR009053: Prefoldin |
| MGG03132.6 | 2.57 | DNA repair helicase RAD3 | IPR002464: DNA/RNA helicase, ATP-dependent, DEAH-box type, conserved site, IPR014013: Helicase, superfamily 1 and 2, ATP-binding, DinG/Rad3-type |
| MGG13278.6 | 2.57 | hypothetical protein | No defined Interpro term |
| MGG02730.6 | 2.57 | RNA binding protein | IPR000504: RNA recognition motif, RNP-1, IPR012677: Nucleotide-binding, alpha-beta plait |
| MGG05080.6 | 2.57 | hypothetical protein | No defined Interpro term |
| MGG00763.6 | 2.56 | NIMA interactive protein | No defined Interpro term |
| MGG09197.6 | 2.56 | hypothetical protein | No defined Interpro term |
| MGG09368.6 | 2.56 | hypothetical protein | No defined Interpro term |
| MGG09026.6 | 2.55 | conserved hypothetical protein | IPR007526: SWIRM |
| MGG06929.6 | 2.55 | conserved hypothetical protein | No defined Interpro term |
| MGG05479.6 | 2.55 | xylosidase/arabinosidase | IPR006710: Glycoside hydrolase, family 43 |
| MGG08617.6 | 2.55 | H(+)/hexose cotransporter 1 | IPR003663: Sugar/inositol transporter, IPR005828: General substrate transporter, IPR005829: Sugar transporter, conserved site, IPR016196: Major facilitator superfamily, general substrate transporter |
| MGG03448.6 | 2.55 | golgi transport complex component Cog5 | No defined Interpro term |
| MGG11693.6 | 2.54 | conserved hypothetical protein | IPR000342: Regulator of G protein signalling, IPR016137: Regulator of G protein signalling superfamily |
| MGG11903.6 | 2.54 | cation efflux family protein family | IPR002524: Cation efflux protein |
| MGG01624.6 | 2.54 | fungal specific transcription factor domain-containing protein | IPR001138: Fungal transcriptional regulatory protein, N-terminal, IPR007219: Fungal specific transcription factor |
| MGG00019.6 | 2.53 | conserved hypothetical protein | IPR001214: SET |
| MGG03892.6 | 2.53 | conserved hypothetical protein | No defined Interpro term |
| MGG03173.6 | 2.52 | conserved hypothetical protein | IPR003593: ATPase, AAA+ type, core, IPR010339: TIP49, C-terminal |
| MGG01615.6 | 2.52 | pH-response regulator protein palF/rim-8 | IPR011021 : Arrestin-like, N-terminal, IPR011022 : Arrestin-like, C-terminal, IPR014756 : Immunoglobulin E-set |
| MGG03117.6 | 2.51 | alpha-ketoglutarate-dependent taurine dioxygenase | IPR003819 : Taurine catabolism dioxygenase TauD/TfdA |
| MGG06866.6 | 2.50 | conserved hypothetical protein | No defined Interpro term |
| MGG04680.6 | 2.49 | lipase 2 | IPR013094: Alpha/beta hydrolase fold-3 |
| MGG08278.6 | 2.49 | conserved hypothetical protein | IPR001433: Oxidoreductase FAD/NAD(P)-binding, IPR013112: FAD-binding 8, IPR013130: Ferric reductase-like transmembrane component, N-terminal, IPR017927: Ferredoxin reductase-type FAD-binding domain |
| MGG05176.6 | 2.49 | mitochondrial outer membrane protein involved in mitochondrial shape | No defined Interpro term |
| MGG03055.6 | 2.49 | hypothetical protein | IPR001138: Fungal transcriptional regulatory protein, N-terminal |
| MGG05927.6 | 2.49 | conserved hypothetical protein | No defined Interpro term |
| MGG08326.6 | 2.48 | conserved hypothetical protein | IPR006353: HAD-superfamily hydrolase, subfamily IIA, CECR5, IPR006357 : HAD-superfamily hydrolase, subfamily IIA |
| MGG14215.6 | 2.48 | pyridoxal-5'-phosphate-dependent protein beta subunit | IPR000277: Cys/Met metabolism, pyridoxal phosphate-dependent enzyme |
| MGG04522.6 | 2.48 | conserved hypothetical protein | No defined Interpro term |
| MGG02748.6 | 2.48 | RNA-dependent RNA polymerase 1 | IPR007855: RNA-dependent RNA polymerase, eukaryotic-type |
| MGG01768.6 | 2.48 | hypothetical protein | IPR001452: Src homology-3 domain |
| MGG01480.6 | 2.47 | conserved hypothetical protein | IPR001185: Large-conductance mechanosensitive channel |
| MGG00837.6 | 2.47 | conserved hypothetical protein | IPR016160: Aldehyde dehydrogenase, conserved site |
| MGG05683.6 | 2.46 | activator of stress genes | IPR007219: Fungal specific transcription factor |
| MGG00916.6 | 2.46 | hypothetical protein | IPR007087: Zinc finger, C2H2-type, IPR015880 : Zinc finger, C2H2-like |
| MGG00952.6 | 2.45 | amidohydrolase 2 | IPR006992: Amidohydrolase 2 |
| MGG03533.6 | 2.45 | formamidase | IPR004304: Acetamidase/Formamidase |
| MGG01001.6 | 2.45 | endo-1,3(4)-beta-glucanase 1 precursor | IPR002345: Lipocalin, IPR005200 : Glycoside hydrolase, family 81 |
| MGG01503.6 | 2.44 | hypothetical protein | No defined Interpro term |
| MGG08751.6 | 2.44 | hypothetical protein | No defined Interpro term |
| MGG03384.6 | 2.43 | conserved hypothetical protein | No defined Interpro term |
| MGG11885.6 | 2.43 | conserved hypothetical protein | IPR000873: AMP-dependent synthetase and ligase |
| MGG11385.6 | 2.43 | conserved hypothetical protein | IPR016125: Peptidase C15, pyroglutamyl peptidase I-like |
| MGG01617.6 | 2.43 | telomerase reverse transcriptase | IPR000477: RNA-directed DNA polymerase (reverse transcriptase) |
| MGG09008.6 | 2.43 | conserved hypothetical protein | No defined Interpro term |
| MGG02157.6 | 2.42 | hypothetical protein | No defined Interpro term |
| MGG02088.6 | 2.41 | conserved hypothetical protein | IPR013922: Cyclin-related 2 |
| MGG14661.6 | 2.41 | hypothetical protein | No defined Interpro term |
| MGG10531.6 | 2.40 | hypothetical protein | No defined Interpro term |
| MGG01029.6 | 2.40 | acetyl-hydrolase | IPR013094: Alpha/beta hydrolase fold-3 |
| MGG13935.6 | 2.40 | conserved hypothetical protein |  |
| MGG06710.6 | 2.39 | conserved hypothetical protein | IPR007175: RNAse P, Rpr2/Rpp21 subunit |
| MGG10038.6 | 2.38 | periplasmic beta-glucosidase/beta-xylosidase | IPR001764: Glycoside hydrolase, family 3, N-terminal, IPR002772: Glycoside hydrolase, family 3, C-terminal, IPR017853: Glycoside hydrolase, catalytic core |
| MGG04779.6 | 2.37 | conserved hypothetical protein | No defined Interpro term |
| MGG04176.6 | 2.37 | conserved hypothetical protein | IPR010730: Heterokaryon incompatibility |
| MGG01043.6 | 2.37 | catabolite repression protein creC | IPR001680: WD40 repeat, IPR011046 : WD40 repeat-like, IPR015943 : WD40/YVTN repeat-like |
| MGG00645.6 | 2.37 | phosphopantothenoylcysteine decarboxylase | IPR003382: Flavoprotein |
| MGG06651.6 | 2.36 | BTB/POZ domain-containing protein | IPR011333: BTB/POZ fold |
| MGG14864.6 | 2.36 | hypothetical protein | No defined Interpro term |
| MGG03233.6 | 2.36 | ATP-dependent DNA helicase recQ | IPR001650: DNA/RNA helicase, C-terminal, IPR002464: DNA/RNA helicase, ATP-dependent, DEAH-box type, conserved site, IPR004589: DNA helicase, ATP-dependent, RecQ type, IPR014021: Helicase, superfamily 1 and 2, ATP-binding |
| MGG06007.6 | 2.35 | hypothetical protein | No defined Interpro term |
| MGG03701.6 | 2.35 | conserved hypothetical protein | IPR011701: Major facilitator superfamily MFS-1, IPR016196: Major facilitator superfamily, general substrate transporter |
| MGG06747.6 | 2.34 | glutathione S-transferase | IPR004045: Glutathione S-transferase, N-terminal, IPR004046: Glutathione S-transferase, C-terminal, IPR012335: Thioredoxin fold, IPR017933: Glutathione S-transferase/chloride channel, C-terminal |
| MGG00275.6 | 2.34 | MFS transporter | IPR011701: Major facilitator superfamily MFS-1, IPR016196: Major facilitator superfamily, general substrate transporter, |
| MGG07000.6 | 2.34 | ribonucleoside-diphosphate reductase large chain | IPR000788: Ribonucleotide reductase large subunit, C-terminal, IPR005144: ATP-cone, IPR008926: Ribonucleotide reductase R1 subunit, N-terminal |
| MGG06548.6 | 2.34 | carotenoid oxygenase | IPR004294: Carotenoid oxygenase |
| MGG05343.6 | 2.33 | C6 zinc finger protein | IPR001138: Fungal transcriptional regulatory protein, N-terminal, IPR007219: Fungal specific transcription factor |
| MGG15038.6 | 2.33 | glycine dehydrogenase | IPR003437: Glycine cleavage system P-protein, IPR015421: Pyridoxal phosphate-dependent transferase, major region, subdomain 1 |
| MGG02880.6 | 2.33 | transcriptional activator xlnR | IPR007219: Fungal specific transcription factor |
| MGG03532.6 | 2.33 | ubiquitin thioesterase OTU1 | IPR003323: Ovarian tumour, otubain |
| MGG07075.6 | 2.32 | ATPase family AAA domain-containing protein 1-A | IPR003593: ATPase, AAA+ type, core, IPR003959 : ATPase, AAA-type, core |
| MGG07593.6 | 2.32 | isotrichodermin C-15 hydroxylase | IPR001128: Cytochrome P450 |
| MGG04895.6 | 2.31 | isocitrate lyase | IPR006254 : Isocitrate lyase, IPR000918: Isocitrate lyase and phosphorylmutase, IPR015813: Pyruvate/Phosphoenolpyruvate kinase, catalytic core |
| MGG01312.6 | 2.31 | conserved hypothetical protein | IPR003006: Immunoglobulin/major histocompatibility complex, conserved site |
| MGG08409.6 | 2.30 | cellulose-growth-specific protein | IPR000254: Cellulose-binding region, IPR005103: Glycoside hydrolase, family 61 |
| MGG07431.6 | 2.30 | conserved hypothetical protein | IPR001638: Extracellular solute-binding protein, family 3 |
| MGG04184.6 | 2.29 | PBSP domain-containing protein | IPR007541: Plant Basic Secretory Protein |
| MGG08120.6 | 2.29 | integral membrane protein | IPR005018: DOMON related, IPR006593: Cytochrome b561/ferric reductase transmembrane, IPR006593: Cytochrome b561/ferric reductase transmembrane, IPR008960: Carbohydrate-binding family 9/cellobiose dehydrogenase, cytochrome, IPR015920: Cellobiose dehydrogenase, cytochrome |
| MGG02438.6 | 2.29 | hypothetical protein | No defined Interpro term |
| MGG05875.6 | 2.29 | pectate lyase | IPR004898: Pectate lyase, catalytic, IPR011050: Pectin lyase fold/virulence factor |
| MGG08024.6 | 2.28 | hypothetical protein | No defined Interpro term |
| MGG00347.6 | 2.28 | N-glycosylase/DNA lyase | IPR003265: HhH-GPD domain, IPR011257 : DNA glycosylase, IPR012294: Transcription factor TFIID, C-terminal/DNA glycosylase, N-terminal, IPR012904: 8-oxoguanine DNA glycosylase, N-terminal |
| MGG06326.6 | 2.28 | vacuolar ATP synthase 16 kDa proteolipid subunit | IPR002379: ATPase, F0/V0 complex, subunit C, IPR011555: ATPase, V0 complex, proteolipid subunit C, eukaryotic |
| MGG03665.6 | 2.28 | tRNA wybutosine-synthesizing protein 3 | IPR003827: tRNA wybutosine-synthesizing protein, IPR006162: Phosphopantetheine attachment site |
| MGG10738.6 | 2.27 | mitochondrial chaperone BCS1 | IPR003593: ATPase, AAA+ type, core, IPR003959 : ATPase, AAA-type, core, IPR003960 : ATPase, AAA-type, conserved site, IPR014851 : BCS1, N-terminal |
| MGG04188.6 | 2.27 | conserved hypothetical protein | No defined Interpro term |
| MGG04607.6 | 2.27 | ornithine cyclodeaminase/mu-crystallin family protein | IPR003462: Ornithine cyclodeaminase/mu-crystallin, IPR016040: NAD(P)-binding |
| MGG08462.6 | 2.26 | hypothetical protein | No defined Interpro term |
| MGG01734.6 | 2.26 | C6 zinc finger domain-containing protein | IPR001138: Fungal transcriptional regulatory protein |
| MGG13615.6 | 2.25 | aquaporin-9 | IPR000425: Major intrinsic protein, IPR012269: Aquaporin |
| MGG09546.6 | 2.24 | conserved hypothetical protein | No defined Interpro term |
| MGG11544.6 | 2.24 | hypothetical protein | No defined Interpro term |
| MGG05218.6 | 2.24 | conserved hypothetical protein | No defined Interpro term |
| MGG09762.6 | 2.23 | conserved hypothetical protein | IPR001547: Glycoside hydrolase, family 5, IPR002035: von Willebrand factor, type A, |
| MGG00056.6 | 2.22 | xanthoxin dehydrogenase | IPR002198: Short-chain dehydrogenase/reductase SDR, IPR016040: NAD(P)-binding |
| MGG08377.6 | 2.22 | conserved hypothetical protein | IPR001077: O-methyltransferase, family 2, IPR011991: Winged helix repressor DNA-binding |
| MGG07335.6 | 2.21 | cAMP-dependent protein kinase regulatory subunit | IPR000595: Cyclic nucleotide-binding, IPR002373: cAMP/cGMP-dependent protein kinase, IPR012198 : cAMP-dependent protein kinase regulatory subunit |
| MGG00086.6 | 2.20 | 42 kDa endochitinase | IPR001223: Glycoside hydrolase, family 18, catalytic domain, IPR001579: Glycoside hydrolase, chitinase active site, IPR011583: Chitinase II |
| MGG13518.6 | 2.20 | sorbose reductase SOU1 | IPR002198: Short-chain dehydrogenase/reductase SDR, IPR002347: Glucose/ribitol dehydrogenase, IPR016040: NAD(P)-binding |
| MGG08922.6 | 2.19 | conserved hypothetical protein | No defined Interpro term |
| MGG00677.6 | 2.19 | endoglucanase-1 | IPR002594: Glycoside hydrolase, family 12, IPR008985 : Concanavalin A-like lectin/glucanase |
| MGG02926.6 | 2.19 | hypothetical protein | No defined Interpro term |
| MGG05247.6 | 2.18 | NAD-specific glutamate dehydrogenase | IPR006096: Glutamate/phenylalanine/leucine/valine dehydrogenase, C-terminal, IPR016040: NAD(P)-binding, IPR016210: Glutamate dehydrogenase, NAD-dependent |
| MGG05804.6 | 2.17 | phospholipase D p1 | IPR001736: Phospholipase D/Transphosphatidylase, IPR015679: Phospholipase D |
| MGG00459.6 | 2.15 | conserved hypothetical protein | IPR001104: 3-oxo-5-alpha-steroid 4-dehydrogenase, C-terminal, IPR010721: Protein of unknown function DUF1295 |
| MGG00102.6 | 2.15 | cell cycle control protein cwf19 | IPR006767: Protein similar to CwfJ, C-terminal 2, IPR006768: Protein similar to CwfJ, C-terminal 1, IPR011151 : Histidine triad motif |
| MGG03310.6 | 2.15 | T-complex protein 1 subunit eta | IPR002423: Chaperonin Cpn60/TCP-1, IPR012720: T-complex protein 1, eta subunit |
| MGG00157.6 | 2.15 | peroxisomal membrane protein PAS20 | IPR001452: Src homology-3 domain, IPR002016: Haem peroxidase, plant/fungal/bacterial, IPR007223: Peroxin 13, N-terminal, IPR011511: Variant SH3 |
| MGG00203.6 | 2.13 | conserved hypothetical protein | IPR000719: Protein kinase, core |
| MGG11326.6 | 2.13 | protein kinase domain-containing protein ppk38 | IPR000719: Protein kinase, core, IPR001245 Tyrosine protein kinase, IPR002290: Serine/threonine protein kinase |
| MGG01123.6 | 2.12 | ribosomal RNA-processing protein 12 | IPR000357: HEAT, IPR012978: Region of unknown function, NUC173, IPR016024: Armadillo-type fold |
| MGG04674.6 | 2.12 | conserved hypothetical protein | IPR001138: Fungal transcriptional regulatory protein, N-terminal |
| MGG10484.6 | 2.12 | conserved hypothetical protein | No defined Interpro term |
| MGG02801.6 | 2.12 | conserved hypothetical protein | No defined Interpro term |
| MGG03956.6 | 2.11 | conserved hypothetical protein | IPR003195: Transcription initiation factor IID, 18 kDa subunit, IPR009072: Histone-fold |
| MGG09402.6 | 2.11 | conserved hypothetical protein | No defined Interpro term |
| MGG04611.6 | 2.11 | carbonic anhydrase | IPR001765: Carbonic anhydrase, IPR015892: Carbonic anhydrase, prokaryotic-like, conserved site |
| MGG07517.6 | 2.11 | conserved hypothetical protein | IPR000700: PAS-associated, C-terminal |
| MGG07809.6 | 2.10 | exoglucanase 1 | IPR001722: Glycoside hydrolase, family 7, IPR008985: Concanavalin A-like lectin/glucanase |
| MGG12582.6 | 2.08 | hypothetical protein | No defined Interpro term |
| MGG12832.6 | 2.08 | sorting nexin-41 | IPR001683: Phox-like |
| MGG00965.6 | 2.07 | MSF1 domain-containing protein | IPR006797: PRELI/MSF1 |
| MGG08308.6 | 2.07 | conserved hypothetical protein | IPR012578: Nuclear pore complex component |
| MGG05288.6 | 2.07 | conserved hypothetical protein | IPR007946: AAR2 |
| MGG10251.6 | 2.07 | sulfate permease | IPR011547 : Sulphate transporter, IPR003583: Helix-hairpin-helix DNA-binding motif, class 1 |
| MGG03501.6 | 2.07 | conserved hypothetical protein | IPR010686: Protein of unknown function DUF1264, |
| MGG00688.6 | 2.06 | hypothetical protein | No defined Interpro term |
| MGG06883.6 | 2.06 | t-SNARE affecting a late Golgi compartment protein 2 | IPR000727: Target SNARE coiled-coil region, IPR006011: Syntaxin, N-terminal, IPR006012: Syntaxin/epimorphin, conserved site, IPR010989: t-SNARE |
| MGG12268.6 | 2.05 | transcription elongation factor spt-5 | IPR005100: Supt5 repeat, IPR005824 : KOW, IPR005825: Ribosomal protein L24/L26, conserved site, IPR017071: Transcription elongation factor Spt5 |
| MGG09780.6 | 2.05 | hypothetical protein | IPR001138: Fungal transcriptional regulatory protein, N-terminal, IPR007087: Zinc finger, C2H2-type, IPR013087: Zinc finger, C2H2-type/integrase, DNA-binding |
| MGG00912.6 | 2.03 | protein-tyrosine phosphatase 2 | IPR000387: Protein-tyrosine phosphatase, IPR003595: Protein-tyrosine phosphatase, catalytic |
| MGG09949.6 | 2.02 | oxysterol-binding protein | IPR000648: Oxysterol-binding protein |
| MGG06251.6 | 2.02 | conserved hypothetical protein | No defined Interpro term |
| MGG13127.6 | 2.02 | conserved hypothetical protein | No defined Interpro term |
| MGG07123.6 | 2.02 | ATP-dependent protease La 2 | IPR001984: Peptidase S16, Lon protease, C-terminal region, IPR003593: ATPase, AAA+ type, core, IPR003959 : ATPase, AAA-type, core, IPR004815 : Peptidase S16, ATP-dependent protease La |
| MGG03266.6 | 2.01 | conserved hypothetical protein | IPR002678: NGG1p interacting factor 3, NIF3,IPR002678: NGG1p interacting factor 3, NIF3 |
| MGG13290.6 | 2.01 | alpha/beta hydrolase | IPR000073: Alpha/beta hydrolase fold-1,IPR000073 : Alpha/beta hydrolase fold-1 |
| MGG03520.6 | 2.01 | zinc finger protein | IPR007087: Zinc finger, C2H2-type |
| MGG08307.6 | 2.01 | conserved hypothetical protein | IPR000873: AMP-dependent synthetase and ligase |
| MGG07475.6 | 2.01 | conserved hypothetical protein | IPR000387: Protein-tyrosine phosphatase, IPR016130: Protein-tyrosine phosphatase, active site |
| MGG03185.6 | 2.00 | ATP synthase subunit beta | IPR000194: ATPase, F1/V1/A1 complex, alpha/beta subunit, nucleotide-binding, IPR005722: ATPase, F1 complex, beta subunit |
| MGG09821.6 | 2.00 | conserved hypothetical protein | IPR011701: Major facilitator superfamily MFS-1, IPR016196: Major facilitator superfamily, general substrate transporter |
| MGG01355.6 | 2.00 | conserved hypothetical protein | No defined Interpro term |

^a^ Induction ratios from the microarray analysis are calculated as the expression in conidiating mycelia (CNMY) divided by non-conidiating mycelia (NCMY) of the wild-type.
