# Supplementary material for: Gene Expression Profiling during Conidiation in the Rice Blast Pathogen Magnaporthe oryzae
Source: PLoS One. 2012 Aug 21;7(8):e43202. doi: 10.1371/journal.pone.0043202 (PMC3424150; doi:10.1371/journal.pone.0043202)
Supplement: Table S4 — Genes repressed during conidiation of M. oryzae. (DOCX) [file pone.0043202.s004.docx]

**Table S4**. Genes repressed during conidiation of *M. oryzae*

| **Locus** | **Fold-reduction during conidiation in the wild-type^a^** | **Annotation** | **InterPro domain search** |
| --- | --- | --- | --- |
| MGG12983.6 | 0.50 | conserved hypothetical protein | IPR002198: Short-chain dehydrogenase/reductase SDR, IPR002347 : Glucose/ribitol dehydrogenase |
| MGG13517.6 | 0.50 | hypothetical protein | No defined Interpro term |
| MGG00672.6 | 0.50 | C6 zinc finger domain-containing protein | IPR001138: Fungal transcriptional regulatory protein, N-terminal |
| MGG08072.6 | 0.50 | cholesterol oxidase | IPR000172: Glucose-methanol-choline oxidoreductase, IPR003953: Fumarate reductase/succinate dehydrogenase flavoprotein, N-terminal |
| MGG07446.6 | 0.50 | conserved hypothetical protein | IPR012908 : PGAP1-like,IPR012908 : PGAP1-like |
| MGG08063.6 | 0.50 | pyruvate kinase | IPR001697: Pyruvate kinase, IPR015793: Pyruvate kinase, barrel, barrel, IPR015794: Pyruvate kinase, alpha/beta, IPR015813: Pyruvate/Phosphoenolpyruvate kinase, catalytic core |
| MGG06459.6 | 0.50 | heat shock protein HSP98 | IPR001270: Chaperonin clpA/B, IPR003593: ATPase, AAA+ type, core, IPR013093: ATPase associated with various cellular activities, AAA-2 |
| MGG01982.6 | 0.50 | conserved hypothetical protein | IPR006598 : Lipopolysaccharide-modifying protein, |
| MGG00799.6 | 0.50 | peptide-N4-(N-acetyl-beta-glucosaminyl)asparagine amidase A | No defined Interpro term |
| MGG13192.6 | 0.50 | conserved hypothetical protein | No defined Interpro term |
| MGG07482.6 | 0.50 | conserved hypothetical protein | No defined Interpro term |
| MGG05061.6 | 0.49 | conserved hypothetical protein | IPR000120: Amidase signature enzyme |
| MGG12967.6 | 0.49 | hypothetical protein | No defined Interpro term |
| MGG03667.6 | 0.49 | conserved hypothetical protein | IPR013112: FAD-binding 8, IPR013121: Ferric reductase, NAD binding, IPR013130: Ferric reductase-like transmembrane component, N-terminal |
| MGG08693.6 | 0.49 | topoisomerase II | No defined Interpro term |
| MGG00576.6 | 0.49 | conserved hypothetical protein | No defined Interpro term |
| MGG10328.6 | 0.49 | allantoate permease | IPR011701: Major facilitator superfamily MFS-1 |
| MGG04706.6 | 0.49 | hypothetical protein | No defined Interpro term |
| MGG08019.6 | 0.49 | F-box domain-containing protein | IPR001810: Cyclin-like F-box |
| MGG09240.6 | 0.49 | F-box domain-containing protein | IPR001810: Cyclin-like F-box |
| MGG12109.6 | 0.49 | conserved hypothetical protein | IPR001965: Zinc finger, PHD-type,PHD-type |
| MGG00705.6 | 0.49 | hypothetical protein | No defined Interpro term |
| MGG01452.6 | 0.49 | sexual differentiation process protein isp4 | IPR004648: Tetrapeptide transporter, OPT1/isp4, IPR004813: Oligopeptide transporter OPT superfamily |
| MGG15085.6 | 0.48 | conserved hypothetical protein | IPR001138: Fungal transcriptional regulatory protein, N-terminal ,IPR007219 : Fungal specific transcription factor |
| MGG06648.6 | 0.48 | hypoxia up-regulated protein 1 precursor | IPR001023: Heat shock protein Hsp70 |
| MGG00698.6 | 0.48 | conserved hypothetical protein | IPR008854: Thiopurine S-methyltransferase |
| MGG13427.6 | 0.48 | NAD/NADP octopine/nopaline dehydrogenase | IPR003421: Opine dehydrogenase, IPR00892: 6-phosphogluconate dehydrogenase, C-terminal-like |
| MGG14004.6 | 0.48 | ribonuclease H | IPR002156: Ribonuclease H, IPR009027: Ribosomal protein L9 N-terminal-like |
| MGG05674.6 | 0.48 | non-imprinted in Prader-Willi/Angelman syndrome region protein 2 | IPR008521 : Protein of unknown function DUF803 |
| MGG07101.6 | 0.48 | mannan endo-1,6-alpha-mannosidase DCW1 precursor | IPR005198: Glycoside hydrolase, family 76, IPR008928: Six-hairpin glycosidase-like, IPR014480: Mannan endo-1,6-alpha-mannosidase |
| MGG08613.6 | 0.48 | poly polymerase 2 ADP-ribosyltransferase 2 | IPR001357: BRCT, IPR004102: Poly(ADP-ribose) polymerase, regulatory region |
| MGG11860.6 | 0.47 | NACHT and WD domain-containing protein | IPR011046: WD40 repeat-like, IPR015943: WD40/YVTN repeat-like |
| MGG09920.6 | 0.47 | cytochrome P450 52A5 | IPR001128: Cytochrome P450, IPR002401: Cytochrome P450, E-class, group I |
| MGG05506.6 | 0.47 | glyoxylate reductase | IPR006140 : D-isomer specific 2-hydroxyacid dehydrogenase, NAD-binding |
| MGG00321.6 | 0.47 | conserved hypothetical protein | No defined Interpro term |
| MGG09719.6 | 0.47 | conserved hypothetical protein | IPR003042: Aromatic-ring hydroxylase-like, IPR006076: FAD dependent oxidoreductase |
| MGG09319.6 | 0.47 | conserved hypothetical protein | IPR013090: Phospholipase A2, active site. |
| MGG04449.6 | 0.47 | branched-chain alpha-keto acid lipoamide acyltransferase | IPR000089: Biotin/lipoyl attachment, IPR015761 : Lipoamide Acyltransferase |
| MGG00015.6 | 0.47 | catechol O-methyltransferase | IPR002935: O-methyltransferase, family 3 |
| MGG15421.6 | 0.47 | monooxygenase |  |
| MGG04009.6 | 0.47 | bZIP transcription factor | IPR004827: Basic-leucine zipper (bZIP) transcription factor |
| MGG04329.6 | 0.47 | conserved hypothetical protein | IPR011701: Major facilitator superfamily MFS-1, PR016196: Major facilitator superfamily, general substrate transporter |
| MGG04825.6 | 0.47 | endopolyphosphatase | IPR004843: Metallophosphoesterase, IPR012358: Endopolyphosphatase, Ppn1p-related |
| MGG10371.6 | 0.46 | glutaredoxin-C4 precursor | IPR002109: Glutaredoxin, IPR012335: Thioredoxin fold |
| MGG05008.6 | 0.46 | aldehyde dehydrogenase | IPR015590: Aldehyde dehydrogenase |
| MGG00663.6 | 0.46 | phytanoyl-CoA dioxygenase family protein | IPR008775 : Phytanoyl-CoA dioxygenase |
| MGG09017.6 | 0.46 | rieske domain-containing protein | IPR002048: Calcium-binding EF-hand, IPR005806: Rieske [2Fe-2S] region |
| MGG07698.6 | 0.46 | hypothetical protein | No defined Interpro term |
| MGG07643.6 | 0.46 | conserved hypothetical protein | No defined Interpro term |
| MGG01900.6 | 0.46 | hypothetical protein | No defined Interpro term |
| MGG04915.6 | 0.46 | hypothetical protein | No defined Interpro term |
| MGG10372.6 | 0.46 | hypothetical protein | No defined Interpro term |
| MGG14654.6 | 0.46 | hypothetical protein | No defined Interpro term |
| MGG00312.6 | 0.46 | glyoxylate reductase | IPR006140: D-isomer specific 2-hydroxyacid dehydrogenase, NAD-binding |
| MGG02825.6 | 0.45 | short-chain dehydrogenase | IPR002198: Short-chain dehydrogenase/reductase SDR, IPR016040 NAD(P)-binding |
| MGG12942.6 | 0.45 | hypothetical protein | No defined Interpro term |
| MGG05141.6 | 0.45 | conserved hypothetical protein | No defined Interpro term |
| MGG03451.6 | 0.45 | C2H2 zinc finger | IPR007087: Zinc finger, C2H2-type |
| MGG08686.6 | 0.45 | hypothetical protein | No defined Interpro term |
| MGG04160.6 | 0.45 | vacuolar membrane-associated protein IML1 | IPR000591: Pleckstrin/G-protein, interacting region, IPR011991: Winged helix repressor DNA-binding |
| MGG06161.6 | 0.45 | conserved hypothetical protein | IPR013216: Methyltransferase type 11 |
| MGG10634.6 | 0.45 | conserved hypothetical protein | IPR003370: Chromate transporter region |
| MGG01429.6 | 0.45 | conserved hypothetical protein | IPR011513: Nse1 non-SMC component of SMC5-6 complex, IPR013083: Zinc finger, RING/FYVE/PHD-type, IPR014857: Zinc finger, RING-like |
| MGG04334.6 | 0.45 | FAD binding domain-containing protein | IPR002938: Monooxygenase, IPR005829: Sugar transporter, conserved site |
| MGG01679.6 | 0.45 | ThiJ/PfpI family protein | IPR002818: ThiJ/PfpI |
| MGG13334.6 | 0.45 | general amino acid permease AGP2 | IPR002293: Amino acid/polyamine transporter I, IPR004841: Amino acid permease-associated region |
| MGG00527.6 | 0.45 | hypothetical protein | No defined Interpro term |
| MGG04157.6 | 0.45 | hypothetical protein | IPR010730: Heterokaryon incompatibility |
| MGG03293.6 | 0.45 | conserved hypothetical protein | IPR005493: Ribonuclease E inhibitor RraA/Dimethylmenaquinone methyltransferase |
| MGG13020.6 | 0.45 | glucose-repressible alcohol dehydrogenase transcriptional effector | IPR001611: Leucine-rich repeat, IPR005135: Endonuclease/exonuclease/phosphatase |
| MGG03304.6 | 0.45 | hypothetical protein | No defined Interpro term |
| MGG02374.6 | 0.45 | hypothetical protein | IPR001810: Cyclin-like F-box |
| MGG01007.6 | 0.45 | conserved hypothetical protein | No defined Interpro term |
| MGG05910.6 | 0.45 | deoxyribonuclease tatD | IPR001130: Deoxyribonuclease, TatD-related, IPR012278 : Deoxyribonuclease, TatD Mg-dependent, IPR015992 : Deoxyribonuclease, TatD |
| MGG07316.6 | 0.45 | FACT complex subunit spt-16 | IPR000994: Peptidase M24, structural domain, IPR013719 : Region of unknown function DUF1747, eukaryote, IPR013953 : FACT complex subunit Spt16p/Cdc68p |
| MGG07504.6 | 0.44 | NADH-ubiquinone oxidoreductase 21.3 kDa subunit | IPR016813: NADH dehydrogenase [ubiquinone] (complex I), 21 kDa subunit, fungi |
| MGG06923.6 | 0.44 | myosin-2 | IPR000048: IQ calmodulin-binding region, IPR001609 : Myosin head, motor region, IPR013805: GrpE nucleotide exchange factor, coiled-coil |
| MGG09784.6 | 0.44 | hypothetical protein | No defined Interpro term |
| MGG10150.6 | 0.44 | pH-response transcription factor pacC/RIM101 | IPR007087 : Zinc finger, C2H2-type |
| MGG10235.6 | 0.44 | hypothetical protein | No defined Interpro term |
| MGG07703.6 | 0.44 | hypothetical protein | No defined Interpro term |
| MGG09786.6 | 0.44 | conserved hypothetical protein | No defined Interpro term |
| MGG01855.6 | 0.44 | hypothetical protein | No defined Interpro term |
| MGG05071.6 | 0.44 | conserved hypothetical protein | IPR011046: WD40 repeat-like |
| MGG04084.6 | 0.44 | platelet-activating factor acetylhydrolase precursor | IPR005065: Platelet-activating factor acetylhydrolase, plasma/intracellular isoform II |
| MGG00820.6 | 0.44 | conserved hypothetical protein | IPR010730: Heterokaryon incompatibility |
| MGG09377.6 | 0.44 | hypothetical protein | No defined Interpro term |
| MGG04377.6 | 0.44 | rho-type GTPase-activating protein 1 | IPR000198: RhoGAP, IPR001781: Zinc finger, LIM-type, IPR008936: Rho GTPase activation protein |
| MGG02919.6 | 0.44 | conserved hypothetical protein | No defined Interpro term |
| MGG05128.6 | 0.43 | dicarboxylic amino acid permease | IPR002293: Amino acid/polyamine transporter I, IPR004840: Amino acid permease, conserved site |
| MGG01292.6 | 0.43 | nucleolar protein NOP2 | IPR001678: Bacterial Fmu (Sun)/eukaryotic nucleolar NOL1/Nop2p |
| MGG02744.6 | 0.43 | hypothetical protein | IPR000408: Regulator of chromosome condensation |
| MGG07272.6 | 0.43 | conserved hypothetical protein | IPR007235: Glycosyl transferase, family 28, C-terminal |
| MGG13767.6 | 0.43 | lovastatin nonaketide synthase | IPR000794: Beta-ketoacyl synthase, PR001227: Acyl transferase region |
| MGG02475.6 | 0.43 | vitamin H transporter 1 /biotin symporter vht1 | IPR011701: Major facilitator superfamily MFS-1, IPR016196: Major facilitator superfamily, general substrate transporter |
| MGG08583.6 | 0.43 | beta-glucosidase 1 precursor | IPR001764: Glycoside hydrolase, family 3, N-terminal, IPR017853: Glycoside hydrolase, catalytic core |
| MGG09935.6 | 0.43 | serine/threonine-protein phosphatase 2A activator 2 | IPR00432 : Phosphotyrosyl phosphatase activator, PTPA |
| MGG04435.6 | 0.43 | bifunctional purine biosynthesis protein ADE17 | IPR002695: AICARFT/IMPCHase bienzyme, IPR011607 : MGS-like |
| MGG04364.6 | 0.43 | conserved hypothetical protein | No defined Interpro term |
| MGG08012.6 | 0.43 | UDP-glucose 4-epimerase | IPR001509: NAD-dependent epimerase/dehydratase, IPR005886: UDP-glucose 4-epimerase, IPR016040: NAD(P)-binding |
| MGG15451.6 | 0.43 | conserved hypothetical protein | No defined Interpro term |
| MGG04333.6 | 0.43 | hypothetical protein | No defined Interpro term |
| MGG01705.6 | 0.42 | sequence orphan | No defined Interpro term |
| MGG05722.6 | 0.42 | conserved hypothetical protein | IPR002345: Lipocalin, IPR005828: General substrate transporter, IPR016196: Major facilitator superfamily, general substrate transporter |
| MGG07609.6 | 0.42 | hypothetical protein | No defined Interpro term |
| MGG01014.6 | 0.42 | C-1-tetrahydrofolate synthase | IPR000559: Formate-tetrahydrofolate ligase, FTHFS ,IPR000672: Tetrahydrofolate dehydrogenase/cyclohydrolase, IPR016040: NAD(P)-binding |
| MGG11346.6 | 0.42 | hypothetical protein | IPR015880: Zinc finger, C2H2-like |
| MGG15037.6 | 0.42 | hypothetical protein | No defined Interpro term |
| MGG06216.6 | 0.42 | hypothetical protein | No defined Interpro term |
| MGG15073.6 | 0.42 | conserved hypothetical protein | No defined Interpro term |
| MGG14805.6 | 0.42 | DUF907 domain-containing protein | IPR010308: Protein of unknown function DUF907, fungi |
| MGG13895.6 | 0.42 | fructose-bisphosphate aldolase | IPR000771: Ketose-bisphosphate aldolase, class-II, IPR013785: Aldolase-type TIM barrel |
| MGG00692.6 | 0.42 | cell pattern formation-associated protein stuA | IPR003163: APSES-type HTH DNA-binding domain |
| MGG09125.6 | 0.42 | osmosensor protein | IPR001452: Src homology-3 domain, IPR002345: Lipocalin |
| MGG03147.6 | 0.42 | glycerol-3-phosphate dehydrogenase | IPR000447: FAD-dependent glycerol-3-phosphate dehydrogenase, PR006076: FAD dependent oxidoreductase |
| MGG04341.6 | 0.42 | hypothetical protein | No defined Interpro term |
| MGG07630.6 | 0.42 | hypothetical protein | No defined Interpro term |
| MGG00220.6 | 0.41 | NADP-dependent alcohol dehydrogenase 6 | IPR002085: Alcohol dehydrogenase superfamily, zinc-containing, IPR011032: GroES-like |
| MGG04934.6 | 0.41 | peptide transporter PTR2-A | IPR000109: TGF-beta receptor, type I/II extracellular region, IPR016196: Major facilitator superfamily, general substrate transporter |
| MGG01085.6 | 0.41 | ThiJ/PfpI family protein | IPR002818: ThiJ/PfpI |
| MGG04212.6 | 0.41 | L-ornithine 5-monooxygenase | No defined Interpro term |
| MGG06361.6 | 0.41 | dynamin-A | IPR000375: Dynamin central region, IPR001401: Dynamin, GTPase region, IPR005829: Sugar transporter |
| MGG02091.6 | 0.41 | hypothetical protein | No defined Interpro term |
| MGG07376.6 | 0.41 | hypothetical protein | No defined Interpro term |
| MGG03161.6 | 0.41 | conserved hypothetical protein | IPR014751: DNA double-strand break repair and VJ recombination XRCC4, C-terminal |
| MGG09499.6 | 0.41 | ras-2 | IPR001806: Ras GTPase, IPR003577: Ras small GTPase, Ras type, IPR005225: Small GTP-binding protein |
| MGG07139.6 | 0.41 | hypothetical protein | No defined Interpro term |
| MGG05234.6 | 0.41 | conserved hypothetical protein | IPR016084: Haem oxygenase-like, multi-helical |
| MGG07312.6 | 0.41 | osmosensing histidine protein kinase SLN1 | IPR001789: Signal transduction response regulator, receiver region, IPR003594: ATP-binding region, ATPase-like, IPR003661: Signal transduction histidine kinase, subgroup 1, dimerisation and phosphoacceptor region |
| MGG00494.6 | 0.41 | transcriptional regulatory protein pro-1 | IPR001138: Fungal transcriptional regulatory protein, N-terminal |
| MGG06610.6 | 0.41 | lipase | No defined Interpro term |
| MGG09766.6 | 0.41 | phospho-2-dehydro-3-deoxyheptonate aldolase | IPR006218: DAHP synthetase I/KDSA, IPR013785: Aldolase-type TIM barrel |
| MGG00521.6 | 0.40 | conserved hypothetical protein | No defined Interpro term |
| MGG00041.6 | 0.40 | conserved hypothetical protein | IPR002935: O-methyltransferase, family 3 |
| MGG00134.6 | 0.40 | von Willebrand and RING finger domain-containing protein | IPR001841: Zinc finger, RING-type, IPR002035: von Willebrand factor, type A |
| MGG05760.6 | 0.40 | conserved hypothetical protein | IPR015362: Exon junction complex, Pym |
| MGG02937.6 | 0.40 | conserved hypothetical protein | ,IPR001214: SET, IPR003616: Post-SET zinc-binding region |
| MGG08200.6 | 0.40 | conserved hypothetical protein | IPR002016: Haem peroxidase, plant/fungal/bacterial, IPR002889: Carbohydrate-binding WSC, IPR010255: Haem peroxidase |
| MGG05201.6 | 0.40 | guanine nucleotide-binding protein subunit beta | IPR001632: G-protein, beta subunit, IPR001680: WD40 repeat, |
| MGG08832.6 | 0.40 | C-5 sterol desaturase | IPR006088 |
| MGG06761.6 | 0.40 | monooxygenase | IPR002938: Monooxygenase, FAD-binding |
| MGG08187.6 | 0.40 | thiol-specific monooxygenase | IPR000759: Adrenodoxin reductase, IPR013027: FAD-dependent pyridine nucleotide-disulphide oxidoreductase |
| MGG10820.6 | 0.40 | conserved hypothetical protein | No defined Interpro term |
| MGG06971.6 | 0.40 | flocculation suppression protein | IPR000232: Heat shock factor (HSF)-type, DNA-binding, IPR011991: Winged helix repressor DNA-binding |
| MGG04134.6 | 0.40 | aminotransferase | IPR004839: Aminotransferase, class I and II, IPR015421: Pyridoxal phosphate-dependent transferase, major region, subdomain 1 |
| MGG04677.6 | 0.40 | conserved hypothetical protein | IPR003006: Immunoglobulin/major histocompatibility complex, conserved site |
| MGG01467.6 | 0.40 | transmembrane protein 34 | IPR005178: Protein of unknown function DUF300 |
| MGG05768.6 | 0.39 | hypothetical protein | No defined Interpro term |
| MGG06498.6 | 0.39 | phosphorylase family protein | IPR001369: Purine phosphorylase, family 2, IPR010044: Methylthioadenosine phosphorylase |
| MGG09073.6 | 0.39 | minor extracellular protease vpr | IPR000209: Peptidase S8 and S53, subtilisin, kexin, sedolisin, IPR008162: Inorganic pyrophosphatase, IPR015500: Peptidase S8, subtilisin-related |
| MGG02865.6 | 0.39 | bZIP transcription factor | IPR004827: Basic-leucine zipper (bZIP) transcription factor, IPR011700: Basic leucine zipper |
| MGG09329.6 | 0.39 | hypothetical protein | No defined Interpro term |
| MGG00564.6 | 0.39 | hypothetical protein | No defined Interpro term |
| MGG05019.6 | 0.39 | conserved hypothetical protein | IPR001163: Like-Sm ribonucleoprotein, core |
| MGG09215.6 | 0.39 | conserved hypothetical protein | No defined Interpro term |
| MGG05183.6 | 0.39 | cript family protein | No defined Interpro term |
| MGG08731.6 | 0.39 | conserved hypothetical protein | IPR003864 : Protein of unknown function DUF221 |
| MGG08832.6 | 0.39 | C-5 sterol desaturase |  |
| MGG00986.6 | 0.39 | 2-dehydropantoate 2-reductase | IPR003710: Ketopantoate reductase ApbA/PanE,IPR008927 : 6-phosphogluconate dehydrogenase, C-terminal-like |
| MGG08214.6 | 0.38 | hypothetical protein | IPR000172: Glucose-methanol-choline oxidoreductase, N-terminal |
| MGG00297.6 | 0.38 | conserved hypothetical protein | No defined Interpro term |
| MGG05103.6 | 0.38 | conserved hypothetical protein | No defined Interpro term |
| MGG03920.6 | 0.38 | inositolphosphorylceramide-B C-26 hydroxylase | IPR006694: Fatty acid hydroxylase |
| MGG14912.6 | 0.38 | DNA replication licensing factor mcm6 | IPR001208: DNA-dependent ATPase MCM, IPR012340: Nucleic acid-binding, OB-fold |
| MGG10684.6 | 0.38 | conserved hypothetical protein | No defined Interpro term |
| MGG10591.6 | 0.38 | endo-beta-1,3-glucanase | IPR017853: Glycoside hydrolase, catalytic core |
| MGG07744.6 | 0.38 | cwfJ domain-containing protein | IPR006767: Protein similar to CwfJ, C-terminal 2 |
| MGG14676.6 | 0.38 | conserved hypothetical protein | No defined Interpro term |
| MGG02269.6 | 0.38 | conserved hypothetical protein | IPR002654: Glycosyl transferase, family 25 |
| MGG03371.6 | 0.38 | conserved hypothetical protein | IPR003892: Ubiquitin system component Cue, IPR008162: Inorganic pyrophosphatase |
| MGG07980.6 | 0.38 | metabolite transporter | IPR005829: Sugar transporter, conserved site, IPR011701: Major facilitator superfamily MFS-1 |
| MGG10510.6 | 0.38 | ribonuclease T2 | IPR001568: Ribonuclease T2 |
| MGG00409.6 | 0.37 | conserved hypothetical protein | No defined Interpro term |
| MGG00675.6 | 0.37 | hypothetical protein | No defined Interpro term |
| MGG09728.6 | 0.37 | lactose permease | IPR003663: Sugar/inositol transporter, IPR005828 General substrate transporter, IPR016196: Major facilitator superfamily, general substrate transporter |
| MGG08618.6 | 0.37 | conserved hypothetical protein |  |
| MGG02715.6 | 0.37 | hypothetical protein | No defined Interpro term |
| MGG03121.6 | 0.37 | conserved hypothetical protein | No defined Interpro term |
| MGG04159.6 | 0.37 | vacuolar calcium ion transporter | IPR004713: Calcium/proton exchanger, R004837: Sodium/calcium exchanger membrane region |
| MGG00545.6 | 0.37 | hypothetical protein | No defined Interpro term |
| MGG01655.6 | 0.37 | copper radical oxidase | IPR002889: Carbohydrate-binding WSC, PR00988: Glyoxal oxidase, N-terminal, IPR011043: Galactose oxidase/kelch, beta-propeller |
| MGG05690.6 | 0.37 | conserved hypothetical protein | IPR002889: Carbohydrate-binding WSC, IPR013994: Carbohydrate-binding WSC, subgroup |
| MGG13895.6 | 0.37 | fructose-bisphosphate aldolase |  |
| MGG01849.6 | 0.37 | conserved hypothetical protein | No defined Interpro term |
| MGG08898.6 | 0.37 | conserved hypothetical protein | IPR000504: RNA recognition motif, RNP-1, IPR012677: Nucleotide-binding, alpha-beta plait, IPR015903: Ribonucleoprotein, BRUNO-like |
| MGG07295.6 | 0.37 | conserved hypothetical protein | IPR015609: Molecular chaperone, heat shock protein, Hsp40, DnaJ |
| MGG09828.6 | 0.36 | vacuolar conductance protein | No defined Interpro term |
| MGG03817.6 | 0.36 | metalloprotease 1 | IPR008754: Peptidase M43B, pregnancy-associated plasma-A |
| MGG03535.6 | 0.36 | conserved hypothetical protein | No defined Interpro term |
| MGG00535.6 | 0.36 | conserved hypothetical protein | IPR000719: Protein kinase, core |
| MGG12130.6 | 0.36 | serine/threonine protein phosphatase 2A | IPR002554: Protein phosphatase 2A, regulatory B subunit, B56 |
| MGG01470.6 | 0.36 | conserved hypothetical protein | No defined Interpro term |
| MGG15036.6 | 0.36 | conserved hypothetical protein | No defined Interpro term |
| MGG03664.6 | 0.36 | palmitoyltransferase ERF2 | IPR001594: Zinc finger, DHHC-type |
| MGG09709.6 | 0.36 | endoglucanase II | IPR005103: Glycoside hydrolase, family 61 |
| MGG08618.6 | 0.36 | conserved hypothetical protein | IPR001138: Fungal transcriptional regulatory protein, N-terminal, IPR007219 : Fungal specific transcription factor |
| MGG03837.6 | 0.36 | conserved hypothetical protein | IPR013941: Sporulation protein Zds1, C-terminal |
| MGG06968.6 | 0.36 | WD repeat-containing protein | IPR001680: WD40 repeat |
| MGG01151.6 | 0.36 | integral membrane protein | No defined Interpro term |
| MGG02807.6 | 0.36 | RNAse P Rpr2/Rpp21 subunit domain-containing protein | IPR007175: RNAse P, Rpr2/Rpp21 subunit |
| MGG13218.6 | 0.36 | pyruvate formate lyase activating enzyme | IPR007197: Radical SAM, IPR016431: Pyruvate-formate lyase-activating enzyme, predicted |
| MGG03914.6 | 0.36 | hypothetical protein | No defined Interpro term |
| MGG06533.6 | 0.36 | hypothetical protein | No defined Interpro term |
| MGG04893.6 | 0.36 | allantoate permease | IPR011701: Major facilitator superfamily MFS-1, IPR016196 : Major facilitator superfamily, general substrate transporter |
| MGG02916.6 | 0.36 | hypothetical protein | No defined Interpro term |
| MGG05735.6 | 0.35 | lactate 2-monooxygenase | IPR000262: FMN-dependent dehydrogenase |
| MGG02001.6 | 0.35 | conserved hypothetical protein | No defined Interpro term |
| MGG03133.6 | 0.35 | conserved hypothetical protein | IPR013087: Zinc finger, C2H2-type/integrase, DNA-binding |
| MGG00718.6 | 0.35 | hypothetical protein | IPR008914: Phosphatidylethanolamine-binding protein PEBP |
| MGG00373.6 | 0.35 | conserved hypothetical protein | IPR007087: Zinc finger, C2H2-type |
| MGG08447.6 | 0.35 | conserved hypothetical protein | IPR016040: NAD(P)-binding |
| MGG00832.6 | 0.35 | cytochrome P450 | IPR001128: Cytochrome P450 |
| MGG01204.6 | 0.35 | MADS-box MEF2 type transcription factor | IPR002100: Transcription factor, MADS-box |
| MGG13924.6 | 0.35 | serine palmitoyltransferase 2 | IPR001917: Aminotransferase, class-II, pyridoxal-phosphate binding site, IPR001951: Histone H4 |
| MGG00276.6 | 0.35 | FAD binding domain protein | IPR006094: FAD linked oxidase N-terminal, IPR016166: FAD-binding, type 2 |
| MGG03337.6 | 0.35 | endoprotease | IPR008758: Peptidase S28 |
| MGG12091.6 | 0.35 | hypothetical protein | No defined Interpro term |
| MGG15138.6 | 0.35 | conserved hypothetical protein | No defined Interpro term |
| MGG15092.6 | 0.35 | hypothetical protein | No defined Interpro term |
| MGG06157.6 | 0.35 | abhydrolase domain-containing protein 4 | IPR000073: Alpha/beta hydrolase fold-1 |
| MGG13697.6 | 0.34 | conserved hypothetical protein | IPR011009: Protein kinase-like |
| MGG00903.6 | 0.34 | frequency clock protein | No defined Interpro term |
| MGG03123.6 | 0.34 | multidrug and toxin extrusion protein 1 | IPR002528: Multi antimicrobial extrusion protein MatE |
| MGG09421.6 | 0.34 | hypothetical protein | No defined Interpro term |
| MGG04110.6 | 0.34 | queuine tRNA-ribosyltransferase | ,IPR002616: Queuine/other tRNA-ribosyltransferase |
| MGG01519.6 | 0.34 | conserved hypothetical protein | IPR000504: RNA recognition motif, RNP-1, IPR002344: Lupus La protein |
| MGG06634.6 | 0.34 | hypothetical protein | IPR001841 : Zinc finger, RING-type |
| MGG08783.6 | 0.34 | conserved hypothetical protein | IPR015590: Aldehyde dehydrogenase |
| MGG10276.6 | 0.34 | conserved hypothetical protein | IPR00708 : Zinc finger, C2H2-type |
| MGG07303.6 | 0.34 | hypothetical protein | No defined Interpro term |
| MGG00245.6 | 0.34 | conserved hypothetical protein | No defined Interpro term |
| MGG04738.6 | 0.34 | bacilysin biosynthesis oxidoreductase bacC | IPR002198: Short-chain dehydrogenase/reductase SDR, IPR002347: Glucose/ribitol dehydrogenase |
| MGG01622.6 | 0.34 | uroporphyrinogen decarboxylase | IPR006361 : Uroporphyrinogen decarboxylase HemE |
| MGG10514.6 | 0.34 | conserved hypothetical protein | No defined Interpro term |
| MGG02084.6 | 0.34 | glyoxylate reductase | IPR016040 : NAD(P)-binding |
| MGG08887.6 | 0.34 | conserved hypothetical protein | IPR001849 : Pleckstrin homology |
| MGG07883.6 | 0.34 | sorbose reductase sou1 | IPR002198: Short-chain dehydrogenase/reductase SDR, IPR002347 : Glucose/ribitol dehydrogenase, IPR016040 : NAD(P)-binding,IPR016040 : NAD(P)-binding |
| MGG02223.6 | 0.34 | conserved hypothetical protein | No defined Interpro term |
| MGG04640.6 | 0.34 | sulfate permease 2 | IPR001902: Sulphate anion transporter |
| MGG01728.6 | 0.33 | methylenetetrahydrofolate reductase 1 | IPR003171: Methylenetetrahydrofolate reductase |
| MGG02000.6 | 0.33 | conserved hypothetical protein | IPR003480: Transferase |
| MGG07140.6 | 0.33 | conserved hypothetical protein | IPR007087: Zinc finger, C2H2-type I |
| MGG03340.6 | 0.33 | conserved hypothetical protein | No defined Interpro term |
| MGG09752.6 | 0.33 | conserved hypothetical protein | No defined Interpro term |
| MGG07704.6 | 0.33 | carboxypeptidase A | IPR000834: Peptidase M14, carboxypeptidase A |
| MGG09860.6 | 0.33 | hypothetical protein | No defined Interpro term |
| MGG00298.6 | 0.33 | conserved hypothetical protein | IPR001810: Cyclin-like F-box |
| MGG09648.6 | 0.32 | conserved hypothetical protein | No defined Interpro term |
| MGG02543.6 | 0.32 | conserved hypothetical protein | IPR001087: Lipase, GDSL, IPR013830 : Esterase, SGNH hydrolase-type |
| MGG13202.6 | 0.32 | conserved hypothetical protein | No defined Interpro term |
| MGG09210.6 | 0.32 | endosome protein | IPR001870: B302 (SPRY)-like ,IPR003877 : SPla/RYanodine receptor SPRY |
| MGG10852.6 | 0.32 | methionine synthase | IPR002629: Methionine synthase, vitamin-B12 independent |
| MGG10956.6 | 0.32 | hypothetical protein | No defined Interpro term |
| MGG04847.6 | 0.32 | peptidase M14 | IPR000834: Peptidase M14, carboxypeptidase A |
| MGG08046.6 | 0.32 | bilirubin oxidase | IPR002355: Multicopper oxidase, copper-binding site |
| MGG07597.6 | 0.31 | conserved hypothetical protein | IPR011701: Major facilitator superfamily MFS-1 |
| MGG03360.6 | 0.31 | carboxylic acid transport protein | IPR011701: Major facilitator superfamily MFS-1 |
| MGG10651.6 | 0.31 | 50S ribosomal protein L17 | IPR000456: Ribosomal protein L17 |
| MGG09458.6 | 0.31 | GTP cyclohydrolase I | IPR001474: GTP cyclohydrolase I |
| MGG10969.6 | 0.31 | kynureninase | IPR010111: Kynureninase, IPR015421: Pyridoxal phosphate-dependent transferase, major region, subdomain 1 |
| MGG13202.6 | 0.31 | conserved hypothetical protein |  |
| MGG13629.6 | 0.31 | fungal specific transcription factor domain protein | No defined Interpro term |
| MGG10318.6 | 0.31 | conserved hypothetical protein | No defined Interpro term |
| MGG03761.6 | 0.31 | conserved hypothetical protein | IPR000194: ATPase, F1/V1/A1 complex, alpha/beta subunit, nucleotide-binding, IPR002889 : Carbohydrate-binding WSC |
| MGG03324.6 | 0.31 | conserved hypothetical protein | IPR005123: 2OG-Fe(II) oxygenase |
| MGG05901.6 | 0.31 | conserved hypothetical protein | No defined Interpro term |
| MGG08047.6 | 0.30 | glycerophosphoryl diester phosphodiesterase family protein | IPR004129: Glycerophosphoryl diester phosphodiesterase, IPR017946: PLC-like phosphodiesterase, TIM beta/alpha-barrel domain |
| MGG13192.6 | 0.30 | conserved hypothetical protein | IPR001958: Tetracycline resistance protein, TetA, IPR011701 : Major facilitator superfamily MFS-1 |
| MGG08105.6 | 0.30 | GTPase activating protein | IPR001936: Ras GTPase-activating protein ,IPR008973: C2 calcium/lipid-binding region, CaLB |
| MGG00969.6 | 0.30 | asparagine synthetase 1 | IPR000583: Glutamine amidotransferase, class-II, IPR001962 : Asparagine synthase, IPR006426 : Asparagine synthase, glutamine-hydrolyzing |
| MGG05769.6 | 0.30 | hypothetical protein | No defined Interpro term |
| MGG04979.6 | 0.30 | GPI mannosyltransferase 4 | IPR005599: Alg9-like mannosyltransferase |
| MGG00505.6 | 0.30 | septation protein SUN4 | IPR005556: SUN |
| MGG14657.6 | 0.30 | conserved hypothetical protein | No defined Interpro term |
| MGG04305.6 | 0.30 | cell wall glycosyl hydrolase YteR | IPR008928: Six-hairpin glycosidase-like, IPR010905 : Glycosyl hydrolase, family 88 |
| MGG02851.6 | 0.30 | conserved hypothetical protein | No defined Interpro term |
| MGG07553.6 | 0.30 | CFEM domain-containing protein | No defined Interpro term |
| MGG08799.6 | 0.30 | hypothetical protein | No defined Interpro term |
| MGG08845.6 | 0.30 | conserved hypothetical protein | No defined Interpro term |
| MGG10237.6 | 0.30 | hypothetical protein | No defined Interpro term |
| MGG02626.6 | 0.30 | septin | IPR000038: Cell division/GTP binding protein |
| MGG06372.6 | 0.30 | F-box/WD repeat-containing protein 7 | IPR001680: WD40 repeat, IPR001810 : Cyclin-like F-box |
| MGG10533.6 | 0.30 | agmatinase | IPR005924: Arginase |
| MGG07243.6 | 0.30 | conserved hypothetical protein | IPR001164: Arf GTPase activating protein, IPR015940: Ubiquitin-associated/translation elongation factor EF1B, N-terminal, eukaryote |
| MGG03843.6 | 0.30 | conserved hypothetical protein | IPR011701: Major facilitator superfamily MFS-1, IPR016196: Major facilitator superfamily, general substrate transporter |
| MGG10300.6 | 0.29 | conserved hypothetical protein | No defined Interpro term |
| MGG00110.6 | 0.29 | conserved hypothetical protein | IPR013831: Esterase, SGNH hydrolase-type, subgroup |
| MGG07228.6 | 0.29 | conserved hypothetical protein | IPR004813: Oligopeptide transporter OPT superfamily |
| MGG00631.6 | 0.29 | conserved hypothetical protein | No defined Interpro term |
| MGG07780.6 | 0.29 | 3-dehydroshikimate dehydratase | IPR012307: Xylose isomerase-type TIM barrel, IPR013022: Xylose isomerase-like, TIM barrel |
| MGG14157.6 | 0.29 | conserved hypothetical protein | IPR002227: Tyrosinase, IPR008922: Di-copper centre-containing |
| MGG03671.6 | 0.29 | hypothetical protein | No defined Interpro term |
| MGG03668.6 | 0.29 | importin subunit beta-1 | IPR000357: HEAT, IPR001494 : Importin-beta, N-terminal, IPR011989: Armadillo-like helical |
| MGG06653.6 | 0.29 | conserved hypothetical protein | IPR008972: Cupredoxin |
| MGG00282.6 | 0.29 | conserved hypothetical protein | IPR000209: Peptidase S8 and S53, subtilisin, kexin, sedolisin |
| MGG10574.6 | 0.28 | GNAT family N-acetyltransferase | IPR000182: GCN5-related N-acetyltransferase, IPR016181: Acyl-CoA N-acyltransferase |
| MGG07099.6 | 0.28 | ATP-dependent RNA helicase mss116, mitochondrial precursor | IPR000629: RNA helicase, ATP-dependent, DEAD-box, conserved site, IPR01154: DNA/RNA helicase, DEAD/DEAH box type, N-terminal |
| MGG11610.6 | 0.28 | hypothetical protein | No defined Interpro term |
| MGG05413.6 | 0.28 | MFS transporter | IPR011701: Major facilitator superfamily MFS-1 |
| MGG00992.6 | 0.28 | conserved hypothetical protein | No defined Interpro term |
| MGG04071.6 | 0.28 | hypothetical protein | No defined Interpro term |
| MGG10297.6 | 0.28 | conserved hypothetical protein | IPR012602: PyrBI operon leader peptide |
| MGG10287.6 | 0.28 | metabolite transporter | IPR005828: General substrate transporter, IPR005829: Sugar transporter, conserved site |
| MGG07619.6 | 0.28 | taurine catabolism dioxygenase TauD | IPR003819 : Taurine catabolism dioxygenase TauD/TfdA |
| MGG07327.6 | 0.28 | asparagine-rich protein |  |
| MGG04386.6 | 0.28 | urea amidolyase | IPR000089: Biotin/lipoyl attachment, IPR000120: Amidase signature enzyme, IPR003778: Allophanate hydrolase subunit 2, IPR005479: Carbamoyl phosphate synthetase, large subunit, ATP-binding, IPR013817: Pre-ATP-grasp fold IPR014085: Allophanate hydrolase |
| MGG08214.6 | 0.28 | hypothetical protein |  |
| MGG15455.6 | 0.27 | hypothetical protein | No defined Interpro term |
| MGG10593.6 | 0.27 | hypothetical protein | No defined Interpro term |
| MGG04951.6 | 0.27 | hypothetical protein | IPR001138: Fungal transcriptional regulatory protein, N-terminal |
| MGG09693.6 | 0.27 | conserved hypothetical protein | No defined Interpro term |
| MGG06183.6 | 0.27 | hypothetical protein | IPR000535: Major sperm protein, IPR008962 : PapD-like, IPR016763: Vesicle-associated membrane protein |
| MGG11702.6 | 0.27 | lanosterol synthase | IPR001330: Prenyltransferase/squalene oxidase, IPR002365 : Terpene synthase, conserved site, IPR008930 : Terpenoid cylases/protein prenyltransferase alpha-alpha toroid |
| MGG00593.6 | 0.27 | liver carboxylesterase | IPR002018: Carboxylesterase, type B |
| MGG04545.6 | 0.27 | cytochrome c peroxidase | IPR002016: Haem peroxidase, plant/fungal/bacterial, IPR002207 : Plant ascorbate peroxidase, IPR010255 : Haem peroxidase |
| MGG00849.6 | 0.27 | conserved hypothetical protein | IPR000253 : Forkhead-associated, IPR008984 : SMAD/FHA domain |
| MGG09956.6 | 0.27 | PRO41 protein | No defined Interpro term |
| MGG10995.6 | 0.27 | conserved hypothetical protein | No defined Interpro term |
| MGG06135.6 | 0.27 | GTP-binding protein SAS1 | IPR001806: Ras GTPase, IPR003579: Ras small GTPase, Rab type, IPR005225 : Small GTP-binding protein, IPR013753 : Ras |
| MGG06218.6 | 0.27 | RNA-binding post-transcriptional regulator cip2 | IPR000504: RNA recognition motif, RNP-1, IPR001374: Single-stranded nucleic acid binding R3H, IPR012677: Nucleotide-binding, alpha-beta plait |
| MGG15306.6 | 0.27 | conserved hypothetical protein | IPR002654 : Glycosyl transferase, family 25 |
| MGG04728.6 | 0.27 | hypothetical protein | No defined Interpro term |
| MGG01888.6 | 0.27 | WD repeat-containing protein | IPR001680 : WD40 repeat |
| MGG10116.6 | 0.27 | conserved hypothetical protein | IPR008696 : NAF1 |
| MGG06062.6 | 0.26 | nitrate reductase | IPR000572: Oxidoreductase, molybdopterin binding, IPR001199: Cytochrome b5, IPR001709: Flavoprotein pyridine nucleotide cytochrome reductase, IPR001834: NADH:cytochrome b5 reductase (CBR), IPR005066: Moybdenum cofactor oxidoreductase, dimerization I, IPR012137: Nitrate reductase NADH dependant, IPR014756: Immunoglobulin E-set, IPR017927: Ferredoxin reductase-type FAD-binding domain, IPR017938: Riboflavin synthase-like beta-barrel |
| MGG15337.6 | 0.26 | zygote-specific protein | No defined Interpro term |
| MGG05788.6 | 0.26 | Poly(3-hydroxybutyrate) depolymerase | No defined Interpro term |
| MGG11852.6 | 0.26 | hypothetical protein | No defined Interpro term |
| MGG01823.6 | 0.26 | conserved hypothetical protein | No defined Interpro term |
| MGG09307.6 | 0.26 | hexose transporter protein | IPR003663: Sugar/inositol transporter, IPR005828: General substrate transporter |
| MGG13773.6 | 0.26 | indoleamine 2,3-dioxygenase family protein | IPR000898: Indoleamine 2,3-dioxygenase |
| MGG11454.6 | 0.26 | vacuolar calcium ion transporter | IPR004713: Calcium/proton exchanger, IPR004837: Sodium/calcium exchanger membrane region |
| MGG05946.6 | 0.26 | high-affinity glucose transporter | IPR003663: Sugar/inositol transporter, IPR005828: General substrate transporter, IPR016196: Major facilitator superfamily, general substrate transporter |
| MGG08980.6 | 0.26 | stress-induced-phosphoprotein 1 | IPR001440 : Tetratricopeptide TPR-1, IPR006636 : Heat shock chaperonin-binding |
| MGG03508.6 | 0.26 | beta-glucosidase 1 precursor | IPR001764 : Glycoside hydrolase, family 3 |
| MGG00599.6 | 0.26 | 3-methyl-2-oxobutanoate hydroxymethyltransferase | IPR003700: Ketopantoate hydroxymethyltransferase, IPR015813: Pyruvate/Phosphoenolpyruvate kinase, catalytic core |
| MGG10487.6 | 0.26 | hypothetical protein | No defined Interpro term |
| MGG10826.6 | 0.26 | conserved hypothetical protein | IPR000719: Protein kinase, core, IPR00827 : Serine/threonine protein kinase, active site, IPR017441 : Protein kinase, ATP binding site |
| MGG02268.6 | 0.26 | conserved hypothetical protein | No defined Interpro term |
| MGG03045.6 | 0.26 | ankyrin repeat protein | IPR002110: Ankyrin |
| MGG05198.6 | 0.25 | conserved hypothetical protein | No defined Interpro term |
| MGG02818.6 | 0.25 | isoamyl alcohol oxidase | IPR006094: FAD linked oxidase, N-terminal, IPR012951: Berberine/berberine-like, IPR016166: FAD-binding, type 2 |
| MGG07787.6 | 0.25 | conserved hypothetical protein | No defined Interpro term |
| MGG00532.6 | 0.25 | conserved hypothetical protein | IPR000832: GPCR, family 2, secretin-like |
| MGG09032.6 | 0.25 | aspergillopepsin-2 | IPR000250: Peptidase G1, eqolisin, IPR008985: Concanavalin A-like lectin/glucanase |
| MGG02845.6 | 0.25 | hypothetical protein | IPR013087: Zinc finger, C2H2-type/integrase, DNA-binding |
| MGG06871.6 | 0.25 | conserved hypothetical protein | No defined Interpro term |
| MGG06682.6 | 0.25 | hypothetical protein | IPR002198: Short-chain dehydrogenase/reductase SDR ,IPR016040: NAD(P)-binding |
| MGG06390.6 | 0.25 | rho-GTPase-activating protein 5 | IPR000198 : RhoGAP, IPR008936: Rho GTPase activation protein |
| MGG00329.6 | 0.25 | conserved hypothetical protein | No defined Interpro term |
| MGG14179.6 | 0.25 | conserved hypothetical protein | No defined Interpro term |
| MGG05746.6 | 0.25 | canalicular multispecific organic anion transporter 1 | IPR001140: ABC transporter, transmembrane region, IPR003593: ATPase, AAA+ type, core, IPR011527: ABC transporter, transmembrane region, type 1, IPR017940: ABC transporter integral membrane type 1 |
| MGG06661.6 | 0.25 | high-affinity nicotinic acid transporter | IPR011701: Major facilitator superfamily MFS-1, IPR016196 : Major facilitator superfamily, general substrate transporter |
| MGG09677.6 | 0.24 | conserved hypothetical protein | IPR011118: Tannase and feruloyl esterase |
| MGG07697.6 | 0.24 | superoxide dismutase | IPR001189: Manganese and iron superoxide dismutase |
| MGG06851.6 | 0.24 | conserved hypothetical protein | No defined Interpro term |
| MGG03595.6 | 0.24 | G2/mitotic-specific cyclin-B1 | IPR004367: Cyclin, C-terminal, IPR006670 : Cyclin |
| MGG02976.6 | 0.24 | conserved hypothetical protein | ,IPR010730: Heterokaryon incompatibility, IPR011990: Tetratricopeptide-like helical |
| MGG15333.6 | 0.24 | conserved hypothetical protein |  |
| MGG12506.6 | 0.24 | hypothetical protein | No defined Interpro term |
| MGG08480.6 | 0.24 | alpha/beta hydrolase | IPR000073: Alpha/beta hydrolase fold-1 |
| MGG03995.6 | 0.24 | carboxypeptidase S1 | IPR001563: Peptidase S10, serine carboxypeptidase |
| MGG04869.6 | 0.23 | esterase/lipase/thioesterase | IPR013094: Alpha/beta hydrolase fold-3 |
| MGG07919.6 | 0.23 | hypothetical protein | No defined Interpro term |
| MGG00364.6 | 0.23 | smr domain-containing protein | IPR002625: Smr protein/MutS2 C-terminal, IPR013899: Region of unknown function DUF1771 |
| MGG13838.6 | 0.23 | NADPH-quinone reductase | IPR002085: Alcohol dehydrogenase superfamily, zinc-containing, IPR011032: GroES-like |
| MGG03407.6 | 0.23 | conserved hypothetical protein | No defined Interpro term |
| MGG09807.6 | 0.23 | hypothetical protein | No defined Interpro term |
| MGG05619.6 | 0.22 | alkaline phytoceramidase | IPR008901: Alkaline phytoceramidase |
| MGG01747.6 | 0.22 | nitric oxide synthase | IPR001433: Oxidoreductase FAD/NAD(P)-binding0, IPR003097: FAD-binding, type 1, IPR017927: Ferredoxin reductase-type FAD-binding domain, IPR017938: Riboflavin synthase-like beta-barrel |
| MGG02489.6 | 0.22 | branched-chain-amino-acid aminotransferase | IPR001544 : Aminotransferase, class IV, IPR005786: Branched-chain amino acid aminotransferase II |
| MGG09891.6 | 0.22 | double-strand-break repair protein rad21 | IPR001638: Extracellular solute-binding protein, family 3, IPR006910: Rad21/Rec8 like protein, N-terminal |
| MGG06504.6 | 0.22 | 26S proteasome regulatory subunit RPN7 | IPR000717 : Proteasome component region PCI |
| MGG11374.6 | 0.22 | alpha-galactosidase | IPR00011 : Glycoside hydrolase, clan GH-D, IPR002241: Glycoside hydrolase, family 27, IPR006215: Glycoside hydrolase, melibiase, IPR017853 : Glycoside hydrolase, catalytic core |
| MGG09785.6 | 0.22 | conserved hypothetical protein | IPR002198: Short-chain dehydrogenase/reductase SDR, IPR002347: Glucose/ribitol dehydrogenase, IPR016040 : NAD(P)-binding |
| MGG05485.6 | 0.22 | L-amino-acid oxidase precursor | IPR002937: Amine oxidase |
| MGG04544.6 | 0.22 | F1F0 ATP synthase assembly protein Atp11 | IPR010591: ATP11 |
| MGG15383.6 | 0.22 | glutamyl-tRNA(Gln) amidotransferase | IPR000120: Amidase signature enzyme |
| MGG08854.6 | 0.22 | CaaX protease | IPR003675: Abortive infection protein |
| MGG05283.6 | 0.22 | uricase | IPR002042: Uricase |
| MGG02920.6 | 0.22 | integral membrane protein sed5 | IPR000727: Target SNARE coiled-coil region, IPR006011: Syntaxin, N-terminal, IPR010989: t-SNARE |
| MGG04344.6 | 0.22 | conserved hypothetical protein | No defined Interpro term |
| MGG08822.6 | 0.21 | WW domain-containing protein | IPR001202: WW/Rsp5/WWP |
| MGG08724.6 | 0.21 | glycosyltransferase | IPR000111: Glycoside hydrolase, clan GH-D, IPR002213: UDP-glucuronosyl/UDP-glucosyltransferase, IPR006158 : Cobalamin (vitamin B12)-binding |
| MGG06665.6 | 0.21 | hypothetical protein | No defined Interpro term |
| MGG08851.6 | 0.21 | phosphatidyl synthase | IPR000462: CDP-alcohol phosphatidyltransferase, IPR016059: ATP-dependent DNA ligase, conserved site |
| MGG03438.6 | 0.21 | transporter | IPR005829 : Sugar transporter, conserved site |
| MGG10702.6 | 0.21 | conserved hypothetical protein | IPR004838: Aminotransferases, class-I, pyridoxal-phosphate-binding site,I IPR016196: Major facilitator superfamily, general substrate transporter |
| MGG01870.6 | 0.21 | hypothetical protein | IPR001138: Fungal transcriptional regulatory protein, N-terminal |
| MGG04589.6 | 0.21 | chromodomain helicase hrp3 | IPR000330: SNF2-related, IPR000953 : Chromo domain, IPR001650: DNA/RNA helicase, C-terminal, IPR014001 : DEAD-like helicase, N-terminal, IPR014021: Helicase, superfamily 1 and 2, ATP-binding, IPR016197: Chromo domain-like |
| MGG03593.6 | 0.21 | conserved hypothetical protein | No defined Interpro term |
| MGG08389.6 | 0.21 | conserved hypothetical protein | IPR002198: Short-chain dehydrogenase/reductase SDR, IPR002347: Glucose/ribitol dehydrogenase, IPR016040: NAD(P)-binding |
| MGG04369.6 | 0.21 | spastin | IPR003593: ATPase, AAA+ type, core, IPR003959: ATPase, AAA-type, core, |
| MGG07528.6 | 0.21 | imidazoleglycerol-phosphate dehydratase | IPR000807: Imidazole glycerol-phosphate dehydratase |
| MGG07109.6 | 0.21 | eukaryotic translation initiation factor 3 subunit G | IPR000504: RNA recognition motif, RNP-1, IPR012677: Nucleotide-binding, alpha-beta plait, IPR017334: Translation initiation factor 3, RNA-binding subunit |
| MGG05197.6 | 0.21 | serine palmitoyltransferase 2 | IPR004839: Aminotransferase, class I and II, IPR015421: Pyridoxal phosphate-dependent transferase, major region, subdomain 1 |
| MGG11021.6 | 0.20 | conserved hypothetical protein | IPR003593 : ATPase, AAA+ type, core,IPR003959 : ATPase, AAA-type, core |
| MGG03276.6 | 0.20 | major allergen Asp f 2 | No defined Interpro term |
| MGG09332.6 | 0.20 | hypothetical protein | No defined Interpro term |
| MGG04456.6 | 0.20 | zinc finger protein 664 | IPR013087 : Zinc finger, C2H2-type/integrase, DNA-binding |
| MGG00010.6 | 0.20 | conserved hypothetical protein | IPR016196 : Major facilitator superfamily, general substrate transporter |
| MGG02041.6 | 0.20 | hypothetical protein | No defined Interpro term |
| MGG06476.6 | 0.20 | conserved hypothetical protein | IPR003123: Vacuolar sorting protein 9 |
| MGG02632.6 | 0.20 | conserved hypothetical protein | IPR001159: Double-stranded RNA binding, IPR004827: Basic-leucine zipper (bZIP) transcription factor |
| MGG00350.6 | 0.20 | conserved hypothetical protein | IPR007858: Dpy-30, conserved site |
| MGG06937.6 | 0.20 | NIF domain protein | IPR004274: NLI interacting factor |
| MGG05937.6 | 0.19 | dynamin family protein | IPR001401: Dynamin, GTPase region |
| MGG14890.6 | 0.19 | conserved hypothetical protein | IPR011042: Six-bladed beta-propeller, TolB-like, IPR013658 : SMP-30/Gluconolaconase/LRE-like region |
| MGG04943.6 | 0.19 | mitogen-activated protein kinase spm1 | IPR000719 : Protein kinase, core, IPR002290 : Serine/threonine protein kinase, IPR003527 : MAP kinase, conserved site, IPR017441 : Protein kinase, ATP binding site |
| MGG08020.6 | 0.19 | endoglucanase-4 | IPR005103: Glycoside hydrolase, family 61 |
| MGG13026.6 | 0.19 | hypothetical protein | No defined Interpro term |
| MGG12712.6 | 0.19 | conserved hypothetical protein | No defined Interpro term |
| MGG13262.6 | 0.19 | FAD binding domain-containing protein | IPR006094: FAD linked oxidase, N-terminal, IPR016166: FAD-binding, type 2 |
| MGG12214.6 | 0.19 | fatty acid synthase S-acetyltransferase | IPR000794: Beta-ketoacyl synthase, IPR001227: Acyl transferase region, IPR006163: Phosphopantetheine-binding, IPR011032: GroES-like, IPR013149: Alcohol dehydrogenase, zinc-binding, IPR013217: Methyltransferase type 12 ,IPR013968: Polyketide synthase, KR, IPR016036: Malonyl-CoA ACP transacylase, ACP-binding, IPR016040: NAD(P)-binding |
| MGG11496.6 | 0.19 | conserved hypothetical protein | No defined Interpro term |
| MGG08279.6 | 0.19 | inner membrane protein yicO | IPR006043: Xanthine/uracil/vitamin C permease |
| MGG11604.6 | 0.19 | conserved hypothetical protein | IPR000215: Protease inhibitor I4, serpin, IPR001810: Cyclin-like F-box |
| MGG10704.6 | 0.19 | hypothetical protein | IPR00018 : GCN5-related N-acetyltransferase, IPR016181: Acyl-CoA N-acyltransferase |
| MGG09198.6 | 0.19 | ent-kaurene oxidase | IPR001128 : Cytochrome P450, IPR002403 : Cytochrome P450, E-class, group IV |
| MGG12118.6 | 0.18 | serine/threonine-protein kinase 24 | IPR000719: Protein kinase, core, IPR001245: Tyrosine protein kinase, IPR002290: Serine/threonine protein kinase, IPR017441 : Protein kinase, ATP binding site |
| MGG10162.6 | 0.18 | conserved hypothetical protein | IPR007087: Zinc finger, C2H2-type |
| MGG00702.6 | 0.18 | hypothetical protein | No defined Interpro term |
| MGG13262.6 | 0.18 | FAD binding domain-containing protein |  |
| MGG04961.6 | 0.18 | conserved hypothetical protein | No defined Interpro term |
| MGG01692.6 | 0.18 | conserved hypothetical protein | IPR001148: Carbonic anhydrase, alpha-class, catalytic domain |
| MGG02961.6 | 0.18 | anucleate primary sterigmata protein A | IPR001849: Pleckstrin homology |
| MGG04361.6 | 0.17 | conserved hypothetical protein | No defined Interpro term |
| MGG04969.6 | 0.17 | cytochrome c oxidase assembly protein COX15 | IPR003780: Cytochrome oxidase assembly |
| MGG02156.6 | 0.17 | iron transport multicopper oxidase FET5 | IPR001117: Multicopper oxidase, type 1, IPR00235 : Multicopper oxidase, copper-binding site, IPR008972: Cupredoxin, |
| MGG07973.6 | 0.17 | surface protein 1 | No defined Interpro term |
| MGG15060.6 | 0.17 | conserved hypothetical protein | IPR011701: Major facilitator superfamily MFS-1, IPR016196: Major facilitator superfamily, general substrate transporter |
| MGG05051.6 | 0.17 | hypothetical protein | No defined Interpro term |
| MGG02987.6 | 0.17 | para-nitrobenzyl esterase | IPR002018: Carboxylesterase, type B |
| MGG09131.6 | 0.17 | alpha/beta hydrolase | IPR000073: Alpha/beta hydrolase fold-1 |
| MGG15388.6 | 0.17 | lactose permease | IPR003663: Sugar/inositol transporter, IPR005828 : General substrate transporter |
| MGG00271.6 | 0.17 | conserved hypothetical protein | No defined Interpro term |
| MGG03170.6 | 0.17 | conserved hypothetical protein | No defined Interpro term |
| MGG03030.6 | 0.17 | conserved hypothetical protein | IPR007087: Zinc finger, C2H2-type, IPR015880: Zinc finger, C2H2-like |
| MGG09972.6 | 0.17 | conserved hypothetical protein | IPR005829 : Sugar transporter, conserved site, IPR011701 : Major facilitator superfamily MFS-1 |
| MGG08158.6 | 0.16 | hypothetical protein | No defined Interpro term |
| MGG06593.6 | 0.16 | endo-1,4-beta-xylanase 2 | IPR001137: Glycoside hydrolase, family 11, IPR008985: Concanavalin A-like lectin/glucanase, IPR013319: Glycoside hydrolase, families 11 and 12, catalytic core |
| MGG03241.6 | 0.16 | arrestin domain-containing protein | IPR011022: Arrestin-like, C-terminal |
| MGG05798.6 | 0.16 | conserved hypothetical protein | IPR000675: Cutinase, IPR011150: Cutinase, monofunctional |
| MGG03370.6 | 0.16 | C-4 methylsterol oxidase | IPR006088 |
| MGG03446.6 | 0.16 | conserved hypothetical protein | No defined Interpro term |
| MGG04338.6 | 0.15 | potassium transport protein 1 | IPR003445: Cation transporter |
| MGG10286.6 | 0.15 | conserved hypothetical protein | No defined Interpro term |
| MGG03625.6 | 0.15 | conserved hypothetical protein | No defined Interpro term |
| MGG06070.6 | 0.15 | serine/threonine-protein kinase SAPK1 | IPR000719: Protein kinase, core, IPR008271: Serine/threonine protein kinase, active site, IPR017441: Protein kinase, ATP binding site |
| MGG01905.6 | 0.15 | conserved hypothetical protein | No defined Interpro term |
| MGG02759.6 | 0.15 | conserved hypothetical protein | No defined Interpro term |
| MGG01427.6 | 0.15 | SET1 complex component ash2 | IPR001870: B302 (SPRY)-like, IPR003877 : SPla/RYanodine receptor SPRY |
| MGG07648.6 | 0.15 | hypothetical protein | No defined Interpro term |
| MGG04952.6 | 0.15 | hypothetical protein | No defined Interpro term |
| MGG10456.6 | 0.15 | hypothetical protein | No defined Interpro term |
| MGG03207.6 | 0.14 | protein kinase domain-containing protein | IPR000719: Protein kinase, core |
| MGG13218.6 | 0.14 | pyruvate formate lyase activating enzyme |  |
| MGG10490.6 | 0.14 | GPI-anchor transamidase | IPR001096: Peptidase C13, legumain |
| MGG15364.6 | 0.14 | hypothetical protein | No defined Interpro term |
| MGG05844.6 | 0.14 | mannan endo-1,4-beta-mannosidase 1 precursor | IPR001547: Glycoside hydrolase, family 5, IPR013781: Glycoside hydrolase, subgroup, catalytic core |
| MGG02383.6 | 0.14 | hypothetical protein | No defined Interpro term |
| MGG04101.6 | 0.14 | hypothetical protein | No defined Interpro term |
| MGG08130.6 | 0.14 | conserved hypothetical protein | IPR001138: Fungal transcriptional regulatory protein, N-terminal, IPR007219: Fungal specific transcription factor |
| MGG04550.6 | 0.14 | calcium-transporting ATPase sarcoplasmic/endoplasmic reticulum type | IPR001757 : ATPase, P-type, K/Mg/Cd/Cu/Zn/Na/Ca/Na/H-transporter, IPR004014 : ATPase, P-type cation-transporter |
| MGG06624.6 | 0.14 | hypothetical protein | No defined Interpro term |
| MGG07971.6 | 0.13 | calcium-transporting ATPase 1 | IPR001757: ATPase, P-type, K/Mg/Cd/Cu/Zn/Na/Ca/Na/H-transporter, IPR004014: ATPase, P-type cation-transporter, N-terminal, IPR006068: ATPase, P-type cation-transporter, C-terminal, IPR006408: ATPase, P-type, calcium-transporting, PMCA-type |
| MGG15188.6 | 0.13 | conserved hypothetical protein | No defined Interpro term |
| MGG05871.6 | 0.13 | integral membrane protein | IPR008427: Extracellular membrane protein, 8-cysteine region, CFEM |
| MGG04864.6 | 0.13 | allantoin permease | IPR001248: Permease for cytosine/purines, uracil, thiamine, allantoin |
| MGG10661.6 | 0.13 | hypothetical protein | No defined Interpro term |
| MGG03081.6 | 0.13 | conserved hypothetical protein | IPR002654: Glycosyl transferase, family 25 |
| MGG13328.6 | 0.13 | RING finger membrane protein | No defined Interpro term |
| MGG07501.6 | 0.13 | ATP-dependent RNA helicase DHX8 | IPR001650: DNA/RNA helicase, C-terminal, IPR002464 : DNA/RNA helicase, ATP-dependent, DEAH-box type, conserved site, IPR007502: Helicase-associated region, IPR014021 : Helicase, superfamily 1 and 2, ATP-binding |
| MGG01432.6 | 0.13 | conserved hypothetical protein | IPR000555: Mov34/MPN/PAD-1 |
| MGG05787.6 | 0.13 | conserved hypothetical protein | IPR001128: Cytochrome P450, IPR002401 : Cytochrome P450, E-class, group I |
| MGG04988.6 | 0.13 | chromosome segregation protein sudA | ,IPR010935: SMCs flexible hinge |
| MGG05157.6 | 0.13 | conserved hypothetical protein | IPR011008: Dimeric alpha-beta barrel |
| MGG05038.6 | 0.13 | endonuclease/exonuclease/phosphatase family protein | IPR005135: Endonuclease/exonuclease/phosphatase |
| MGG10583.6 | 0.12 | 12-oxophytodienoate reductase 1 | IPR001155: NADH:flavin oxidoreductase/NADH oxidase, N-terminal, IPR013785: Aldolase-type TIM barrel |
| MGG09082.6 | 0.12 | conserved hypothetical protein | No defined Interpro term |
| MGG06144.6 | 0.12 | hypothetical protein |  |
| MGG07958.6 | 0.12 | naringenin,2-oxoglutarate 3-dioxygenase | IPR005123: 2OG-Fe(II) oxygenase |
| MGG04017.6 | 0.12 | L-asparaginase | IPR002110: Ankyrin, IPR006034 : Asparaginase/glutaminase |
| MGG08218.6 | 0.12 | hypothetical protein | No defined Interpro term |
| MGG14901.6 | 0.12 | conserved hypothetical protein | No defined Interpro term |
| MGG07850.6 | 0.12 | conserved hypothetical protein | No defined Interpro term |
| MGG01581.6 | 0.12 | 26S protease regulatory subunit 7 | IPR003593: ATPase, AAA+ type, core, IPR003959: ATPase, AAA-type, core, IPR005937: 26S proteasome subunit P45 |
| MGG09549.6 | 0.12 | conserved hypothetical protein | No defined Interpro term |
| MGG01572.6 | 0.12 | DNA-directed RNA polymerase I subunit RPA2 | IPR007120: DNA-directed RNA polymerase, subunit 2, domain 6, IPR007121: RNA polymerase, beta subunit, conserved site, IPR007641: RNA polymerase Rpb2, domain 7, IPR007642: RNA polymerase Rpb2, domain 2, IPR007644: RNA polymerase, beta subunit, protrusion, IPR007645: RNA polymerase Rpb2, domain 3, IPR007647: RNA polymerase Rpb2, domain 5, IPR009674: RNA polymerase I, Rpa2 specific, IPR015712: DNA-directed RNA polymerase, subunit 2 |
| MGG06955.6 | 0.12 | conserved hypothetical protein | No defined Interpro term |
| MGG12317.6 | 0.12 | hypothetical protein | No defined Interpro term |
| MGG13764.6 | 0.12 | bilirubin oxidase | IPR008972: Cupredoxin, IPR011706: Multicopper oxidase, type 2, IPR011707: Multicopper oxidase, type 3 |
| MGG10760.6 | 0.12 | FAD-binding domain-containing protein | IPR013112: FAD-binding 8, IPR013130: Ferric reductase-like transmembrane component, N-terminal, IPR017927: Ferredoxin reductase-type FAD-binding domain |
| MGG01952.6 | 0.12 | conserved hypothetical protein | No defined Interpro term |
| MGG09817.6 | 0.11 | minor extracellular protease vpr | IPR000209: Peptidase S8 and S53, subtilisin, kexin, sedolisin ,IPR003137: Protease-associated PA |
| MGG00099.6 | 0.11 | conserved hypothetical protein | IPR006353: HAD-superfamily hydrolase, subfamily IIA, CECR5, IPR006357: HAD-superfamily hydrolase, subfamily IIA |
| MGG09795.6 | 0.11 | conserved hypothetical protein | IPR006461: Protein of unknown function Cys-rich |
| MGG07964.6 | 0.11 | conserved hypothetical protein | IPR013217: Methyltransferase type 12 |
| MGG06635.6 | 0.11 | hypothetical protein | No defined Interpro term |
| MGG01941.6 | 0.11 | FAD binding domain-containing protein | IPR006094: FAD linked oxidase, N-terminal, IPR016166: FAD-binding, type 2 ,IPR016168: FAD-linked oxidase, FAD-binding, subdomain 2 |
| MGG00634.6 | 0.11 | nitrite reductase | IPR001327: Pyridine nucleotide-disulphide oxidoreductase, NAD-binding region, , IPR005806: Rieske [2Fe-2S] region, IPR006066 : Nitrite and sulphite reductase iron-sulphur/siroheme-binding site,IPR006067 : Nitrite and sulphite reductase 4Fe-4S region, IPR007419: BFD-like [2Fe-2S]-binding region, IPR012744: Nitrite reductase [NAD(P)H] large subunit, NirB, IPR013027: FAD-dependent pyridine nucleotide-disulphide oxidoreductase |
| MGG07824.6 | 0.11 | spherulin-1B | IPR001929: Germin, IPR006045 : Cupin 1, IPR011051: Cupin, RmlC-type1, IPR014710: RmlC-like jelly roll fold |
| MGG09165.6 | 0.11 | hypothetical protein | No defined Interpro term |
| MGG01862.6 | 0.11 | zinc/iron transporter protein | IPR003689: Zinc/iron permease |
| MGG13261.6 | 0.11 | conserved hypothetical protein | IPR008972: Cupredoxin |
| MGG10429.6 | 0.11 | conserved hypothetical protein | No defined Interpro term |
| MGG00311.6 | 0.11 | conserved hypothetical protein | IPR000250: Peptidase G1, eqolisin, IPR008985: Concanavalin A-like lectin/glucanase |
| MGG07305.6 | 0.11 | hypothetical protein |  |
| MGG07100.6 | 0.10 | hypothetical protein | No defined Interpro term |
| MGG06696.6 | 0.10 | ethylene receptor | IPR000719: Protein kinase, core ,IPR001789 : Signal transduction response regulator, receiver region ,IPR002290: Serine/threonine protein kinase, IPR003018: GAF, IPR003594: ATP-binding region, ATPase-like, IPR003661 : Signal transduction histidine kinase, subgroup 1, dimerisation and phosphoacceptor region, IPR005467: Signal transduction histidine kinase, core, IPR005467: Signal transduction histidine kinase, core, IPR009082: Signal transduction histidine kinase, homodimeric |
| MGG09608.6 | 0.10 | conserved hypothetical protein | IPR000743: Glycoside hydrolase, family 28, IPR011050: Pectin lyase fold/virulence factor, IPR012334 : Pectin lyase fold |
| MGG08501.6 | 0.10 | conserved hypothetical protein | No defined Interpro term |
| MGG10799.6 | 0.10 | acid phosphatase PHO1 | IPR000560: Histidine acid phosphatase |
| MGG12154.6 | 0.10 | fatty acid synthase subunit alpha reductase | IPR004568: Phosphopantethiene-protein transferase, IPR006162: Phosphopantetheine attachment site, IPR008278 : 4'-phosphopantetheinyl transferase, IPR014030: Beta-ketoacyl synthase, N-terminal, IPR014031: Beta-ketoacyl synthase, C-terminal, IPR016035: Acyl transferase/acyl hydrolase/lysophospholipase, IPR016039: Thiolase-liken, IPR016040: NAD(P)-binding, |
| MGG02487.6 | 0.10 | calcium-transporting ATPase 2 (Vacuolar Ca(2+)-ATPase) | IPR001757: ATPase, P-type, K/Mg/Cd/Cu/Zn/Na/Ca/Na/H-transporter,ATPase, P-type cation-transporter, N-terminal, IPR004014: ATPase, P-type cation-transporter, N-terminal, IPR006068: ATPase, P-type cation-transporter, C-terminal, IPR006408: ATPase, P-type, calcium-transporting, PMCA-type, IPR008162: Inorganic pyrophosphatase |
| MGG00251.6 | 0.10 | dimethyladenosine transferase | IPR001737: Ribosomal RNA adenine methylase transferase, IPR011530: rRNA adenine dimethylase |
| MGG13861.6 | 0.10 | conserved hypothetical protein | IPR001077: O-methyltransferase, family 2 |
| MGG15333.6 | 0.10 | conserved hypothetical protein | IPR001077: O-methyltransferase, family 2, IPR016461: O-methyltransferase, COMT, eukaryota |
| MGG07631.6 | 0.10 | fungal cellulose binding domain-containing protein | No defined Interpro term |
| MGG06234.6 | 0.09 | hypothetical protein | No defined Interpro term |
| MGG15435.6 | 0.09 | hypothetical protein 2 | IPR011701: Major facilitator superfamily MFS-1, IPR016196: Major facilitator superfamily, general substrate transporter |
| MGG07305.6 | 0.09 | bZIP transcription factor | IPR004827: Basic-leucine zipper (bZIP) transcription factor |
| MGG13793.6 | 0.09 | nitrate transporter | IPR004737 : Nitrate transporter, IPR011701: Major facilitator superfamily MFS-1, IPR016196 : Major facilitator superfamily, general substrate transporter |
| MGG06330.6 | 0.09 | conserved hypothetical protein | No defined Interpro term |
| MGG13764.6 | 0.09 | bilirubin oxidase |  |
| MGG06799.6 | 0.09 | conserved hypothetical protein | No defined Interpro term |
| MGG00097.6 | 0.09 | conserved hypothetical protein | IPR001395: Aldo/keto reductase |
| MGG04332.6 | 0.09 | hypothetical protein | No defined Interpro term |
| MGG13177.6 | 0.09 | cytosolic Cu/Zn superoxide dismutase | IPR001424: Superoxide dismutase, copper/zinc binding |
| MGG07274.6 | 0.09 | conserved hypothetical protein | No defined Interpro term |
| MGG14883.6 | 0.09 | methyltransferase small domain-containing protein | IPR013216: Methyltransferase type 11,IPR013216 : Methyltransferase type 11 |
| MGG04985.6 | 0.09 | conserved hypothetical protein | IPR001313: Pumilio RNA-binding region, IPR016024 : Armadillo-type fold |
| MGG04582.6 | 0.08 | conserved hypothetical protein | IPR013781: Glycoside hydrolase, subgroup, catalytic core, IPR017853: Glycoside hydrolase, catalytic core |
| MGG01742.6 | 0.08 | elongation factor 2 | IPR000640 : Translation elongation factor EFG/EF2, C-terminal ,IPR000795: Protein synthesis factor, GTP-binding, IPR004161 : Translation elongation factor EFTu/EF1A, domain 2, IPR009000: Translation elongation and initiation factors/Ribosomal, beta-barrel |
| MGG14603.6 | 0.08 | hypothetical protein | No defined Interpro term |
| MGG01444.6 | 0.08 | hypothetical protein | No defined Interpro term |
| MGG05349.6 | 0.08 | conserved hypothetical protein | IPR002123: Phospholipid/glycerol acyltransferase |
| MGG09354.6 | 0.07 | peptide transporter MTD1 | IPR004813: Oligopeptide transporter OPT superfamily |
| MGG07766.6 | 0.07 | hypothetical protein | No defined Interpro term |
| MGG09321.6 | 0.07 | conserved hypothetical protein | No defined Interpro term |
| MGG01063.6 | 0.07 | conserved hypothetical protein | No defined Interpro term |
| MGG04339.6 | 0.07 | conserved hypothetical protein | No defined Interpro term |
| MGG14517.6 | 0.07 | developmental regulator flbA | IPR000342: Regulator of G protein signalling, IPR000591: Pleckstrin/G-protein, interacting region, IPR016137: Regulator of G protein signalling superfamily |
| MGG04406.6 | 0.07 | 26S proteasome non-ATPase regulatory subunit 10 | IPR002078 : RNA polymerase sigma factor 54, interaction,IPR002110 : Ankyrin |
| MGG02059.6 | 0.07 | conserved hypothetical protein | No defined Interpro term |
| MGG06169.6 | 0.06 | conserved hypothetical protein | IPR002227: Tyrosinase, IPR008922 : Di-copper centre-containing |
| MGG06782.6 | 0.06 | N amino acid transport system protein | IPR013057: Amino acid transporter, transmembrane |
| MGG07523.6 | 0.06 | conserved hypothetical protein | IPR005062: SAC3/GANP/Nin1/mts3/eIF-3 p25 |
| MGG08185.6 | 0.06 | conserved hypothetical protein | IPR001138: Fungal transcriptional regulatory protein, N-terminal |
| MGG08757.6 | 0.06 | DNA binding regulatory protein AmdX | IPR013087: Zinc finger, C2H2-type/integrase, DNA-binding, IPR015880: Zinc finger, C2H2-like |
| MGG05789.6 | 0.06 | hypothetical protein | No defined Interpro term |
| MGG01134.6 | 0.04 | UTR2 protein | IPR000757: Glycoside hydrolase, family 16, IPR008197: Whey acidic protein, 4-disulphide core |
| MGG15264.6 | 0.04 | hypothetical protein | No defined Interpro term |
| MGG01129.6 | 0.04 | hypothetical protein | No defined Interpro term |
| MGG15264.6 | 0.04 | hypothetical protein |  |
| MGG07705.6 | 0.03 | acyl-CoA ligase | IPR000873: AMP-dependent synthetase and ligase, IPR010506: DMAP1-binding |
| MGG09445.6 | 0.01 | conserved hypothetical protein | IPR000008: C2 calcium-dependent membrane targeting, IPR008973: C2 calcium/lipid-binding region, CaLB |

^a^ Reduction ratios from the microarray analysis are calculated as the expression in conidiating mycelia (CNMY) divided by non-conidiating mycelia (NCMY) of the wild-type.
