# Supplementary material for: Gene Expression Profiling during Conidiation in the Rice Blast Pathogen Magnaporthe oryzae
Source: PLoS One. 2012 Aug 21;7(8):e43202. doi: 10.1371/journal.pone.0043202 (PMC3424150; doi:10.1371/journal.pone.0043202)
Supplement: Table S5 — Genes repressed during conidiation in the ΔMohox2 mutant. (DOCX) [file pone.0043202.s005.docx]

**Table S5.** Genes repressed during conidiation in the ΔMohox2 mutant.

| **Locus** | **Fold-reduction during conidiation in the *ΔMohox2* mutant^a^** | **Annotation** | **InterPro domain search** |
| --- | --- | --- | --- |
| MGG00618.6 | 0.50 | pectinesterase | IPR000070 : Pectinesterase, catalytic, IPR011050 : Pectin lyase fold/virulence factor |
| MGG01174.6 | 0.50 | conserved hypothetical protein | IPR005373 : Uncharacterised protein family UPF0183 |
| MGG01428.6 | 0.50 | conserved hypothetical protein | No defined Interpro term |
| MGG13219.6 | 0.50 | DUF323 domain-containing protein | IPR005532 : Sulfatase-modifying factor, IPR017805 : SAM-dependent methyltransferase, EsaF-type, putative |
| MGG00722.6 | 0.50 | hydantoinase |  |
| MGG01769.6 | 0.50 | conserved hypothetical protein | IPR007632 : Protein of unknown function DUF590 |
| MGG05328.6 | 0.49 | peroxin 8 | IPR008984 : SMAD/FHA domain |
| MGG03620.6 | 0.49 | hexose transporter 2 | IPR003663 : Sugar/inositol transporter, IPR005828 : General substrate transporter, IPR006162 : Phosphopantetheine attachment site |
| MGG03349.6 | 0.49 | auxin Efflux Carrier superfamily | IPR004776 : Auxin efflux carrier |
| MGG04166.6 | 0.49 | salicylate hydroxylase | IPR003042 : Aromatic-ring hydroxylase-like, IPR013027 : FAD-dependent pyridine nucleotide-disulphide oxidoreductase |
| MGG10619.6 | 0.49 | conserved hypothetical protein | IPR000182 : GCN5-related N-acetyltransferase, IPR016181 : Acyl-CoA N-acyltransferase |
| MGG00645.6 | 0.49 | phosphopantothenoylcysteine decarboxylase | IPR003382 : Flavoprotein |
| MGG09199.6 | 0.49 | repressible acid phosphatase | IPR000560 : Histidine acid phosphatase, IPR016274 : Histidine acid phosphatase, eukaryotic |
| MGG01257.6 | 0.48 | tRNA-dihydrouridine synthase 4 | IPR001269 : tRNA-dihydrouridine synthase, IPR013785 : Aldolase-type TIM barrel |
| MGG08648.6 | 0.48 | anaphase-promoting complex subunit 8 | IPR001440 : Tetratricopeptide TPR-1, IPR007192 : Cdc23, IPR013026 : Tetratricopeptide region |
| MGG05058.6 | 0.48 | hypothetical protein | No defined Interpro term |
| MGG00275.6 | 0.48 | MFS transporter | IPR011701 : Major facilitator superfamily MFS-1, IPR016196 : Major acilitator superfamily, general substrate transporter |
| MGG05218.6 | 0.48 | conserved hypothetical protein | No defined Interpro term |
| MGG11536.6 | 0.48 | alpha-xylosidase | IPR000322 : Glycoside hydrolase, family 31, IPR017853 : Glycoside hydrolase, catalytic core |
| MGG09710.6 | 0.48 | conserved hypothetical protein | No defined Interpro term |
| MGG09512.6 | 0.48 | 3-ketoacyl-CoA thiolase | IPR002155 : Thiolase, IPR016039 : Thiolase-like |
| MGG03287.6 | 0.48 | alpha-amylase | IPR006047 : Glycosyl hydrolase, family 13, catalytic region, IPR013776 : Alpha-amylase, thermostable |
| MGG06475.6 | 0.47 | alpha-actinin | IPR001589 : Actinin-type, actin-binding, conserved site, IPR001715 : Calponin-like actin-binding, IPR002048 : Calcium-binding EF-hand |
| MGG11210.6 | 0.47 | beta-glucosidase 1 | IPR001764 : Glycoside hydrolase, family 3, N-terminal, IPR002772 : Glycoside hydrolase, family 3, C-terminal, IPR017853 : Glycoside hydrolase, catalytic core |
| MGG07271.6 | 0.47 | mitochondrial ribosomal small subunit component | IPR017081 : Ribosomal protein S24, mitochondrial |
| MGG01822.6 | 0.47 | mitogen-activated protein kinase HOG1 | IPR000719 : Protein kinase, core, IPR002290 : Serine/threonine protein kinase, IPR003527 : MAP kinase, conserved site |
| MGG09471.6 | 0.47 | neutral trehalase | IPR001661 : Glycoside hydrolase, family 37, IPR008928 : Six-hairpin glycosidase-like, IPR011120 : Neutral trehalase Ca2+ binding |
| MGG01087.6 | 0.47 | ribosome biogenesis protein TSR1 | IPR007034 : Protein of unknown function DUF663, IPR012948 : AARP2CN |
| MGG01380.6 | 0.47 | 26S protease regulatory subunit 8 | IPR003593 : ATPase, AAA+ type, core, IPR003959 : ATPase, AAA-type, core, IPR005937 : 26S proteasome subunit P45 |
| MGG01328.6 | 0.47 | endoglucanase | IPR000334 : Glycoside hydrolase, family 45, IPR009009 : Barwin-related endoglucanase |
| MGG13641.6 | 0.47 | peroxin 23 | IPR006613 : Dysferlin, N-terminal, IPR010482 : Integral peroxisomal membrane peroxin |
| MGG01456.6 | 0.47 | conserved hypothetical protein | No defined Interpro term |
| MGG04611.6 | 0.47 | carbonic anhydrase | IPR001765 : Carbonic anhydrase, IPR015892 : Carbonic anhydrase, prokaryotic-like, conserved site |
| MGG06266.6 | 0.47 | eukaryotic peptide chain release factor subunit 1 | IPR004403 : Peptide chain release factor eRF/aRF subunit 1, IPR005140 : eRF1 domain 1, IPR005141 : eRF1 domain 2, |
| MGG00245.6 | 0.47 | conserved hypothetical protein | No defined Interpro term |
| MGG09351.6 | 0.46 | aspergillopepsin-F | IPR001461 : Peptidase A1, IPR001969 : Peptidase aspartic, active site, IPR009007 : Peptidase aspartic, catalytic |
| MGG03451.6 | 0.46 | Transcription factor | IPR007087 : Zinc finger, C2H2-type |
| MGG11429.6 | 0.46 | oligonucleotide transporter | IPR004813 : Oligopeptide transporter OPT superfamily |
| MGG09531.6 | 0.45 | rho-GTPase-activating protein 8 | IPR000198 : RhoGAP, IPR000591 : Pleckstrin/G-protein, interacting region, IPR001060 : Fps/Fes/Fer/CIP4 homology |
| MGG04710.6 | 0.45 | translational activator GCN1 | IPR000357 : HEAT,IPR000357 : HEAT, IPR011989 : Armadillo-like helical, IPR016024 : Armadillo-type fold |
| MGG10510.6 | 0.45 | ribonuclease T2 | IPR001568 : Ribonuclease T2 |
| MGG04007.6 | 0.45 | conserved hypothetical protein | IPR008914 : Phosphatidylethanolamine-binding protein PEBP |
| MGG06064.6 | 0.45 | chitin synthase D | IPR004835 : Fungal chitin synthase |
| MGG03197.6 | 0.45 | conserved hypothetical protein | IPR013861 : Protein of unknown function DUF1751, integral membrane, eukaryotic |
| MGG09729.6 | 0.45 | hypothetical protein | No defined Interpro term |
| MGG09107.6 | 0.45 | conserved hypothetical protein | IPR001202 : WW/Rsp5/WWP IPR010730 : Heterokaryon incompatibility |
| MGG07402.6 | 0.44 | conserved hypothetical protein | IPR015075 : Protein of unknown function DUF1857 |
| MGG01524.6 | 0.44 | 54S ribosomal protein L3 | IPR000999 : Ribonuclease III, IPR001159 : Double-stranded RNA binding, IPR014720 : Double-stranded RNA-binding-like |
| MGG02797.6 | 0.44 | conserved hypothetical protein | No defined Interpro term |
| MGG04469.6 | 0.44 | cytochrome P450 97B3 | IPR001128 : Cytochrome P450 |
| MGG07730.6 | 0.43 | DNL zinc finger domain-containing protein | IPR007853 : Zinc finger, Zim17-type |
| MGG08360.6 | 0.43 | DUF341 domain-containing protein | No defined Interpro term |
| MGG03555.6 | 0.43 | hypothetical protein | No defined Interpro term |
| MGG10123.6 | 0.43 | deamidase | IPR003010 : Nitrilase/cyanide hydratase and apolipoprotein N-acyltransferase |
| MGG11286.6 | 0.43 | oxidoreductase | IPR006620 : Prolyl 4-hydroxylase, alpha subunit |
| MGG13927.6 | 0.43 | hypothetical protein | IPR001138 : Fungal transcriptional regulatory protein, N-terminal |
| MGG03191.6 | 0.43 | katanin p60 ATPase-containing subunit | IPR003593 : ATPase, AAA+ type, core, IPR003959 : ATPase, AAA-type, core, IPR003960 : ATPase, AAA-type, conserved site |
| MGG01725.6 | 0.43 | deoxyhypusine hydroxylase | IPR004155 : PBS lyase HEAT-like repeat, IPR011989 : Armadillo-like helical, IPR016024 : Armadillo-type fold |
| MGG04132.6 | 0.42 | RNA exonuclease 4 | IPR006055 : Exonuclease, IPR012337 : Polynucleotidyl transferase, Ribonuclease H fold, IPR013520 : Exonuclease, RNase T and DNA polymerase III |
| MGG15100.6 | 0.42 | polyketide synthase | IPR000794 : Beta-ketoacyl synthase, IPR001227 : Acyl transferase region, IPR001242 : Condensation domain, IPR006163 : Phosphopantetheine-binding |
| MGG01867.6 | 0.42 | conserved hypothetical protein | No defined Interpro term |
| MGG04725.6 | 0.42 | leucine-rich repeat-containing protein 28 | IPR000719 : Protein kinase, core, IPR001245 : Tyrosine protein kinase, IPR001611 : Leucine-rich repeat |
| MGG08377.6 | 0.42 | conserved hypothetical protein | IPR001077 : O-methyltransferase, family 2, IPR011991 : Winged helix repressor DNA-binding |
| MGG02332.6 | 0.42 | conserved hypothetical protein | IPR008701 : Necrosis inducing |
| MGG04453.6 | 0.42 | mitochondrial ATPase complex subunit ATP10 | IPR007849 : ATPase assembly factor ATP10, mitochondria |
| MGG15327.6 | 0.42 | hypothetical protein | No defined Interpro term |
| MGG10268.6 | 0.42 | MAP kinase kinase PBS2 | IPR000719 : Protein kinase, core, IPR011009 : Protein kinase-like, IPR017442 : Serine/threonine protein kinase-related |
| MGG05632.6 | 0.42 | conserved hypothetical protein | No defined Interpro term |
| MGG00786.6 | 0.41 | conserved hypothetical protein | No defined Interpro term |
| MGG11311.6 | 0.41 | conserved hypothetical protein | No defined Interpro term |
| MGG05150.6 | 0.41 | conserved hypothetical protein | IPR016805 : Uncharacterised conserved protein UCP022603 |
| MGG06007.6 | 0.41 | hypothetical protein | No defined Interpro term |
| MGG07123.6 | 0.41 | ATP-dependent protease La 2 | IPR001984 : Peptidase S16, Lon protease, C-terminal region, IPR003593 : ATPase, AAA+ type, core, IPR004815 : Peptidase S16, ATP-dependent protease La |
| MGG03956.6 | 0.41 | conserved hypothetical protein | IPR003195 : Transcription initiation factor IID, 18 kDa subunit, IPR009072 : Histone-fold |
| MGG03186.6 | 0.41 | 1,4-alpha-glucan-branching enzyme | IPR004193 : Glycoside hydrolase, family 13, N-terminal, IPR006048 : Alpha-amylase, C-terminal all beta, IPR013783 : Immunoglobulin-like fold |
| MGG09569.6 | 0.41 | hypothetical protein | No defined Interpro term |
| MGG01390.6 | 0.41 | MFS hexose transporter | IPR003663 : Sugar/inositol transporter, IPR005828 : General substrate transporter, IPR016196 : Major facilitator superfamily, general substrate transporter |
| MGG00633.6 | 0.41 | potassium hydrogen antiporter | IPR006153 : Cation/H+ exchanger |
| MGG01256.6 | 0.41 | phosphoribosylaminoimidazole carboxylase | IPR000031 : 1-(5-Phosphoribosyl)-5-amino-4-imidazole-carboxylate (AIR) carboxylase, IPR003135 : ATP-grasp fold, ATP-dependent carboxylate-amine ligase-type, IPR013817 : Pre-ATP-grasp fold, |
| MGG01355.6 | 0.40 | conserved hypothetical protein | No defined Interpro term |
| MGG01272.6 | 0.40 | CORD and CS domain-containing protein | IPR007051 : CHORD, IPR007052 : CS domain, IPR008978 : HSP20-like chaperone IPR017447 : CS |
| MGG00212.6 | 0.40 | superoxide dismutase | IPR001189 : Manganese and iron superoxide dismutase |
| MGG06251.6 | 0.40 | conserved hypothetical protein | No defined Interpro term |
| MGG14581.6 | 0.40 | hypothetical protein | No defined Interpro term |
| MGG12809.6 | 0.40 | conserved hypothetical protein | IPR004087 : K Homology, IPR004088 : K Homology, type 1 |
| MGG05176.6 | 0.40 | mitochondrial outer membrane protein involved in mitochondrial shape | No defined Interpro term |
| MGG06428.6 | 0.40 | INSIG domain-containing protein | No defined Interpro term |
| MGG04182.6 | 0.40 | multidrug and toxin extrusion protein 1 | IPR002345 : Lipocalin, IPR002528 : Multi antimicrobial extrusion protein MatE |
| MGG03148.6 | 0.40 | not for RT-PCR conserved hypothetical protein | IPR005330 : MHYT |
| MGG05009.6 | 0.40 | canalicular multispecific organic anion transporter 2 | IPR001140 : ABC transporter, transmembrane region, IPR003439 : ABC transporter-like, IPR003593 : ATPase, AAA+ type, core, |
| MGG02979.6 | 0.40 | conserved hypothetical protein |  |
| MGG15428.6 | 0.40 | para-nitrobenzyl esterase | IPR006076 : FAD dependent oxidoreductase |
| MGG08904.6 | 0.40 | conserved hypothetical protein | IPR002076 : GNS1/SUR4 membrane protein |
| MGG12339.6 | 0.40 | conserved hypothetical protein | IPR001138 : Fungal transcriptional regulatory protein, N-terminal, IPR013055 : Tachykinin/Neurokinin like, conserved site |
| MGG00169.6 | 0.39 | conserved hypothetical protein | IPR003958 : Transcription factor CBF/NF-Y/archaeal histone, IPR009072 : Histone-fold |
| MGG06578.6 | 0.39 | hypothetical protein | No defined Interpro term |
| MGG08079.6 | 0.39 | rRNA processing protein Rrp17 | No defined Interpro term |
| MGG01246.6 | 0.39 | hypothetical protein | IPR003807 : Protein of unknown function DUF202 |
| MGG04084.6 | 0.39 | platelet-activating factor acetylhydrolase precursor | IPR005065 : Platelet-activating factor acetylhydrolase, plasma/intracellular isoform II |
| MGG06460.6 | 0.39 | conserved hypothetical protein | No defined Interpro term |
| MGG01830.6 | 0.39 | hypothetical protein | No defined Interpro term |
| MGG15428.6 | 0.39 | para-nitrobenzyl esterase | IPR002018 : Carboxylesterase, type B |
| MGG14757.6 | 0.39 | 1-phosphatidylinositol-3-phosphate 5-kinase FAB1 | IPR000306 : Zinc finger, FYVE-type, IPR002423 : Chaperonin Cpn60/TCP-1, IPR002498 : Phosphatidylinositol-4-phosphate 5-kinase, core, IPR013083 : Zinc finger, RING/FYVE/PHD-type, |
| MGG07317.6 | 0.39 | glutamate-cysteine ligase | IPR004308 : Glutamate-cysteine ligase catalytic subunit |
| MGG00951.6 | 0.39 | mannan endo-1,6-alpha-mannosidase DCW1 | IPR005198 : Glycoside hydrolase, family 76, IPR008928 : Six-hairpin glycosidase-like, IPR014480 : Mannan endo-1,6-alpha-mannosidase |
| MGG06766.6 | 0.39 | hypothetical protein | IPR001623 : Heat shock protein DnaJ, N-terminal, IPR015609 : Molecular chaperone, heat shock protein, Hsp40, DnaJ |
| MGG01401.6 | 0.39 | conserved hypothetical protein | No defined Interpro term |
| MGG00244.6 | 0.39 | 15-hydroxyprostaglandin dehydrogenase | IPR002198 : Short-chain dehydrogenase/reductase SDR, IPR002347 : Glucose/ribitol dehydrogenase, IPR016040 : NAD(P)-binding |
| MGG01311.6 | 0.39 | nuclear elongation and deformation protein 1 | IPR007651 : Lipin, N-terminal conserved region, IPR013209 : LNS2, Lipin/Ned1/Smp2 |
| MGG07751.6 | 0.38 | conserved hypothetical protein | No defined Interpro term |
| MGG02798.6 | 0.38 | short-chain dehydrogenase/reductase SDR | IPR002198 : Short-chain dehydrogenase/reductase SDR, IPR016040 : NAD(P)-binding |
| MGG14587.6 | 0.38 | ser/Thr protein phosphatase family protein | No defined Interpro term |
| MGG04897.6 | 0.38 | conserved hypothetical protein | IPR007114 : , IPR011701 : Major facilitator superfamily MFS-1, IPR016196 : Major facilitator superfamily, general substrate transporter |
| MGG07136.6 | 0.38 | nucleolar GTP-binding protein 1 | IPR005225 : Small GTP-binding protein, IPR006073 : GTP1/OBG, IPR012973 : NOG, C-terminal |
| MGG01872.6 | 0.38 | hypothetical protein | IPR008427 : Extracellular membrane protein, 8-cysteine region, CFEM |
| MGG14215.6 | 0.38 | pyridoxal-5'-phosphate-dependent protein beta subunit | IPR000277 : Cys/Met metabolism, pyridoxal phosphate-dependent enzyme, IPR001926 : Pyridoxal phosphate-dependent enzyme, beta subunit, IPR015424 : Pyridoxal phosphate-dependent transferase, major region, |
| MGG07518.6 | 0.38 | centromere/microtubule-binding protein cbf5 | IPR002478 : PUA, IPR004521 : Uncharacterized domain 2, IPR004802 : Putative rRNA pseudouridine synthase |
| MGG06905.6 | 0.38 | protein transport protein sec73 | IPR000904 : SEC7-like, IPR001849 : Pleckstrin homology, IPR011993 : Pleckstrin homology-type |
| MGG01820.6 | 0.38 | conserved hypothetical protein | No defined Interpro term |
| MGG05812.6 | 0.38 | phosphomevalonate kinase | IPR005916 : Phosphomevalonate kinase, eukaryotic, IPR006204 : GHMP kinase, IPR013750 : GHMP kinase, C-terminal |
| MGG11560.6 | 0.38 | taurine catabolism dioxygenase TauD | IPR003819 : Taurine catabolism dioxygenase TauD/TfdA |
| MGG10142.6 | 0.37 | hypothetical protein | No defined Interpro term |
| MGG00081.6 | 0.37 | conserved hypothetical protein | No defined Interpro term |
| MGG08781.6 | 0.37 | cupin domain-containing protein | IPR011051 : Cupin, RmlC-type, conserved barrel, IPR013096 : Cupin 2, conserved barrel, IPR014710 : RmlC-like jelly roll fold |
| MGG03629.6 | 0.37 | hypothetical protein | No defined Interpro term |
| MGG10484.6 | 0.37 | conserved hypothetical protein | No defined Interpro term |
| MGG07809.6 | 0.37 | exoglucanase 1 | IPR001722 : Glycoside hydrolase, family 7, IPR008985 : Concanavalin A-like lectin/glucanase |
| MGG14115.6 | 0.37 | GabA permease | IPR002293 : Amino acid/polyamine transporter I, IPR004841 : Amino acid permease-associated region |
| MGG12176.6 | 0.37 | conserved hypothetical protein | No defined Interpro term |
| MGG05525.6 | 0.37 | conserved hypothetical protein | IPR007913 : Uncharacterised protein family UPF0187 |
| MGG00302.6 | 0.37 | conserved hypothetical protein | IPR017943 : Bactericidal permeability-increasing protein, alpha/beta domain |
| MGG01312.6 | 0.37 | conserved hypothetical protein | IPR003006 : Immunoglobulin/major histocompatibility complex, conserved site |
| MGG03560.6 | 0.37 | phospholipid diacylglycerol acyltransferase | IPR003386 : Lecithin:cholesterol acyltransferase |
| MGG08768.6 | 0.37 | methyltransferase-UbiE family protein | IPR013216 : Methyltransferase type 11 |
| MGG09701.6 | 0.36 | conserved hypothetical protein | IPR007087 : Zinc finger, C2H2-type,IPR015880 : Zinc finger, C2H2-like |
| MGG08252.6 | 0.36 | conserved hypothetical protein | IPR006153 : Cation/H+ exchanger |
| MGG07010.6 | 0.36 | translation machinery-associated protein 46 | IPR000571 : Zinc finger, CCCH-type |
| MGG14633.6 | 0.36 | KpsF/GutQ family protein | IPR001199 : Cytochrome b5, IPR001347 : Sugar isomerase (SIS) |
| MGG11149.6 | 0.36 | conserved hypothetical protein | IPR005556 : SUN |
| MGG03298.6 | 0.36 | conserved hypothetical protein | IPR011701 : Major facilitator superfamily MFS-1, IPR016196 : Major facilitator superfamily, general substrate transporter |
| MGG06005.6 | 0.36 | RNA exonuclease 3 | IPR006055 : Exonuclease, IPR012337 : Polynucleotidyl transferase, Ribonuclease H fold, IPR013520 : Exonuclease, RNase T and DNA polymerase III |
| MGG08254.6 | 0.36 | conserved hypothetical protein | IPR005103 : Glycoside hydrolase, family 61 |
| MGG14691.6 | 0.36 | conserved hypothetical protein | IPR001680 : WD40 repeat, IPR011046 : WD40 repeat-like, IPR015943 : WD40/YVTN repeat-like |
| MGG00839.6 | 0.36 | vacuolar protein sorting-associated protein 74 | IPR008628 : Golgi phosphoprotein 3 |
| MGG06350.6 | 0.36 | hypothetical protein | IPR000637 : HMG-I and HMG-Y, DNA-binding, conserved site |
| MGG07474.6 | 0.36 | sreptomyces cyclase/dehydrase family protein | IPR005031 : Streptomyces cyclase/dehydrase |
| MGG07482.6 | 0.36 | conserved hypothetical protein | No defined Interpro term |
| MGG12383.6 | 0.36 | conserved hypothetical protein | No defined Interpro term |
| MGG11813.6 | 0.36 | hypothetical protein | No defined Interpro term |
| MGG04959.6 | 0.36 | hypothetical protein | No defined Interpro term |
| MGG01361.6 | 0.36 | PHO85 cyclin-1 | IPR006671 : Cyclin, N-terminal, IPR011028 : Cyclin-like, IPR012104 : Cyclin, fungal Pcl/HCS26 |
| MGG05343.6 | 0.35 | Transcription factor | IPR001138 : Fungal transcriptional regulatory protein, N-terminal, IPR007219 : Fungal specific transcription factor |
| MGG03418.6 | 0.35 | acyl-CoA dehydrogenase | IPR001199 : Cytochrome b5, IPR006090 : Acyl-CoA oxidase/dehydrogenase, type 1, IPR006091 : Acyl-CoA dehydrogenase/oxidase, central region |
| MGG03889.6 | 0.35 | threonine synthase | IPR001926 : Pyridoxal phosphate-dependent enzyme, beta subunit |
| MGG03711.6 | 0.35 | conserved hypothetical protein | IPR001138 : Fungal transcriptional regulatory protein, N-terminal |
| MGG02110.6 | 0.35 | caleosin domain-containing protein | IPR007736 : Caleosin related |
| MGG03384.6 | 0.35 | conserved hypothetical protein | No defined Interpro term |
| MGG00086.6 | 0.35 | 42 kDa endochitinase | IPR001223 : Glycoside hydrolase, family 18, catalytic domain, IPR001579 : Glycoside hydrolase, chitinase active site, ,IPR011583 : Chitinase II |
| MGG08486.6 | 0.35 | beta-lactamase family protein | IPR000871 : Beta-lactamase, class A/D, IPR001466 : Beta-lactamase-related, IPR012338 : Beta-lactamase-type transpeptidase fold |
| MGG07624.6 | 0.35 | hypothetical protein | No defined Interpro term |
| MGG05997.6 | 0.35 | hypothetical protein | No defined Interpro term |
| MGG06605.6 | 0.35 | DUF895 domain membrane protein | IPR010291 : Protein of unknown function DUF895, eukaryotic, IPR016196 : Major facilitator superfamily, general substrate transporter |
| MGG03892.6 | 0.34 | conserved hypothetical protein | No defined Interpro term |
| MGG07162.6 | 0.34 | sphingosine-1-phosphate lyase | IPR002129 : Pyridoxal phosphate-dependent decarboxylase, IPR015421 : Pyridoxal phosphate-dependent transferase, major region, subdomain 1, IPR015424 : Pyridoxal phosphate-dependent transferase, major region |
| MGG01376.6 | 0.34 | tyrosine-protein phosphatase 1 | IPR000242 : Protein-tyrosine phosphatase, receptor/non-receptor type, IPR000387 : Protein-tyrosine phosphatase, IPR001763 : Rhodanese-like |
| MGG02089.6 | 0.34 | hypothetical protein | No defined Interpro term |
| MGG01357.6 | 0.34 | conserved hypothetical protein | No defined Interpro term |
| MGG01956.6 | 0.34 | conserved hypothetical protein | No defined Interpro term |
| MGG15218.6 | 0.34 | hypothetical protein | No defined Interpro term |
| MGG09178.6 | 0.34 | conserved hypothetical protein | No defined Interpro term |
| MGG06868.6 | 0.34 | acetolactate synthase catalytic subunit |  |
| MGG01922.6 | 0.34 | polysaccharide deacetylase family protein | IPR002509 : Polysaccharide deacetylase, IPR011330 : Glycoside hydrolase/deacetylase, beta/alpha-barrel |
| MGG09762.6 | 0.34 | conserved hypothetical protein | IPR001547 : Glycoside hydrolase, family 5, IPR002035 : von Willebrand factor, type A |
| MGG15481.6 | 0.34 | conserved hypothetical protein | No defined Interpro term |
| MGG08272.6 | 0.34 | ATP-dependent RNA helicase dbp-3 | IPR000629 : RNA helicase, ATP-dependent, DEAD-box, conserved site, IPR001650 : DNA/RNA helicase, C-terminal, IPR011545 : DNA/RNA helicase, DEAD/DEAH box type, N-terminal |
| MGG14656.6 | 0.33 | phosphoglycerate mutase family protein | IPR001345 : Phosphoglycerate/bisphosphoglycerate mutase, IPR013078 : Phosphoglycerate mutase |
| MGG00994.6 | 0.33 | mannosyl-oligosaccharide 1,2-alpha-mannosidase IC | IPR001382 : Glycoside hydrolase, family 47 |
| MGG05356.6 | 0.33 | conserved hypothetical protein | No defined Interpro term |
| MGG08296.6 | 0.33 | hypothetical protein | No defined Interpro term |
| MGG06203.6 | 0.33 | high-affinity glucose transporter RGT2 | IPR003663 : Sugar/inositol transporter, IPR005828 : General substrate transporter, IPR016196 : Major facilitator superfamily, general substrate transporter |
| MGG00177.6 | 0.33 | conserved hypothetical protein | No defined Interpro term |
| MGG01057.6 | 0.33 | conserved hypothetical protein | No defined Interpro term |
| MGG13635.6 | 0.33 | hypothetical protein | IPR017441 : Protein kinase, ATP binding site |
| MGG12956.6 | 0.33 | conserved hypothetical protein | IPR001849 : Pleckstrin homology, IPR011993 : Pleckstrin homology-type, IPR012966 : Protein of unknown function DUF1709, fungi |
| MGG07473.6 | 0.33 | glycosyl hydrolase | IPR008985 : Concanavalin A-like lectin/glucanase, IPR013320 : Concanavalin A-like lectin/glucanase, subgroup |
| MGG09802.6 | 0.32 | conserved hypothetical protein | No defined Interpro term |
| MGG06116.6 | 0.32 | hypothetical protein | No defined Interpro term |
| MGG09115.6 | 0.32 | hypothetical protein | No defined Interpro term |
| MGG06534.6 | 0.32 | retinol dehydrogenase 12 | IPR002198 : Short-chain dehydrogenase/reductase SDR, IPR002347 : Glucose/ribitol dehydrogenase, IPR016040 : NAD(P)-binding |
| MGG08992.6 | 0.32 | hypothetical protein | No defined Interpro term |
| MGG11392.6 | 0.32 | conserved hypothetical protein | IPR006773 : Adhesion regulating molecule |
| MGG01752.6 | 0.32 | conserved hypothetical protein | IPR011011 : Zinc finger, FYVE/PHD-type |
| MGG01856.6 | 0.32 | conserved hypothetical protein | IPR006598 : Lipopolysaccharide-modifying protein |
| MGG15333.6 | 0.32 | conserved hypothetical protein | IPR001077 : O-methyltransferase, family 2, IPR016461 : O-methyltransferase, COMT, eukaryota |
| MGG01362.6 | 0.32 | cell division control protein 2 | IPR000719 : Protein kinase, core, IPR002290 : Serine/threonine protein kinase , IPR017442 : Serine/threonine protein kinase-related |
| MGG09985.6 | 0.32 | hypothetical protein | No defined Interpro term |
| MGG03007.6 | 0.32 | choline kinase | IPR002573 : Choline/ethanolamine kinase, IPR007521 : Choline kinase, N-terminal, IPR011009 : Protein kinase-like |
| MGG00636.6 | 0.32 | GPI mannosyltransferase 2 | IPR007315 : Mannosyltransferase, PIG-V |
| MGG00178.6 | 0.32 | hypothetical protein | IPR005579 : Cgr1 |
| MGG08428.6 | 0.32 | hypothetical protein | No defined Interpro term |
| MGG00979.6 | 0.32 | mitochondrial precursor proteins import receptor | IPR001440 : Tetratricopeptide TPR-1, IPR005687 : Mitochondrial import translocase, subunit Tom70, IPR011990 : Tetratricopeptide-like helical |
| MGG00145.6 | 0.32 | RING-1 protein | IPR006845 : Pex, N-terminal |
| MGG12358.6 | 0.31 | conserved hypothetical protein | IPR014848 : Rgp1 |
| MGG09462.6 | 0.31 | inositol oxygenase 1 | IPR007828 : Protein of unknown function DUF706 |
| MGG00905.6 | 0.31 | Sec14 cytosolic factor | IPR001071 : Cellular retinaldehyde binding/alpha-tocopherol transport, IPR001251 : Cellular retinaldehyde-binding/triple function, C-terminal, IPR008273 : Cellular retinaldehyde-binding/triple function, N-terminal |
| MGG00976.6 | 0.31 | ATP-dependent helicase NAM7 | IPR014001 : DEAD-like helicase, N-terminal |
| MGG01916.6 | 0.31 | hypothetical protein | No defined Interpro term |
| MGG08487.6 | 0.31 | cellobiose dehydrogenase | IPR000172 : Glucose-methanol-choline oxidoreductase, N-terminal, IPR007867 : Glucose-methanol-choline oxidoreductase, C-terminal, IPR013027 : FAD-dependent pyridine nucleotide-disulphide oxidoreductase |
| MGG01115.6 | 0.31 | ubiquitin-protein ligase E3A | IPR000569 : HECT |
| MGG03087.6 | 0.31 | cell division control protein 11 | IPR000038 : Cell division/GTP binding protein, IPR016491 : Septin |
| MGG05987.6 | 0.30 | hypothetical protein | IPR002125 : CMP/dCMP deaminase, zinc-binding |
| MGG14151.6 | 0.30 | conserved hypothetical protein | No defined Interpro term |
| MGG00940.6 | 0.30 | tripeptidyl-peptidase 1 precursor | IPR000209 : Peptidase S8 and S53, subtilisin, kexin, sedolisin, IPR009020 : Proteinase inhibitor, propeptide,I IPR015366 : Peptidase S53, propeptide |
| MGG13156.6 | 0.30 | conserved hypothetical protein |  |
| MGG12832.6 | 0.30 | sorting nexin-41 | IPR001683 : Phox-like |
| MGG00721.6 | 0.30 | conserved hypothetical protein | IPR002821 : Hydantoinase/oxoprolinase, IPR008040 : Hydantoinaseoxoprolinase, N-terminal, IPR010318 : Protein of unknown function DUF917 |
| MGG10497.6 | 0.30 | blue light-inducible protein Bli-3 | IPR009002 : FMN-binding split barrel, related, IPR011576 : Pyridoxamine 5'-phosphate oxidase-related, FMN-binding core |
| MGG15143.6 | 0.30 | hypothetical protein | No defined Interpro term |
| MGG09821.6 | 0.30 | conserved hypothetical protein | IPR011701 : Major facilitator superfamily MFS-1, IPR016196 : Major facilitator superfamily, general substrate transporter |
| MGG14639.6 | 0.30 | hypothetical protein | No defined Interpro term |
| MGG12521.6 | 0.30 | hypothetical protein | No defined Interpro term |
| MGG06144.6 | 0.29 | hypothetical protein | No defined Interpro term |
| MGG11468.6 | 0.29 | isoflavone reductase | IPR008030 : NmrA-like, IPR016040 : NAD(P)-binding |
| MGG11026.6 | 0.29 | conserved hypothetical protein | No defined Interpro term |
| MGG13156.6 | 0.29 | conserved hypothetical protein | IPR007087 : Zinc finger, C2H2-type, IPR013087 : Zinc finger, C2H2-type/integrase, DNA-binding |
| MGG09845.6 | 0.29 | conserved hypothetical protein | IPR008960 : Carbohydrate-binding family 9/cellobiose dehydrogenase, cytochrome, IPR015920 : Cellobiose dehydrogenase, cytochrome |
| MGG00359.6 | 0.29 | Delta(3,5)-Delta(2,4)-dienoyl-CoA isomerase | IPR001753 : Crotonase, core |
| MGG05318.6 | 0.29 | conserved hypothetical protein | IPR013925 : Spindle pole body interacting protein |
| MGG13448.6 | 0.29 | DUF967 domain-containing protein | IPR005624 : Protein of unknown function DUF336, IPR010371 : Protein of unknown function DUF967 |
| MGG03067.6 | 0.29 | hypothetical protein | No defined Interpro term |
| MGG03041.6 | 0.29 | glucokinase | IPR001312 : Hexokinase |
| MGG08110.6 | 0.28 | hypothetical protein | No defined Interpro term |
| MGG06926.6 | 0.28 | cyclopropane-fatty-acyl-phospholipid synthase | IPR013216 : Methyltransferase type 11 11 |
| MGG07245.6 | 0.28 | hypothetical protein | No defined Interpro term |
| MGG02614.6 | 0.28 | hypothetical protein | IPR011058 : Cyanovirin-N |
| MGG04956.6 | 0.28 | long-chain-fatty-acid-CoA ligase 1 | IPR000873 : AMP-dependent synthetase and ligase |
| MGG08732.6 | 0.28 | interferon-induced GTP-binding protein Mx | IPR001401 : Dynamin, GTPase region |
| MGG10551.6 | 0.28 | hypothetical protein | No defined Interpro term |
| MGG11534.6 | 0.28 | conserved hypothetical protein | IPR001092 : Basic helix-loop-helix dimerisation region bHLH, IPR011598 : Helix-loop-helix DNA-binding |
| MGG10170.6 | 0.28 | conserved hypothetical protein | IPR010721 : Protein of unknown function DUF1295 |
| MGG13764.6 | 0.28 | bilirubin oxidase | IPR008972 : Cupredoxin, IPR011706 : Multicopper oxidase, type 2, IPR011707 : Multicopper oxidase, type 3 |
| MGG04663.6 | 0.28 | binding / zinc ion binding | IPR001841 : Zinc finger, RING-type, IPR017907 : Zinc finger, RING-type, conserved site |
| MGG05120.6 | 0.28 | hypothetical protein | No defined Interpro term |
| MGG05573.6 | 0.27 | conserved hypothetical protein | No defined Interpro term |
| MGG00882.6 | 0.27 | negative regulator of the PHO system | IPR013781 : Glycoside hydrolase, subgroup, catalytic core, IPR017853 : Glycoside hydrolase, catalytic core |
| MGG06898.6 | 0.27 | Transcription factor | IPR001005 : SANT, DNA-binding, IPR009057 : Homeodomain-like, IPR012287 : Homeodomain-related, IPR014778 : Myb, DNA-binding, IPR015495 : Myb transcription factor |
| MGG11632.6 | 0.27 | hypothetical protein | No defined Interpro term |
| MGG09428.6 | 0.27 | conserved hypothetical protein | No defined Interpro term |
| MGG14662.6 | 0.27 | hypothetical protein | No defined Interpro term |
| MGG06807.6 | 0.27 | conserved hypothetical protein | No defined Interpro term |
| MGG05240.6 | 0.27 | MYB DNA-binding domain-containing protein | IPR001005 : SANT, DNA-binding, IPR012287 : Homeodomain-related, IPR014778 : Myb, DNA-binding |
| MGG03448.6 | 0.26 | golgi transport complex component Cog5 | No defined Interpro term |
| MGG03205.6 | 0.26 | polyamine transporter 2 | IPR007114 : , IPR011701 : Major facilitator superfamily MFS-1, IPR016196 : Major facilitator superfamily, general substrate transporter |
| MGG05518.6 | 0.26 | hypothetical protein | IPR007087 : Zinc finger, C2H2-type,IPR007087 : Zinc finger, C2H2-type, IPR015880 : Zinc finger, C2H2-like,IPR015880 : Zinc finger, C2H2-like |
| MGG02088.6 | 0.26 | conserved hypothetical protein | IPR013922 : Cyclin-related 2 |
| MGG00725.6 | 0.26 | conserved hypothetical protein | No defined Interpro term |
| MGG00965.6 | 0.26 | MSF1 domain-containing protein | IPR006797 : PRELI/MSF1 |
| MGG03556.6 | 0.26 | H/ACA ribonucleoprotein complex subunit 1 | IPR007504 : Gar1 protein RNA-binding region |
| MGG04171.6 | 0.26 | hypothetical protein | No defined Interpro term |
| MGG07519.6 | 0.26 | hypothetical protein | No defined Interpro term |
| MGG10736.6 | 0.26 | hypothetical protein | No defined Interpro term |
| MGG08193.6 | 0.25 | hypothetical protein | IPR013838 : Beta tubulin, autoregulation binding site |
| MGG03475.6 | 0.25 | hypothetical protein | IPR001304 : C-type lectin |
| MGG12322.6 | 0.25 | DUF907 domain protein | IPR010308 : Protein of unknown function DUF907, fungi, fungi, IPR010916 : TonB box, conserved site |
| MGG03427.6 | 0.25 | conserved hypothetical protein | IPR007114 : , IPR011701 : Major facilitator superfamily MFS-1, IPR016196 : Major facilitator superfamily, general substrate transporter |
| MGG07053.6 | 0.25 | conserved hypothetical protein | IPR005654 : ATPase, AFG1-like |
| MGG02641.6 | 0.25 | conserved hypothetical protein | No defined Interpro term |
| MGG00607.6 | 0.25 | conserved hypothetical protein | IPR002575 : Aminoglycoside phosphotransferase IPR011009 : Protein kinase-like |
| MGG08111.6 | 0.25 | conserved hypothetical protein | No defined Interpro term |
| MGG03705.6 | 0.25 | SH3 domain-containing protein | IPR001452 : Src homology-3 domain |
| MGG14661.6 | 0.25 | hypothetical protein | No defined Interpro term |
| MGG07551.6 | 0.25 | cytochrome P450 monooxygenase | IPR001128 : Cytochrome P450, IPR002403 : Cytochrome P450, E-class, group IV |
| MGG08173.6 | 0.25 | NADP-dependent malic enzyme | IPR012301 : Malic enzyme, N-terminal, IPR012302 : Malic enzyme, NAD-binding, IPR016040 : NAD(P)-binding |
| MGG14716.6 | 0.24 | hypothetical protein | No defined Interpro term |
| MGG14792.6 | 0.24 | hypothetical protein | No defined Interpro term |
| MGG01844.6 | 0.24 | GPI inositol-deacylase | IPR012908 : PGAP1-like |
| MGG00655.6 | 0.24 | peroxisomal biogenesis factor 2 | IPR001841 : Zinc finger, RING-type, IPR006845 : Pex, N-terminal, IPR013083 : Zinc finger, RING/FYVE/PHD-type |
| MGG06877.6 | 0.24 | DUF618 domain-containing protein | IPR006569 : Regulation of nuclear pre-mRNA protein, IPR006903 : Protein of unknown function DUF618, IPR008942 : ENTH/VHS |
| MGG01944.6 | 0.24 | hypothetical protein | No defined Interpro term |
| MGG06965.6 | 0.24 | conserved hypothetical protein | IPR000504 : RNA recognition motif, RNP-1 |
| MGG09402.6 | 0.23 | conserved hypothetical protein | No defined Interpro term |
| MGG05101.6 | 0.23 | conserved hypothetical protein | IPR001509 : NAD-dependent epimerase/dehydratase, IPR016040 : NAD(P)-binding |
| MGG03369.6 | 0.23 | conserved hypothetical protein | IPR011038 : Calycin-like, IPR012674 : Calycin,IPR012674 : Calycin, IPR013208 : Lipocalin-like |
| MGG00912.6 | 0.23 | protein-tyrosine phosphatase 2 | IPR000242 : Protein-tyrosine phosphatase, receptor/non-receptor type, IPR000387 : Protein-tyrosine phosphatase, IPR016130 : Protein-tyrosine phosphatase, active site |
| MGG01532.6 | 0.23 | hypothetical protein | No defined Interpro term |
| MGG12345.6 | 0.23 | conserved hypothetical protein | IPR001619 : Sec1-like protein |
| MGG02339.6 | 0.23 | hypothetical protein | IPR011058 : Cyanovirin-N |
| MGG12005.6 | 0.23 | cation-transporting ATPase 4 | IPR001757 : ATPase, P-type, K/Mg/Cd/Cu/Zn/Na/Ca/Na/H-transporter, IPR005834 : Haloacid dehalogenase-like hydrolase, IPR006544 : ATPase, P-type, unknown pump specificity (type V) |
| MGG01324.6 | 0.23 | urease | IPR002019 : Urease, beta subunit, IPR002026 : Urease, gamma subunit region, IPR005848 : Urease, alpha subunit |
| MGG04353.6 | 0.23 | hypothetical protein | No defined Interpro term |
| MGG05670.6 | 0.23 | conserved hypothetical protein |  |
| MGG08024.6 | 0.22 | hypothetical protein | No defined Interpro term |
| MGG07207.6 | 0.22 | D-3-phosphoglycerate dehydrogenase | IPR002912 : Amino acid-binding ACT, IPR006139 : D-isomer specific 2-hydroxyacid dehydrogenase, catalytic region, IPR006140 : D-isomer specific 2-hydroxyacid dehydrogenase, NAD-binding, |
| MGG08255.6 | 0.22 | conserved hypothetical protein | IPR000210 : BTB/POZ-like, IPR006652 : Kelch repeat type 1, IPR011043 : Galactose oxidase/kelch, beta-propeller |
| MGG10606.6 | 0.22 | aminopeptidase 2 | IPR001930 : Peptidase M1, membrane alanine aminopeptidase, IPR014782 : Peptidase M1, membrane alanine aminopeptidase, N-terminal |
| MGG05216.6 | 0.22 | conserved hypothetical protein | IPR000195 : RabGAP/TBC |
| MGG09533.6 | 0.22 | hypothetical protein | No defined Interpro term |
| MGG02801.6 | 0.22 | conserved hypothetical protein | No defined Interpro term |
| MGG03238.6 | 0.22 | zinc finger protein ZPR1 | IPR004457 : Zinc finger, ZPR1-type |
| MGG05763.6 | 0.21 | stress responsive A/B barrel domain-containing protein | IPR011008 : Dimeric alpha-beta barrel, IPR013097 : Stress responsive alpha-beta barrel |
| MGG12973.6 | 0.21 | conserved hypothetical protein | IPR012469 : Protein of unknown function DUF1688, IPR012469 : Protein of unknown function DUF1688 |
| MGG04575.6 | 0.21 | conserved hypothetical protein | No defined Interpro term |
| MGG09545.6 | 0.21 | conserved hypothetical protein | IPR006043 : Xanthine/uracil/vitamin C permease |
| MGG14006.6 | 0.21 | hypothetical protein | No defined Interpro term |
| MGG00733.6 | 0.21 | conserved hypothetical protein | No defined Interpro term |
| MGG12598.6 | 0.21 | pantothenate kinase | IPR004567 : Eukaryotic pantothenate kinase, IPR011602 : Fumble |
| MGG11599.6 | 0.20 | endoplasmic reticulum mannosyl-oligosaccharide 1,2-alpha-mannosidase | IPR001382 : Glycoside hydrolase, family 47 |
| MGG13405.6 | 0.20 | geranylgeranyl pyrophosphate synthetase | IPR000092 : Polyprenyl synthetase, IPR008949 : Terpenoid synthase, IPR017446 : Polyprenyl synthetase-related |
| MGG04880.6 | 0.20 | conserved hypothetical protein | No defined Interpro term |
| MGG07607.6 | 0.20 | conserved hypothetical protein | No defined Interpro term |
| MGG04662.6 | 0.20 | conserved hypothetical protein | IPR001993 : Mitochondrial substrate carrier |
| MGG08547.6 | 0.20 | serine/threonine-protein kinase srk1 | IPR000719 : Protein kinase, core, IPR002290 : Serine/threonine protein kinase, IPR008271 : Serine/threonine protein kinase, active site |
| MGG01996.6 | 0.20 | conserved hypothetical protein | No defined Interpro term |
| MGG09171.6 | 0.20 | tetracycline resistance protein from transposon | IPR006076 : FAD dependent oxidoreductase |
| MGG09071.6 | 0.20 | aminobenzoyl-glutamate utilization protein B | IPR002933 : Peptidase M20, IPR010168 : Peptidase M20D, amidohydrolase, IPR011650 : Peptidase M20, dimerisation |
| MGG07327.6 | 0.20 | asparagine-rich protein | IPR000504 : RNA recognition motif, RNP-1, IPR001876 : Zinc finger, RanBP2-type, IPR012677 : Nucleotide-binding, alpha-beta plait |
| MGG01883.6 | 0.19 | conserved hypothetical protein | IPR007114 : , IPR011701 : Major facilitator superfamily MFS-1, IPR016196 : Major facilitator superfamily, general substrate transporter |
| MGG14655.6 | 0.19 | hypothetical protein | No defined Interpro term |
| MGG07192.6 | 0.19 | conserved hypothetical protein | IPR000169 : Peptidase, cysteine peptidase active site, IPR002332 : Nitrogen regulatory protein P-II, urydylation site, IPR007603 : Protein of unknown function DUF580 |
| MGG00960.6 | 0.19 | phospholipase D1 | IPR001683 : Phox-like, IPR001736 : Phospholipase D/Transphosphatidylase, IPR015679 : Phospholipase D |
| MGG02890.6 | 0.19 | conserved hypothetical protein | No defined Interpro term |
| MGG13736.6 | 0.19 | hypothetical protein | No defined Interpro term |
| MGG03694.6 | 0.19 | beclin-1 | IPR007243 : Autophagy protein 6 |
| MGG04925.6 | 0.19 | hypothetical protein | No defined Interpro term |
| MGG09055.6 | 0.19 | hypothetical protein | No defined Interpro term |
| MGG14825.6 | 0.19 | hypothetical protein | No defined Interpro term |
| MGG00332.6 | 0.19 | conserved hypothetical protein | IPR012479 : HCNGP-like |
| MGG06242.6 | 0.19 | ribosome biogenesis protein Kri1 | IPR007851 : KRR1 interacting protein 1, subgroup |
| MGG00800.6 | 0.19 | dual specificity protein kinase FUZ7 | IPR000719 : Protein kinase, core, IPR001245 : Tyrosine protein kinase, IPR002290 : Serine/threonine protein kinase |
| MGG09170.6 | 0.18 | conserved hypothetical protein | IPR009604 : LsmAD domain |
| MGG00632.6 | 0.18 | cell differentiation protein rcd1 | IPR007216 : Cell differentiation, Rcd1-like, IPR016024 : Armadillo-type fold |
| MGG02143.6 | 0.18 | hypothetical protein | No defined Interpro term |
| MGG02775.6 | 0.18 | conserved hypothetical protein | IPR007087 : Zinc finger, C2H2-type, IPR015880 : Zinc finger, C2H2-like |
| MGG05295.6 | 0.18 | conserved hypothetical protein | No defined Interpro term |
| MGG05146.6 | 0.18 | ribosomal RNA-processing protein 1 | IPR010301 : Nucleolar, Nop52 |
| MGG02350.6 | 0.18 | conserved hypothetical protein | IPR005123 : 2OG-Fe(II) oxygenase |
| MGG15403.6 | 0.18 | acetylxylan esterase 2 | IPR000675 : Cutinase |
| MGG14700.6 | 0.18 | conserved hypothetical protein | No defined Interpro term |
| MGG04165.6 | 0.18 | conserved hypothetical protein | IPR003864 : Protein of unknown function DUF221 |
| MGG01102.6 | 0.18 | ornithine carbamoyltransferase | IPR002292 : Ornithine carbamoyltransferase, IPR006130 : Aspartate/ornithine carbamoyltransferase, IPR006131 : Aspartate/ornithine carbamoyltransferase, Asp/Orn-binding region, I |
| MGG04873.6 | 0.18 | conserved hypothetical protein | No defined Interpro term |
| MGG01392.6 | 0.18 | hypothetical protein | IPR006094 : FAD linked oxidase, N-terminal, IPR016166 : FAD-binding, type 2 |
| MGG03597.6 | 0.17 | conserved hypothetical protein |  |
| MGG02413.6 | 0.17 | hypothetical protein | No defined Interpro term |
| MGG03482.6 | 0.17 | hypothetical protein | No defined Interpro term |
| MGG15250.6 | 0.17 | conserved hypothetical protein | IPR000760 : Inositol monophosphatase |
| MGG05574.6 | 0.17 | conserved hypothetical protein | IPR006076 : FAD dependent oxidoreductase |
| MGG09356.6 | 0.17 | hypothetical protein | No defined Interpro term |
| MGG08122.6 | 0.17 | DNA replication licensing factor mcm2 | IPR001208 : DNA-dependent ATPase MCM, IPR008045 : MCM protein 2, IPR016027 : Nucleic acid-binding, OB-fold-like |
| MGG11192.6 | 0.17 | hypothetical protein | IPR001138 : Fungal transcriptional regulatory protein, N-terminal |
| MGG03843.6 | 0.17 | conserved hypothetical protein | IPR007114 : , IPR011701 : Major facilitator superfamily MFS-1, IPR016196 : Major facilitator superfamily, general substrate transporter |
| MGG06303.6 | 0.17 | conserved hypothetical protein | IPR013094 : Alpha/beta hydrolase fold-3 |
| MGG01586.6 | 0.17 | conserved hypothetical protein | IPR007087 : Zinc finger, C2H2-type |
| MGG11378.6 | 0.17 | hypothetical protein | No defined Interpro term |
| MGG04835.6 | 0.16 | conserved hypothetical protein | No defined Interpro term |
| MGG08926.6 | 0.16 | conserved hypothetical protein | No defined Interpro term |
| MGG07257.6 | 0.16 | hypothetical protein | No defined Interpro term |
| MGG04191.6 | 0.16 | heat shock protein SSC1 | IPR001023 : Heat shock protein Hsp70, IPR012725 : Chaperone DnaK,IPR013126 : Heat shock protein 70 |
| MGG05761.6 | 0.16 | conserved hypothetical protein | No defined Interpro term |
| MGG02766.6 | 0.16 | succinate-semialdehyde dehydrogenase | IPR015590 : Aldehyde dehydrogenase, IPR016160 : Aldehyde dehydrogenase, conserved site, IPR016161 : Aldehyde/histidinol dehydrogenase |
| MGG06553.6 | 0.16 | retinol dehydrogenase 13 | IPR002198 : Short-chain dehydrogenase/reductase SDR, IPR016040 : NAD(P)-binding |
| MGG03336.6 | 0.16 | LEA domain-containing protein | No defined Interpro term |
| MGG08917.6 | 0.16 | conserved hypothetical protein | IPR001138 : Fungal transcriptional regulatory protein, N-terminal |
| MGG01016.6 | 0.16 | chaperone protein dnaJ 6 | IPR001623 : Heat shock protein DnaJ, N-terminal, IPR015609 : Molecular chaperone, heat shock protein, Hsp40, DnaJ |
| MGG05592.6 | 0.16 | conserved hypothetical protein | IPR011118 : Tannase and feruloyl esterase |
| MGG10277.6 | 0.16 | brefeldin A resistance protein | IPR003439 : ABC transporter-like, IPR003593 : ATPase, AAA+ type, core, IPR010929 : CDR ABC transporter |
| MGG09970.6 | 0.16 | hypothetical protein | No defined Interpro term |
| MGG06323.6 | 0.16 | solute carrier family 35 member E3 | IPR004853 : Protein of unknown function DUF250 |
| MGG08941.6 | 0.16 | hypothetical protein | IPR011058 : Cyanovirin-N |
| MGG00455.6 | 0.16 | class E vacuolar protein-sorting machinery protein hse-1 | IPR001452 : Src homology-3 domain, IPR003903 : Ubiquitin interacting motif, IPR008942 : ENTH/VHS |
| MGG02118.6 | 0.16 | conserved hypothetical protein | IPR001214 : SET |
| MGG00504.6 | 0.16 | Transcription factor | IPR007087 : Zinc finger, C2H2-type, IPR013087 : Zinc finger, C2H2-type/integrase, DNA-binding, IPR015880 : Zinc finger, C2H2-like |
| MGG15351.6 | 0.15 | hypothetical protein | IPR006109 : NAD-dependent glycerol-3-phosphate dehydrogenase, C-terminal, IPR006168 : NAD-dependent glycerol-3-phosphate dehydrogenase, IPR008927 : 6-phosphogluconate dehydrogenase, C-terminal-like |
| MGG04487.6 | 0.15 | conserved hypothetical protein | No defined Interpro term |
| MGG14956.6 | 0.15 | NADH-cytochrome b5 reductase 2 | IPR001199 : Cytochrome b5, IPR001433 : Oxidoreductase FAD/NAD(P)-binding, IPR017927 : Ferredoxin reductase-type FAD-binding domain |
| MGG06832.6 | 0.15 | conserved hypothetical protein | IPR001138 : Fungal transcriptional regulatory protein, N-terminal |
| MGG06572.6 | 0.15 | phosphatidylinositol-4-phosphate 5-kinase its3 (PtdIns(4)P-5-kinase) | IPR002498 : Phosphatidylinositol-4-phosphate 5-kinase, core |
| MGG08659.6 | 0.15 | hypothetical protein | No defined Interpro term |
| MGG07198.6 | 0.15 | conserved hypothetical protein | IPR001680 : WD40 repeat, IPR011046 : WD40 repeat-like, IPR015943 : WD40/YVTN repeat-like |
| MGG05327.6 | 0.15 | hypothetical protein | No defined Interpro term |
| MGG06225.6 | 0.15 | hypothetical protein | No defined Interpro term |
| MGG01933.6 | 0.15 | conserved hypothetical protein | No defined Interpro term |
| MGG00916.6 | 0.15 | hypothetical protein | IPR007087 : Zinc finger, C2H2-type, IPR015880 : Zinc finger, C2H2-like |
| MGG02188.6 | 0.15 | glutamyl-tRNA(Gln) amidotransferase subunit A | IPR000120 : Amidase signature enzyme |
| MGG10214.6 | 0.15 | fumarylacetoacetate hydrolase domain-containing protein 2 | IPR002529 : Fumarylacetoacetase, C-terminal-like, IPR011234 : Fumarylacetoacetase, C-terminal-related |
| MGG00371.6 | 0.15 | cell division control protein 25 | IPR000651 : Guanine nucleotide exchange factor for Ras-like GTPases, N-terminal, IPR001452 : Src homology-3 domain, IPR008937 : Ras guanine nucleotide exchange factor |
| MGG09280.6 | 0.15 | hypothetical protein | No defined Interpro term |
| MGG01145.6 | 0.15 | hypothetical protein | No defined Interpro term |
| MGG10710.6 | 0.14 | oxidoreductase | IPR003042 : Aromatic-ring hydroxylase-like, IPR006076 : FAD dependent oxidoreductase |
| MGG12988.6 | 0.14 | alpha-glucoside transport protein | IPR003663 : Sugar/inositol transporter, IPR005828 : General substrate transporter, IPR016196 : Major facilitator superfamily, general substrate transporter |
| MGG01391.6 | 0.14 | ent-kaurene oxidase | IPR001128 : Cytochrome P450, IPR002403 : Cytochrome P450, E-class, group IV |
| MGG02109.6 | 0.14 | conserved hypothetical protein | No defined Interpro term |
| MGG12552.6 | 0.14 | hypothetical protein | No defined Interpro term |
| MGG07517.6 | 0.14 | conserved hypothetical protein | IPR000700 : PAS-associated, C-terminal |
| MGG00635.6 | 0.14 | conserved hypothetical protein | No defined Interpro term |
| MGG07311.6 | 0.14 | hypothetical protein | No defined Interpro term |
| MGG07848.6 | 0.14 | multidrug resistance protein CDR1 | IPR003439 : ABC transporter-like, IPR003593 : ATPase, AAA+ type, core, IPR010929 : CDR ABC transporter |
| MGG05100.6 | 0.13 | hypothetical protein | IPR001283 : Allergen V5/Tpx-1 related, IPR014044 : SCP-like extracellular |
| MGG03896.6 | 0.13 | conserved hypothetical protein | No defined Interpro term |
| MGG02612.6 | 0.13 | 3-oxoacyl-[acyl-carrier-protein] reductase | IPR002198 : Short-chain dehydrogenase/reductase SDR, IPR016040 : NAD(P)-binding |
| MGG08161.6 | 0.13 | conserved hypothetical protein | IPR013217 : Methyltransferase type 12 |
| MGG10006.6 | 0.13 | hypothetical protein | No defined Interpro term |
| MGG08846.6 | 0.13 | conserved hypothetical protein | IPR000759 : Adrenodoxin reductase, IPR013027 : FAD-dependent pyridine nucleotide-disulphide oxidoreductase |
| MGG12421.6 | 0.12 | aminomethyltransferase | IPR006076 : FAD dependent oxidoreductase, IPR006222 : Glycine cleavage T-protein, N-terminal, IPR013977 : Glycine cleavage T-protein, C-terminal barrel |
| MGG04378.6 | 0.12 | integral membrane protein | No defined Interpro term |
| MGG04346.6 | 0.12 | sterol 24-C-methyltransferase | IPR013216 : Methyltransferase type 11, IPR013705 : Sterol methyltransferase C-terminal |
| MGG02817.6 | 0.12 | glutamate decarboxylase | IPR000760 : Inositol monophosphatase, IPR002129 : Pyridoxal phosphate-dependent decarboxylase, IPR010107 : Glutamate decarboxylase |
| MGG02294.6 | 0.12 | ent-kaurene oxidase | IPR001128 : Cytochrome P450 |
| MGG13014.6 | 0.12 | chitin synthase | IPR001199 : Cytochrome b5, IPR001609 : Myosin head, motor region, IPR002355 : Multicopper oxidase, copper-binding site |
| MGG05673.6 | 0.12 | 40S ribosomal protein S3 | IPR001351 : Ribosomal protein S3, C-terminal, IPR004044 : K Homology, type 2, PR005703 : Ribosomal protein S3, eukaryotic/archaeal |
| MGG07358.6 | 0.12 | serin endopeptidase | IPR000209 : Peptidase S8 and S53, subtilisin, kexin, sedolisin, IPR003137 : Protease-associated PA, IPR015500 : Peptidase S8, subtilisin-related |
| MGG00334.6 | 0.12 | hypothetical protein | No defined Interpro term |
| MGG13332.6 | 0.11 | aquaporin | IPR000425 : Major intrinsic protein |
| MGG02187.6 | 0.11 | hypothetical protein | No defined Interpro term |
| MGG11816.6 | 0.11 | NADPH-dependent 1-acyldihydroxyacetone phosphate reductase | IPR002198 : Short-chain dehydrogenase/reductase SDR, IPR002347 : Glucose/ribitol dehydrogenase, IPR016040 : NAD(P)-binding |
| MGG04699.6 | 0.11 | C2H2 finger domain-containing protein | IPR007087 : Zinc finger, C2H2-type, IPR013087 : Zinc finger, C2H2-type/integrase, DNA-binding, IPR015880 : Zinc finger, C2H2-like |
| MGG02942.6 | 0.11 | conserved hypothetical protein | No defined Interpro term |
| MGG01894.6 | 0.10 | glycerol-3-phosphate acyltransferase | IPR002123 : Phospholipid/glycerol acyltransferase |
| MGG08291.6 | 0.10 | conserved hypothetical protein | IPR008972 : Cupredoxin |
| MGG13926.6 | 0.10 | conserved hypothetical protein | IPR016137 : Regulator of G protein signalling superfamily |
| MGG03973.6 | 0.10 | splicing factor 3B subunit 4 | IPR000504 : RNA recognition motif, RNP-1, IPR012677 : Nucleotide-binding, alpha-beta plait |
| MGG02329.6 | 0.10 | isotrichodermin C-15 hydroxylase | IPR001128 : Cytochrome P450, IPR001209 : Ribosomal protein S14, IPR002401 : Cytochrome P450, E-class, group I |
| MGG08429.6 | 0.10 | serin endopeptidas | IPR000209 : Peptidase S8 and S53, subtilisin, kexin, sedolisin, IPR003137 : Protease-associated PA, IPR015500 : Peptidase S8, subtilisin-related |
| MGG13535.6 | 0.10 | nucleoporin POM152 | No defined Interpro term |
| MGG07150.6 | 0.10 | conserved hypothetical protein | No defined Interpro term |
| MGG05584.6 | 0.10 | conserved hypothetical protein | IPR004854 : Ubiquitin fusion degradation protein UFD1 |
| MGG10571.6 | 0.10 | conserved hypothetical protein | No defined Interpro term |
| MGG15343.6 | 0.10 | hypothetical protein | No defined Interpro term |
| MGG00681.6 | 0.10 | conserved hypothetical protein | IPR013217 : Methyltransferase type 12 |
| MGG01485.6 | 0.09 | conserved hypothetical protein | IPR011701 : Major facilitator superfamily MFS-1, IPR016196 : Major facilitator superfamily, general substrate transporter |
| MGG12146.6 | 0.09 | ER membrane protein | IPR013635 : ICE2 |
| MGG03403.6 | 0.09 | hypothetical protein | No defined Interpro term |
| MGG11408.6 | 0.09 | endoplasmic reticulum mannosyl-oligosaccharide 1,2-alpha-mannosidase | IPR001382 : Glycoside hydrolase, family 47 |
| MGG02962.6 | 0.09 | C6 zinc finger domain-containing protein | IPR001138 : Fungal transcriptional regulatory protein, N-terminal |
| MGG00748.6 | 0.09 | myosin-5 | IPR000048 : IQ calmodulin-binding region, IPR001452 : Src homology-3 domain, IPR001609 : Myosin head, motor region, IPR010926 : Myosin tail 2 |
| MGG04411.6 | 0.08 | conserved hypothetical protein | No defined Interpro term |
| MGG02837.6 | 0.08 | conserved hypothetical protein | No defined Interpro term |
| MGG10107.6 | 0.08 | caleosin domain-containing protein | IPR007736 : Caleosin related |
| MGG05109.6 | 0.08 | conserved hypothetical protein | No defined Interpro term |
| MGG05813.6 | 0.08 | hypothetical protein | No defined Interpro term |
| MGG00438.6 | 0.08 | conserved hypothetical protein | IPR004345 : TB2/DP1 and HVA22 related protein |
| MGG05035.6 | 0.08 | hypothetical protein | No defined Interpro term |
| MGG12522.6 | 0.08 | hypothetical protein | No defined Interpro term |
| MGG00739.6 | 0.08 | conserved hypothetical protein | IPR004871 : Cleavage and polyadenylation specificity factor, A subunit, C-terminal |
| MGG15378.6 | 0.07 | hypothetical protein | No defined Interpro term |
| MGG01273.6 | 0.07 | conserved hypothetical protein | No defined Interpro term |
| MGG02840.6 | 0.07 | conserved hypothetical protein | IPR011701 : Major facilitator superfamily MFS-1, IPR016196 : Major facilitator superfamily, general substrate transporter |
| MGG03871.6 | 0.07 | hypothetical protein | No defined Interpro term |
| MGG05805.6 | 0.07 | hypothetical protein | No defined Interpro term |
| MGG03634.6 | 0.07 | cobalt uptake protein COT1 | IPR002524 : Cation efflux protein |
| MGG07565.6 | 0.07 | conserved hypothetical protein | IPR008427 : Extracellular membrane protein, 8-cysteine region, IPR014005 : Extracellular membrane protein, 8-cysteine region, fungi |
| MGG10031.6 | 0.06 | hypothetical protein | No defined Interpro term |
| MGG03598.6 | 0.06 | conserved hypothetical protein | IPR000209 : Peptidase S8 and S53, subtilisin, kexin, sedolisin, IPR002931 : Transglutaminase-like |
| MGG09388.6 | 0.06 | conserved hypothetical protein | IPR013027 : FAD-dependent pyridine nucleotide-disulphide oxidoreductase |
| MGG11271.6 | 0.06 | hypothetical protein | No defined Interpro term |
| MGG12894.6 | 0.06 | ATP-dependent RNA helicase | IPR000629 : RNA helicase, ATP-dependent, DEAD-box, conserved site, IPR001650 : DNA/RNA helicase, C-terminal, IPR011545 : DNA/RNA helicase, DEAD/DEAH box type, N-terminal |
| MGG02536.6 | 0.06 | conserved hypothetical protein | No defined Interpro term |
| MGG08758.6 | 0.06 | aminopeptidase Y | IPR003137 : Protease-associated PA, IPR007484 : Peptidase M28 |
| MGG07623.6 | 0.06 | hypothetical protein | IPR001002 : Chitin-binding, type 1 |
| MGG09779.6 | 0.06 | hypothetical protein | No defined Interpro term |
| MGG09021.6 | 0.06 | conserved hypothetical protein | No defined Interpro term |
| MGG05908.6 | 0.06 | cytochrome P450 52A11 | IPR001128 : Cytochrome P450, IPR002401 : Cytochrome P450, E-class, group I, IPR002974 : Cytochrome P450, E-class, CYP52 |
| MGG09863.6 | 0.05 | conserved hypothetical protein | No defined Interpro term |
| MGG04231.6 | 0.05 | hypothetical protein | IPR002110 : Ankyrin |
| MGG00888.6 | 0.05 | hypothetical protein | No defined Interpro term |
| MGG07615.6 | 0.04 | protoporphyrinogen oxidase | IPR013216 : Methyltransferase type 11 |
| MGG13137.6 | 0.04 | ABC1 family protein | IPR004147 : ABC-1,IPR004147 : ABC-1,IPR011009 : Protein kinase-like |
| MGG01127.6 | 0.04 | conserved hypothetical protein | IPR007087 : Zinc finger, C2H2-type, IPR013087 : Zinc finger, C2H2-type/integrase, DNA-binding, IPR015880 : Zinc finger, C2H2-like |
| MGG10197.6 | 0.04 | conserved hypothetical protein | IPR000054 : Ribosomal protein L31e, IPR007219 : Fungal specific transcription factor |
| MGG06446.6 | 0.04 | 5-aminolevulinate synthase, mitochondrial precursor | IPR001917 : Aminotransferase, class-II, pyridoxal-phosphate binding site, IPR004839 : Aminotransferase, class I and II, IPR010961 : Tetrapyrrole biosynthesis, 5-aminolevulinic acid synthase, |
| MGG07997.6 | 0.04 | covalently-linked cell wall protein | IPR000420 : Yeast PIR protein repeat |
| MGG08135.6 | 0.03 | conserved hypothetical protein | IPR000731 : Sterol-sensing 5TM box, IPR001680 : WD40 repeat, IPR003392 : Patched, IPR011046 : WD40 repeat-like |
| MGG12588.6 | 0.02 | hypothetical protein | IPR016160 : Aldehyde dehydrogenase, conserved site |
| MGG02482.6 | 0.02 | DNA replication licensing factor mcm10 | IPR004365 : Nucleic acid binding, OB-fold, tRNA/helicase-type, IPR015408 : Zinc finger, Mcm10/DnaG-type |
| MGG01046.6 | 0.02 | methionyl-tRNA synthetase | IPR001412 : Aminoacyl-tRNA synthetase, class I, conserved site, IPR002304 : Methionyl-tRNA synthetase, class Ia, IPR009080 : Aminoacyl-tRNA synthetase, class 1a, anticodon-binding |

^a^ Reduction ratios from the microarray analysis are calculated as the expression in the *ΔMohox2* mutant divided by the wild-type during conidiation.
