# Supplementary material for: Gene Expression Profiling during Conidiation in the Rice Blast Pathogen Magnaporthe oryzae
Source: PLoS One. 2012 Aug 21;7(8):e43202. doi: 10.1371/journal.pone.0043202 (PMC3424150; doi:10.1371/journal.pone.0043202)
Supplement: Table S6 — Genes induced during conidiation in the ΔMohox2 mutant. (DOCX) [file pone.0043202.s006.docx]

**Table S6.** Genes induced during conidiation in the *ΔMohox2* mutant.

| **Locus** | **Fold-induction during conidiation in the *ΔMohox2* mutant^a^** | **Annotation** | **InterPro domain search** |
| --- | --- | --- | --- |
| MGG08757.6 | 35.79 | DNA binding regulatory protein AmdX | IPR007087 : Zinc finger, C2H2-type, IPR013087 : Zinc finger, C2H2-type/integrase, DNA-binding |
| MGG01742.6 | 27.74 | elongation factor 2 | IPR000640 : Translation elongation factor EFG/EF2 |
| MGG02572.6 | 24.00 | prion formation protein 1 | IPR000953 : Chromo domain, IPR003439 : ABC transporter-like, IPR003593 : ATPase, AAA+ type, core, IPR015688 : Elongation Factor 3 |
| MGG02394.6 | 21.71 | malic acid transport protein | IPR004695 : C4-dicarboxylate transporter/malic acid transport protein |
| MGG09082.6 | 19.76 | conserved hypothetical protein | No defined Interpro term |
| MGG14603.6 | 19.39 | hypothetical protein | No defined Interpro term |
| MGG03360.6 | 16.51 | carboxylic acid transport protein | IPR011701 : Major facilitator superfamily MFS-1, IPR016196 : Major facilitator superfamily, general substrate transporter |
| MGG09198.6 | 15.34 | ent-kaurene oxidase | IPR001128 : Cytochrome P450 |
| MGG07000.6 | 15.32 | ribonucleoside-diphosphate reductase large chain | IPR000788 : Ribonucleotide reductase large subunit, C-terminal, IPR005144 : ATP-cone |
| MGG03272.6 | 14.67 | conserved hypothetical protein | No defined Interpro term |
| MGG06234.6 | 14.36 | hypothetical protein | No defined Interpro term |
| MGG03348.6 | 11.94 | phosphate-repressible phosphate permease | IPR001204 : Phosphate transporter,IPR001204 : Phosphate transporter |
| MGG06033.6 | 11.59 | hypothetical protein | No defined Interpro term |
| MGG01063.6 | 10.95 | conserved hypothetical protein | No defined Interpro term |
| MGG08130.6 | 10.89 | conserved hypothetical protein | IPR001138 : Fungal transcriptional regulatory protein |
| MGG00143.6 | 10.37 | conserved hypothetical protein | IPR014898 : Zinc finger, C2H2, LYAR-type |
| MGG07958.6 | 10.03 | naringenin,2-oxoglutarate 3-dioxygenase | IPR005123 : 2OG-Fe(II) oxygenase |
| MGG08482.6 | 9.94 | hypothetical protein | No defined Interpro term |
| MGG02842.6 | 9.70 | hsp70-like protein | IPR001023 : Heat shock protein Hsp70 , IPR013126 : Heat shock protein 70 |
| MGG06330.6 | 9.62 | conserved hypothetical protein | No defined Interpro term |
| MGG10359.6 | 9.58 | conserved hypothetical protein | No defined Interpro term |
| MGG15264.6 | 9.31 | hypothetical protein | No defined Interpro term |
| MGG09639.6 | 9.09 | cell wall alpha-1,3-glucan synthase ags1 | IPR001296 : Glycosyl transferase, group 1, IPR013534 : Starch synthase catalytic region, IPR013781 : Glycoside hydrolase, subgroup, catalytic core |
| MGG02223.6 | 9.04 | conserved hypothetical protein | No defined Interpro term |
| MGG04327.6 | 9.03 | conserved hypothetical protein | IPR002893 : Zinc finger, MYND-type |
| MGG03657.6 | 8.89 | conserved hypothetical protein | IPR002190 : MAGE protein |
| MGG05788.6 | 8.72 | Poly(3-hydroxybutyrate) depolymerase | No defined Interpro term |
| MGG10799.6 | 8.54 | acid phosphatase PHO1 | IPR000560 : Histidine acid phosphatase |
| MGG00574.6 | 8.46 | galactan 1,3-beta-galactosidase | IPR006710 : Glycoside hydrolase, family 43 |
| MGG01896.6 | 8.42 | DJ-1/PfpI family protein | IPR002818 : ThiJ/PfpI,IPR002818 : ThiJ/PfpI |
| MGG02649.6 | 8.22 | conserved hypothetical protein | IPR000209 : Peptidase S8 and S53, subtilisin, kexin, sedolisin, IPR003137 : Protease-associated PA |
| MGG02059.6 | 8.17 | conserved hypothetical protein | No defined Interpro term |
| MGG04865.6 | 8.05 | conserved hypothetical protein | No defined Interpro term |
| MGG10671.6 | 7.87 | pentalenene synthase | IPR005630 : Terpene synthase, metal-binding domain |
| MGG07900.6 | 7.85 | hypothetical protein | No defined Interpro term |
| MGG08577.6 | 7.81 | conserved hypothetical protein | No defined Interpro term |
| MGG15397.6 | 7.80 | hypothetical protein | No defined Interpro term |
| MGG04969.6 | 7.63 | cytochrome c oxidase assembly protein COX15 | IPR003780 : Cytochrome oxidase assembly |
| MGG07429.6 | 7.62 | conserved hypothetical protein | No defined Interpro term |
| MGG01734.6 | 7.53 | C6 zinc finger domain-containing protein | IPR001138 : Fungal transcriptional regulatory protein, N-terminal |
| MGG07100.6 | 7.53 | hypothetical protein | No defined Interpro term |
| MGG08395.6 | 7.43 | hypothetical protein | No defined Interpro term |
| MGG07577.6 | 7.43 | conserved hypothetical protein | No defined Interpro term |
| MGG07528.6 | 7.38 | imidazoleglycerol-phosphate dehydratase | IPR000807 : Imidazole glycerol-phosphate dehydratase |
| MGG09146.6 | 7.35 | conserved hypothetical protein | IPR007568 : RTA1 like protein |
| MGG02918.6 | 7.19 | hypothetical protein | IPR001064 : Beta and gamma crystallin |
| MGG02812.6 | 7.13 | 1-aminocyclopropane-1-carboxylate deaminase | IPR001926 : Pyridoxal phosphate-dependent enzyme, beta subunit, IPR005965 : 1-aminocyclopropane-1-carboxylate deaminase |
| MGG05790.6 | 7.05 | laccase-1 | IPR001117 : Multicopper oxidase, type 1, IPR008972 : Cupredoxin |
| MGG12760.6 | 7.01 | anthranilate synthase component 1 | IPR005256 : Anthranilate synthase component I |
| MGG01534.6 | 7.01 | conserved hypothetical protein | No defined Interpro term |
| MGG07621.6 | 6.98 | endoribonuclease L-PSP | IPR006175 : Endoribonuclease L-PSP |
| MGG06008.6 | 6.95 | hypothetical protein | No defined Interpro term |
| MGG08962.6 | 6.88 | conserved hypothetical protein | No defined Interpro term |
| MGG09817.6 | 6.81 | minor extracellular protease vpr | IPR000209 : Peptidase S8 and S53, subtilisin, kexin, sedolisin, IPR003137 : Protease-associated PA |
| MGG03374.6 | 6.70 | beta-1,6-galactanase | IPR001547 : Glycoside hydrolase, family 5 |
| MGG00692.6 | 6.68 | cell pattern formation-associated protein stuA | IPR003163 : APSES-type HTH DNA-binding domain, IPR016144 : |
| MGG05225.6 | 6.62 | conserved hypothetical protein | No defined Interpro term |
| MGG09920.6 | 6.53 | cytochrome P450 52A5 | IPR001128 : Cytochrome P450 |
| MGG06399.6 | 6.46 | serine/threonine-protein kinase ppk15 | IPR000719 : Protein kinase, core, IPR002290 : Serine/threonine protein kinase |
| MGG04631.6 | 6.41 | terpene synthase metal binding domain-containing protein | IPR008949 : Terpenoid synthase |
| MGG05844.6 | 6.40 | mannan endo-1,4-beta-mannosidase 1 precursor | IPR001547 : Glycoside hydrolase, family 5 |
| MGG09165.6 | 6.32 | hypothetical protein | No defined Interpro term |
| MGG11719.6 | 6.29 | hypothetical protein | No defined Interpro term |
| MGG10286.6 | 6.22 | conserved hypothetical protein | No defined Interpro term |
| MGG01862.6 | 6.13 | zinc/iron transporter protein | IPR003689 : Zinc/iron permease |
| MGG12016.6 | 6.10 | conserved hypothetical protein | IPR000675 : Cutinase |
| MGG04550.6 | 6.08 | calcium-transporting ATPase sarcoplasmic/endoplasmic reticulum type | IPR001757 : ATPase, P-type, K/Mg/Cd/Cu/Zn/Na/Ca/Na/H-transporter, IPR004014 : ATPase, P-type cation-transporter, N-terminal |
| MGG00496.6 | 6.02 | conserved hypothetical protein | IPR013248 : Shr3 amino acid permease chaperone |
| MGG07005.6 | 5.96 | hypothetical protein | IPR008427 : Extracellular membrane protein, 8-cysteine region, CFEM |
| MGG10574.6 | 5.95 | GNAT family N-acetyltransferase | IPR000182 : GCN5-related N-acetyltransferase |
| MGG14148.6 | 5.90 | conserved hypothetical protein | IPR017896 : 4Fe-4S ferredoxin, iron-sulpur binding domain |
| MGG08047.6 | 5.90 | glycerophosphoryl diester phosphodiesterase family protein | IPR004129 : Glycerophosphoryl diester phosphodiesterase |
| MGG04339.6 | 5.89 | conserved hypothetical protein | No defined Interpro term |
| MGG01458.6 | 5.80 | hypothetical protein | No defined Interpro term |
| MGG04406.6 | 5.80 | 26S proteasome non-ATPase regulatory subunit 10 | IPR002078 : RNA polymerase sigma factor 54, interaction, IPR002110 : Ankyrin |
| MGG10826.6 | 5.74 | conserved hypothetical protein | IPR000719 : Protein kinase, core, IPR008271 : Serine/threonine protein kinase, active site |
| MGG02961.6 | 5.70 | anucleate primary sterigmata protein A | IPR001849 : Pleckstrin homology |
| MGG10017.6 | 5.68 | NmrA family protein | IPR008030 : NmrA-like, IPR016040 : NAD(P)-binding |
| MGG12697.6 | 5.68 | pheromone-regulated membrane protein 10 | IPR010619 : Protein of unknown function DUF1212 |
| MGG06799.6 | 5.67 | conserved hypothetical protein | No defined Interpro term |
| MGG01666.6 | 5.63 | CCR4-NOT transcription complex subunit 7 | IPR006941 : Ribonuclease CAF1, IPR012337 : Polynucleotidyl transferase, Ribonuclease H fold |
| MGG02383.6 | 5.63 | hypothetical protein | No defined Interpro term |
| MGG10456.6 | 5.59 | hypothetical protein | No defined Interpro term |
| MGG03222.6 | 5.54 | hypothetical protein | IPR013665 : Sfi1 spindle body |
| MGG03383.6 | 5.46 | hypothetical protein | No defined Interpro term |
| MGG04878.6 | 5.37 | Lid2 complex component lid2 | IPR001304 : C-type lectin, IPR001606 : AT-rich interaction region, IPR001965 : Zinc finger, PHD-type, IPR003347 : Transcription factor jumonji/aspartyl beta-hydroxylase |
| MGG07964.6 | 5.34 | conserved hypothetical protein | IPR013217 : Methyltransferase type 12 |
| MGG07202.6 | 5.33 | citrate synthase | IPR002020 : Citrate synthase-like, IPR010109 : Citrate synthase, eukaryotic |
| MGG05016.6 | 5.31 | bZIP transcription factor | IPR004827 : Basic-leucine zipper (bZIP) transcription factor |
| MGG00324.6 | 5.29 | dienelactone hydrolase family protein | IPR002925 : Dienelactone hydrolase |
| MGG11610.6 | 5.28 | hypothetical protein | No defined Interpro term |
| MGG03079.6 | 5.24 | hypothetical protein | No defined Interpro term |
| MGG06624.6 | 5.14 | hypothetical protein | No defined Interpro term |
| MGG10487.6 | 5.07 | hypothetical protein | No defined Interpro term |
| MGG03177.6 | 5.05 | hypothetical protein | No defined Interpro term |
| MGG04661.6 | 5.02 | conserved hypothetical protein | IPR013243 : SCA7 |
| MGG03595.6 | 5.02 | G2/mitotic-specific cyclin-B1 | IPR004367 : Cyclin, C-terminal |
| MGG02284.6 | 4.98 | conserved hypothetical protein | No defined Interpro term |
| MGG08916.6 | 4.97 | hypothetical protein | No defined Interpro term |
| MGG00151.6 | 4.97 | WD repeat-containing protein | IPR001680 : WD40 repeat |
| MGG10702.6 | 4.96 | conserved hypothetical protein | IPR004838 : Aminotransferases, class-I, pyridoxal-phosphate-binding site |
| MGG04370.6 | 4.94 | conserved hypothetical protein | No defined Interpro term |
| MGG02268.6 | 4.93 | conserved hypothetical protein | No defined Interpro term |
| MGG08759.6 | 4.84 | hypothetical protein | No defined Interpro term |
| MGG04738.6 | 4.84 | bacilysin biosynthesis oxidoreductase bacC | IPR002198 : Short-chain dehydrogenase/reductase SDR, IPR002347 : Glucose/ribitol dehydrogenase, IPR016040 : NAD(P)-binding |
| MGG13177.6 | 4.83 | cytosolic Cu/Zn superoxide dismutase | IPR001424 : Superoxide dismutase, copper/zinc binding |
| MGG03701.6 | 4.82 | conserved hypothetical protein | IPR007114 : , IPR011701 : Major facilitator superfamily MFS-1, IPR016196 : Major facilitator superfamily, general substrate transporter |
| MGG05442.6 | 4.76 | endo alpha-1,4 polygalactosaminidase precusor | No defined Interpro term |
| MGG10595.6 | 4.70 | conserved hypothetical protein | IPR007087 : Zinc finger, C2H2-type |
| MGG01994.6 | 4.68 | conserved hypothetical protein | IPR016040 : NAD(P)-binding |
| MGG02692.6 | 4.67 | hypothetical protein | No defined Interpro term |
| MGG10704.6 | 4.65 | hypothetical protein | IPR000182 : GCN5-related N-acetyltransferase, IPR016181 : Acyl-CoA N-acyltransferase |
| MGG04338.6 | 4.60 | potassium transport protein 1 | IPR003445 : Cation transporter |
| MGG04988.6 | 4.60 | chromosome segregation protein sudA | IPR003395 : RecF/RecN/SMC protein, N-terminal, IPR010935 : SMCs flexible hinge |
| MGG08501.6 | 4.58 | conserved hypothetical protein | No defined Interpro term |
| MGG02062.6 | 4.52 | hypothetical protein | No defined Interpro term |
| MGG12108.6 | 4.51 | leucine Rich Repeat domain-containing protein |  |
| MGG06533.6 | 4.50 | hypothetical protein | No defined Interpro term |
| MGG07450.6 | 4.50 | fungal specific transcription factor domain-containing protein | IPR007219 : Fungal specific transcription factor |
| MGG03880.6 | 4.50 | alcohol dehydrogenase 1 | IPR002085 : Alcohol dehydrogenase superfamily, zinc-containing, IPR016040 : NAD(P)-binding |
| MGG14883.6 | 4.46 | methyltransferase small domain-containing protein | IPR013216 : Methyltransferase type 11 |
| MGG03228.6 | 4.43 | conserved hypothetical protein | IPR007244 : Mak10 subunit, NatC N(alpha)-terminal acetyltransferase |
| MGG07269.6 | 4.42 | conserved hypothetical protein | IPR013087 : Zinc finger, C2H2-type/integrase, DNA-binding |
| MGG04361.6 | 4.41 | conserved hypothetical protein | No defined Interpro term |
| MGG05654.6 | 4.41 | conserved hypothetical protein | No defined Interpro term |
| MGG04831.6 | 4.41 | conserved hypothetical protein | IPR001680 : WD40 repeat, IPR005829 : Sugar transporter, conserved site |
| MGG08898.6 | 4.40 | conserved hypothetical protein | IPR000504 : RNA recognition motif, RNP-1, IPR012677 : Nucleotide-binding, alpha-beta plait |
| MGG04777.6 | 4.39 | antibiotic biosynthesis monooxygenase | IPR011008 : Dimeric alpha-beta barrel |
| MGG13535.6 | 4.38 | nucleoporin POM152 |  |
| MGG09736.6 | 4.38 | hypothetical protein | No defined Interpro term |
| MGG06504.6 | 4.32 | 26S proteasome regulatory subunit RPN7 | IPR000717 : Proteasome component region PCI |
| MGG04456.6 | 4.32 | zinc finger protein 664 | IPR013087 : Zinc finger, C2H2-type/integrase, DNA-binding |
| MGG08988.6 | 4.31 | conserved hypothetical protein | IPR010730 : Heterokaryon incompatibility |
| MGG07274.6 | 4.30 | conserved hypothetical protein | No defined Interpro term |
| MGG03675.6 | 4.28 | conserved hypothetical protein | No defined Interpro term |
| MGG13261.6 | 4.27 | conserved hypothetical protein | IPR008972 : Cupredoxin |
| MGG04895.6 | 4.26 | isocitrate lyase | IPR000918 : Isocitrate lyase and phosphorylmutase, IPR006254 : Isocitrate lyase |
| MGG01134.6 | 4.25 | UTR2 protein | IPR000757 : Glycoside hydrolase, family 16, IPR008197 : Whey acidic protein, 4-disulphide core |
| MGG01613.6 | 4.25 | molybdenum cofactor sulfurase | IPR000192 : Aminotransferase, class V/Cysteine desulfurase, IPR005302 : Molybdenum cofactor sulfurase, C-terminal |
| MGG13464.6 | 4.24 | laccase | IPR001117 : Multicopper oxidase, type 1, IPR008972 : Cupredoxin, |
| MGG05988.6 | 4.24 | lipid phosphate phosphatase 2 | IPR000326 : Phosphatidic acid phosphatase type 2/haloperoxidase |
| MGG15364.6 | 4.22 | hypothetical protein | No defined Interpro term |
| MGG05224.6 | 4.20 | conserved hypothetical protein | IPR001965 : Zinc finger, PHD-type |
| MGG08944.6 | 4.20 | hypothetical protein | No defined Interpro term |
| MGG06782.6 | 4.19 | N amino acid transport system protein | IPR013057 : Amino acid transporter, transmembrane |
| MGG15383.6 | 4.19 | glutamyl-tRNA(Gln) amidotransferase | IPR000120 : Amidase signature enzyme |
| MGG03764.6 | 4.18 | salicylate hydroxylase | IPR006076 : FAD dependent oxidoreductase |
| MGG03338.6 | 4.14 | cellulose-binding protein | IPR001087 : Lipase, GDSL, IPR013830 : Esterase, SGNH hydrolase-type, IPR013831 : Esterase, SGNH hydrolase-type, subgroup |
| MGG06180.6 | 4.12 | endocytosis and cytoskeletal organization protein | IPR000261 : EPS15 homology (EH), IPR002048 : Calcium-binding EF-hand, IPR011992 : EF-Hand type |
| MGG00283.6 | 4.11 | hypothetical protein | No defined Interpro term |
| MGG09648.6 | 4.11 | conserved hypothetical protein | No defined Interpro term |
| MGG13615.6 | 4.10 | aquaporin-9 |  |
| MGG12130.6 | 4.09 | serine/threonine protein phosphatase 2A | IPR002554 : Protein phosphatase 2A, regulatory B subunit, B56 |
| MGG04358.6 | 4.08 | hypothetical protein | IPR002068 : Heat shock protein Hsp20 |
| MGG01090.6 | 4.08 | hypothetical protein | IPR001092 : Basic helix-loop-helix dimerisation region bHLH, IPR011598 : Helix-loop-helix DNA-binding |
| MGG09677.6 | 4.08 | conserved hypothetical protein | IPR011118 : Tannase and feruloyl esterase |
| MGG11391.6 | 4.07 | conserved hypothetical protein | IPR006773 : Adhesion regulating molecule |
| MGG07631.6 | 4.06 | fungal cellulose binding domain-containing protein | No defined Interpro term |
| MGG07015.6 | 4.04 | DNA repair protein Rad7 | No defined Interpro term |
| MGG04369.6 | 4.03 | spastin | IPR003593 : ATPase, AAA+ type, core |
| MGG09181.6 | 4.03 | hydroxymethylglutaryl-CoA lyase | IPR000891 : Pyruvate carboxyltransferase |
| MGG04172.6 | 4.02 | polysaccharide deacetylase | IPR002509 : Polysaccharide deacetylase, IPR011330 : Glycoside hydrolase/deacetylase, beta/alpha-barrel |
| MGG03625.6 | 3.99 | conserved hypothetical protein | No defined Interpro term |
| MGG04017.6 | 3.92 | L-asparaginase | IPR002110 : Ankyrin, IPR006034 : Asparaginase/glutaminase |
| MGG07993.6 | 3.92 | hypothetical protein | No defined Interpro term |
| MGG10593.6 | 3.92 | hypothetical protein | No defined Interpro term |
| MGG03276.6 | 3.91 | major allergen Asp f 2 | No defined Interpro term |
| MGG01085.6 | 3.91 | ThiJ/PfpI family protein | IPR002818 : ThiJ/PfpI |
| MGG10004.6 | 3.88 | hypothetical protein | No defined Interpro term |
| MGG01888.6 | 3.87 | WD repeat-containing protein | IPR001680 : WD40 repeat, IPR011046 : WD40 repeat-like |
| MGG02119.6 | 3.85 | high-affinity nicotinic acid transporter | IPR007114 : , IPR011701 : Major facilitator superfamily MFS-1, IPR016196 : Major facilitator superfamily, general substrate transporter |
| MGG11047.6 | 3.81 | DNA repair and recombination protein RAD5C | IPR000330 : SNF2-related, IPR001650 : DNA/RNA helicase, C-terminal, IPR001841 : Zinc finger, RING-type |
| MGG00110.6 | 3.80 | conserved hypothetical protein | IPR013830 : Esterase, SGNH hydrolase-type, IPR013831 : Esterase, SGNH hydrolase-type, subgroup |
| MGG07276.6 | 3.80 | conserved hypothetical protein | No defined Interpro term |
| MGG15092.6 | 3.80 | hypothetical protein | No defined Interpro term |
| MGG10279.6 | 3.79 | conserved hypothetical protein | IPR001279 : Beta-lactamase-like |
| MGG00461.6 | 3.79 | conserved hypothetical protein | IPR002016 : Haem peroxidase, plant/fungal/bacterial, IPR010255 : Haem peroxidase |
| MGG10287.6 | 3.78 | metabolite transporter | IPR005828 : General substrate transporter, IPR005829 : Sugar transporter, conserved site |
| MGG10235.6 | 3.78 | hypothetical protein | No defined Interpro term |
| MGG07652.6 | 3.73 | class II aldolase/adducin domain-containing protein | IPR000169 : Peptidase, cysteine peptidase active site, IPR001303 : Class II aldolase/adducin, N-terminal |
| MGG06261.6 | 3.73 | eukaryotic translation initiation factor 3 135 kDa subunit | IPR011990 : Tetratricopeptide-like helical, IPR013026 : Tetratricopeptide region |
| MGG04248.6 | 3.73 | conserved hypothetical protein | IPR004813 : Oligopeptide transporter OPT superfamily |
| MGG04929.6 | 3.72 | conserved hypothetical protein | IPR011028 : Cyclin-like, IPR013922 : Cyclin-related 2 |
| MGG03170.6 | 3.69 | conserved hypothetical protein | No defined Interpro term |
| MGG09972.6 | 3.67 | conserved hypothetical protein | IPR005829 : Sugar transporter, conserved site, IPR007114 : , IPR011701 : Major facilitator superfamily MFS-1 |
| MGG03081.6 | 3.66 | conserved hypothetical protein | IPR002654 : Glycosyl transferase, family 25 |
| MGG01754.6 | 3.65 | conserved hypothetical protein | IPR013105 : Tetratricopeptide TPR2, IPR013143 : PCI/PINT associated module |
| MGG09807.6 | 3.65 | hypothetical protein | No defined Interpro term |
| MGG03196.6 | 3.63 | conserved hypothetical protein | IPR001440 : Tetratricopeptide TPR-1 |
| MGG02582.6 | 3.62 | conserved hypothetical protein | No defined Interpro term |
| MGG14010.6 | 3.62 | hypothetical protein | IPR002110 : Ankyrin |
| MGG01296.6 | 3.57 | spermine/spermidine synthase family protein | IPR001045 : Spermine synthase |
| MGG09102.6 | 3.57 | laccase-1 | IPR001117 : Multicopper oxidase, type 1, IPR002355 : Multicopper oxidase, copper-binding site, IPR008972 : Cupredoxin |
| MGG00190.6 | 3.55 | conserved hypothetical protein | IPR001092 : Basic helix-loop-helix dimerisation region bHLH, IPR011598 : Helix-loop-helix DNA-binding |
| MGG04157.6 | 3.55 | hypothetical protein | IPR010730 : Heterokaryon incompatibility |
| MGG05789.6 | 3.54 | hypothetical protein | No defined Interpro term |
| MGG13793.6 | 3.52 | nitrate transporter | IPR004737 : Nitrate transporter, IPR011701 : Major facilitator superfamily MFS-1 |
| MGG05075.6 | 3.52 | hypothetical protein | No defined Interpro term |
| MGG10928.6 | 3.50 | conserved hypothetical protein | No defined Interpro term |
| MGG06538.6 | 3.48 | Bys1 family protein | No defined Interpro term |
| MGG02940.6 | 3.46 | UBX domain-containing protein 8 | IPR001012 : UBX |
| MGG13137.6 | 3.44 | ABC1 family protein |  |
| MGG04728.6 | 3.43 | hypothetical protein | No defined Interpro term |
| MGG02755.6 | 3.43 | nitrogen regulatory protein NUT1 | IPR000679 : Zinc finger, GATA-type, IPR013088 : Zinc finger, NHR/GATA-type, |
| MGG12981.6 | 3.43 | cupin domain-containing protein | IPR011051 : Cupin, RmlC-type, IPR013096 : Cupin 2, conserved barrel |
| MGG05769.6 | 3.42 | hypothetical protein | No defined Interpro term |
| MGG11021.6 | 3.41 | conserved hypothetical protein | IPR003593 : ATPase, AAA+ type, core |
| MGG08965.6 | 3.41 | conserved hypothetical protein | No defined Interpro term |
| MGG03026.6 | 3.37 | G2-specific protein kinase nim-1 | IPR000719 : Protein kinase, core, IPR001245 : Tyrosine protein kinase, IPR002290 : Serine/threonine protein kinase |
| MGG09928.6 | 3.37 | conserved hypothetical protein | No defined Interpro term |
| MGG06361.6 | 3.36 | dynamin-A | IPR000375 : Dynamin central region, IPR001401 : Dynamin, GTPase region, IPR003130 : Dynamin GTPase effector |
| MGG07355.6 | 3.36 | hypothetical protein | No defined Interpro term |
| MGG07819.6 | 3.35 | conserved hypothetical protein | IPR001241 : DNA topoisomerase, type IIA, subunit B or N-terminal |
| MGG08724.6 | 3.34 | glycosyltransferase | IPR000111 : Glycoside hydrolase, clan GH-D, IPR002213 : UDP-glucuronosyl/UDP-glucosyltransferase, IPR006158 : Cobalamin (vitamin B12)-binding |
| MGG00702.6 | 3.34 | hypothetical protein | No defined Interpro term |
| MGG07143.6 | 3.33 | conserved hypothetical protein | IPR013964 : DASH complex, subunit Ask1 |
| MGG06549.6 | 3.31 | conserved hypothetical protein | No defined Interpro term |
| MGG10684.6 | 3.30 | conserved hypothetical protein | No defined Interpro term |
| MGG00750.6 | 3.29 | cytochrome b-245 heavychain subunit beta | IPR013112 : FAD-binding 8, IPR013121 : Ferric reductase, NAD binding |
| MGG12214.6 | 3.27 | fatty acid synthase S-acetyltransferase | IPR000794 : Beta-ketoacyl synthase, IPR001227 : Acyl transferase region, IPR006163 : Phosphopantetheine-binding |
| MGG00505.6 | 3.27 | septation protein SUN4 | IPR005556 : SUN |
| MGG14953.6 | 3.26 | hypothetical protein | No defined Interpro term |
| MGG02489.6 | 3.25 | branched-chain-amino-acid aminotransferase | IPR001544 : Aminotransferase, class IV, IPR005786 : Branched-chain amino acid aminotransferase II, |
| MGG15337.6 | 3.25 | zygote-specific protein | No defined Interpro term |
| MGG09017.6 | 3.24 | rieske domain-containing protein | IPR002048 : Calcium-binding EF-hand, IPR005806 : Rieske [2Fe-2S] region, IPR017941 : Rieske [2Fe-2S] iron-sulphur domain |
| MGG02846.6 | 3.24 | conserved hypothetical protein | No defined Interpro term |
| MGG03324.6 | 3.23 | conserved hypothetical protein | IPR005123 : 2OG-Fe(II) oxygenase |
| MGG08389.6 | 3.22 | conserved hypothetical protein | IPR002198 : Short-chain dehydrogenase/reductase SDR, IPR002347 : Glucose/ribitol dehydrogenase, IPR016040 : NAD(P)-binding |
| MGG00581.6 | 3.22 | hypothetical protein | No defined Interpro term |
| MGG09786.6 | 3.21 | conserved hypothetical protein | No defined Interpro term |
| MGG13517.6 | 3.21 | hypothetical protein | No defined Interpro term |
| MGG00718.6 | 3.20 | hypothetical protein | IPR008914 : Phosphatidylethanolamine-binding protein PEBP |
| MGG13009.6 | 3.20 | hypothetical protein | No defined Interpro term |
| MGG08845.6 | 3.20 | conserved hypothetical protein | No defined Interpro term |
| MGG06672.6 | 3.19 | hypothetical protein | No defined Interpro term |
| MGG09785.6 | 3.18 | conserved hypothetical protein | IPR002198 : Short-chain dehydrogenase/reductase SDR, IPR002347 : Glucose/ribitol dehydrogenase, IPR016040 : NAD(P)-binding |
| MGG11604.6 | 3.17 | conserved hypothetical protein | IPR000215 : Protease inhibitor I4, serpin, IPR001810 : Cyclin-like F-box |
| MGG04434.6 | 3.17 | DNA replication complex GINS protein PSF1 | IPR005339 : GINS complex, Psf1 component |
| MGG10558.6 | 3.15 | conserved hypothetical protein | IPR010730 : Heterokaryon incompatibility |
| MGG06661.6 | 3.15 | high-affinity nicotinic acid transporter | IPR007114 : , IPR011701 : Major facilitator superfamily MFS-1, IPR016196 : Major facilitator superfamily, general substrate transporter |
| MGG14640.6 | 3.15 | mating-type switching protein swi10 |  |
| MGG09223.6 | 3.14 | alpha-N-arabinofuranosidase 2 | IPR006710 : Glycoside hydrolase, family 43 |
| MGG05367.6 | 3.13 | glutathione transferase omega-1 | IPR004045 : Glutathione S-transferase, N-terminal |
| MGG15084.6 | 3.13 | hypothetical protein | No defined Interpro term |
| MGG15365.6 | 3.13 | hypothetical protein | No defined Interpro term |
| MGG15408.6 | 3.13 | conserved hypothetical protein | No defined Interpro term |
| MGG02374.6 | 3.11 | hypothetical protein | IPR001810 : Cyclin-like F-box |
| MGG10005.6 | 3.10 | glycerol kinase | IPR000577 : Carbohydrate kinase, FGGY, IPR005999 : Glycerol kinase |
| MGG04943.6 | 3.10 | mitogen-activated protein kinase spm1 | IPR000719 : Protein kinase, core, IPR002290 : Serine/threonine protein kinase, IPR003527 : MAP kinase, conserved site, |
| MGG00672.6 | 3.10 | C6 zinc finger domain-containing protein | IPR001138 : Fungal transcriptional regulatory protein, N-terminal |
| MGG07272.6 | 3.09 | conserved hypothetical protein | IPR007235 : Glycosyl transferase, family 28, C-terminal |
| MGG15085.6 | 3.09 | conserved hypothetical protein | IPR001138 : Fungal transcriptional regulatory protein, N-terminal, IPR007219 : Fungal specific transcription factor |
| MGG03241.6 | 3.09 | arrestin domain-containing protein | IPR011022 : Arrestin-like, C-terminal |
| MGG10760.6 | 3.09 | FAD-binding domain-containing protein | IPR013112 : FAD-binding 8, IPR017927 : Ferredoxin reductase-type FAD-binding domain |
| MGG02758.6 | 3.09 | conserved hypothetical protein | No defined Interpro term |
| MGG10474.6 | 3.08 | hypothetical protein | No defined Interpro term |
| MGG12317.6 | 3.06 | hypothetical protein | No defined Interpro term |
| MGG15353.6 | 3.05 | conserved hypothetical protein | No defined Interpro term |
| MGG08591.6 | 3.04 | conserved hypothetical protein | IPR000577 : Carbohydrate kinase, FGGY, IPR006003 : Carbohydrate kinase, FGGY-related |
| MGG07140.6 | 3.04 | conserved hypothetical protein | IPR007087 : Zinc finger, C2H2-type, IPR013087 : Zinc finger, C2H2-type/integrase, DNA-binding, IPR015880 : Zinc finger, C2H2-like |
| MGG00220.6 | 3.03 | NADP-dependent alcohol dehydrogenase 6 | IPR002085 : Alcohol dehydrogenase superfamily, zinc-containing, IPR002328 : Alcohol dehydrogenase, zinc-containing, conserved site |
| MGG00171.6 | 3.02 | mannan polymerase complexes MNN9 subunit | IPR005109 : Anp1 |
| MGG04543.6 | 3.01 | conserved hypothetical protein | No defined Interpro term |
| MGG09032.6 | 3.00 | aspergillopepsin-2 | IPR000250 : Peptidase G1, eqolisin, IPR008985 : Concanavalin A-like lectin/glucanase |
| MGG13161.6 | 2.99 | conserved hypothetical protein | No defined Interpro term |
| MGG13026.6 | 2.98 | hypothetical protein | No defined Interpro term |
| MGG04368.6 | 2.96 | stage V sporulation protein K | IPR003593 : ATPase, AAA+ type, core |
| MGG02269.6 | 2.96 | conserved hypothetical protein | IPR002654 : Glycosyl transferase, family 25 |
| MGG07697.6 | 2.96 | superoxide dismutase | IPR001189 : Manganese and iron superoxide dismutase |
| MGG10300.6 | 2.95 | conserved hypothetical protein | No defined Interpro term |
| MG00080.6 | 2.95 | hypothetical protein | No defined Interpro term |
| MGG07971.6 | 2.94 | calcium-transporting ATPase 1 | IPR001757 : ATPase, P-type, K/Mg/Cd/Cu/Zn/Na/Ca/Na/H-transporter, IPR004014 : ATPase, P-type cation-transporter, N-terminal |
| MGG06144.6 | 2.93 | hypothetical protein |  |
| MGG10973.6 | 2.93 | conserved hypothetical protein | No defined Interpro term |
| MGG00521.6 | 2.92 | conserved hypothetical protein | No defined Interpro term |
| MGG06971.6 | 2.92 | flocculation suppression protein | IPR000232 : Heat shock factor (HSF)-type, DNA-binding, IPR011991 : Winged helix repressor DNA-binding |
| MGG01467.6 | 2.91 | transmembrane protein 34 | IPR005178 : Protein of unknown function DUF300 |
| MGG11430.6 | 2.90 | WD repeat-containing protein | IPR001680 : WD40 repeat |
| MGG04252.6 | 2.90 | conserved hypothetical protein | IPR009007 : Peptidase aspartic, catalytic |
| MGG07847.6 | 2.90 | hypothetical protein | IPR007110 : Immunoglobulin-like |
| MGG09795.6 | 2.89 | conserved hypothetical protein | IPR006461 : Protein of unknown function Cys-rich |
| MGG15138.6 | 2.88 | conserved hypothetical protein | No defined Interpro term |
| MGG15113.6 | 2.88 | pyridoxal reductase | IPR001395 : Aldo/keto reductase |
| MGG03607.6 | 2.88 | alanyl-tRNA synthetase | IPR002318 : Alanyl-tRNA synthetase, class IIc, IPR003156 : Phosphoesterase, DHHA1, IPR012947 : Threonyl/alanyl tRNA synthetase, SAD |
| MGG06853.6 | 2.87 | conserved hypothetical protein | No defined Interpro term |
| MGG07464.6 | 2.87 | conserved hypothetical protein | No defined Interpro term |
| MGG08072.6 | 2.86 | cholesterol oxidase | IPR000172 : Glucose-methanol-choline oxidoreductase, N-terminal |
| MGG15026.6 | 2.85 | choline dehydrogenase | IPR012132 : Glucose-methanol-choline oxidoreductase |
| MGG08900.6 | 2.84 | WD40 domain-containing protein | IPR011046 : WD40 repeat-like, IPR015943 : WD40/YVTN repeat-like |
| MGG15056.6 | 2.84 | hypothetical protein | No defined Interpro term |
| MGG02592.6 | 2.84 | hypothetical protein | No defined Interpro term |
| MGG02906.6 | 2.84 | conserved hypothetical protein | No defined Interpro term |
| MGG03715.6 | 2.83 | hypothetical protein | No defined Interpro term |
| MGG02372.6 | 2.83 | hypothetical protein | No defined Interpro term |
| MGG07538.6 | 2.82 | hypothetical protein | No defined Interpro term |
| MGG05197.6 | 2.82 | serine palmitoyltransferase 2 | IPR004839 : Aminotransferase, class I and II, IPR015421 : Pyridoxal phosphate-dependent transferase, major region, subdomain 1 |
| MGG14720.6 | 2.81 | hypothetical protein | No defined Interpro term |
| MGG04869.6 | 2.81 | esterase/lipase/thioesterase | IPR013094 : Alpha/beta hydrolase fold-3 |
| MGG00963.6 | 2.80 | hypothetical protein | No defined Interpro term |
| MGG01151.6 | 2.78 | integral membrane protein | No defined Interpro term |
| MGG00865.6 | 2.78 | 1,3-beta-glucan synthase component GLS1 | IPR003440 : Glycosyl transferase, family 48 |
| MGG10663.6 | 2.77 | cAMP-regulated D2 protein | IPR002018 : Carboxylesterase, type B |
| MGG11887.6 | 2.77 | conserved hypothetical protein | No defined Interpro term |
| MGG01017.6 | 2.77 | conserved hypothetical protein | IPR007087 : Zinc finger, C2H2-type |
| MGG13913.6 | 2.76 | tetraspanin Tsp3 | No defined Interpro term |
| MGG07619.6 | 2.76 | taurine catabolism dioxygenase TauD | IPR003819 : Taurine catabolism dioxygenase TauD/TfdA |
| MGG02543.6 | 2.76 | conserved hypothetical protein | IPR001087 : Lipase, GDSL, IPR013830 : Esterase, SGNH hydrolase-type, IPR013831 : Esterase, SGNH hydrolase-type, subgroup |
| MGG04640.6 | 2.76 | sulfate permease 2 | IPR001902 : Sulphate anion transporter, IPR002645 : Sulphate transporter/antisigma-factor antagonist STAS |
| MGG01444.6 | 2.75 | hypothetical protein | No defined Interpro term |
| MGG09860.6 | 2.73 | hypothetical protein | No defined Interpro term |
| MGG04030.6 | 2.72 | DUF250 domain membrane protein | IPR004853 : Protein of unknown function DUF250 |
| MGG08279.6 | 2.71 | inner membrane protein yicO | IPR006043 : Xanthine/uracil/vitamin C permease |
| MGG04449.6 | 2.71 | branched-chain alpha-keto acid lipoamide acyltransferase | IPR003016 : 2-oxo acid dehydrogenase, lipoyl-binding site, IPR004167 : E3 binding, IPR011053 : Single hybrid motif, IPR015761 : Lipoamide Acyltransferase |
| MGG06328.6 | 2.71 | C2H2 type zinc finger domain-containing protein | IPR007087 : Zinc finger, C2H2-type, IPR015880 : Zinc finger, C2H2-like |
| MGG13773.6 | 2.70 | indoleamine 2,3-dioxygenase family protein | IPR000898 : Indoleamine 2,3-dioxygenase |
| MGG06326.6 | 2.70 | vacuolar ATP synthase 16 kDa proteolipid subunit | IPR002379 : ATPase, F0/V0 complex, subunit C, IPR011555 : ATPase, V0 complex, proteolipid subunit C, eukaryotic |
| MGG10309.6 | 2.68 | sugar transporter | IPR005828 : General substrate transporter, IPR007114 : , IPR016196 : Major facilitator superfamily, general substrate transporter |
| MGG08613.6 | 2.67 | poly polymerase 2 ADP-ribosyltransferase 2) | IPR001357 : BRCT, IPR004102 : Poly(ADP-ribose) polymerase, regulatory region, IPR008893 : WGR,IPR008893 : WGR |
| MGG00545.6 | 2.67 | hypothetical protein | No defined Interpro term |
| MGG02289.6 | 2.67 | conserved hypothetical protein | No defined Interpro term |
| MGG00015.6 | 2.67 | catechol O-methyltransferase | IPR002935 : O-methyltransferase, family 3 |
| MGG05751.6 | 2.67 | conserved hypothetical protein | No defined Interpro term |
| MGG08258.6 | 2.67 | peptide transporter PTR2 | IPR000109 : TGF-beta receptor, type I/II extracellular region, IPR016196 : Major facilitator superfamily, general substrate transporter |
| MGG08574.6 | 2.66 | conserved hypothetical protein | No defined Interpro term |
| MGG00428.6 | 2.66 | conidial yellow pigment biosynthesis polyketide synthase | IPR000794 : Beta-ketoacyl synthase,IPR001031 : Thioesterase, IPR001227 : Acyl transferase region, IPR006163 : Phosphopantetheine-binding |
| MGG00494.6 | 2.65 | transcriptional regulatory protein | IPR001138 : Fungal transcriptional regulatory protein, N-terminal |
| MGG05164.6 | 2.65 | conserved hypothetical protein | No defined Interpro term |
| MGG09363.6 | 2.65 | conserved hypothetical protein | No defined Interpro term |
| MGG05103.6 | 2.64 | conserved hypothetical protein | No defined Interpro term |
| MGG01728.6 | 2.64 | methylenetetrahydrofolate reductase 1 | IPR003171 : Methylenetetrahydrofolate reductase, IPR004621 : Eukaryotic-type methylenetetrahydrofolate reductase |
| MGG09332.6 | 2.63 | hypothetical protein | No defined Interpro term |
| MGG07376.6 | 2.63 | hypothetical protein | No defined Interpro term |
| MGG03341.6 | 2.63 | galactose-proton symporter | IPR005828 : General substrate transporter, IPR016196 : Major facilitator superfamily, general substrate transporter |
| MGG03442.6 | 2.62 | conserved hypothetical protein | No defined Interpro term |
| MGG08854.6 | 2.61 | conserved hypothetical protein | No defined Interpro term |
| MGG08495.6 | 2.61 | conserved hypothetical protein | IPR010730 : Heterokaryon incompatibility |
| MGG06923.6 | 2.61 | myosin-2 | IPR000048 : IQ calmodulin-binding region, IPR001609 : Myosin head, motor region |
| MGG06932.6 | 2.60 | conserved hypothetical protein | No defined Interpro term |
| MGG13716.6 | 2.60 | hypothetical protein | No defined Interpro term |
| MGG09458.6 | 2.59 | GTP cyclohydrolase I | IPR001474 : GTP cyclohydrolase I |
| MGG08583.6 | 2.59 | beta-glucosidase 1 precursor | IPR001764 : Glycoside hydrolase, family 3, N-terminal, IPR002772 : Glycoside hydrolase, family 3, C-terminal |
| MGG11174.6 | 2.57 | sensor protein gacS | IPR001789 : Signal transduction response regulator, receiver region, IPR004358 : Signal transduction histidine kinase-related protein, C-terminal, |
| MGG09555.6 | 2.57 | double-strand break repair protein mus-23 | IPR003701 : DNA repair exonuclease, IPR004843 : Metallophosphoesterase, IPR007281 : Mre11, DNA-binding |
| MGG10969.6 | 2.56 | kynureninase | IPR010111 : Kynureninase, IPR015421 : Pyridoxal phosphate-dependent transferase, major region, subdomain 1 |
| MGG12301.6 | 2.56 | translation initiation factor 4E | IPR001040 : Eukaryotic translation initiation factor 4E (eIF-4E) |
| MGG10548.6 | 2.56 | conserved hypothetical protein | No defined Interpro term |
| MGG00746.6 | 2.56 | conserved hypothetical protein | No defined Interpro term |
| MGG05787.6 | 2.56 | conserved hypothetical protein | IPR001128 : Cytochrome P450P450 |
| MGG01290.6 | 2.54 | nicotinamide mononucleotide adenylyltransferase 1 | IPR004820 : Cytidylyltransferase, IPR005248 : nicotinate-nucleotide adenylyltransferase |
| MGG07604.6 | 2.54 | conserved hypothetical protein | No defined Interpro term |
| MGG00393.6 | 2.53 | leucine-rich repeat-containing protein 40 | IPR001611 : Leucine-rich repeat |
| MGG10738.6 | 2.53 | mitochondrial chaperone BCS1 | IPR003593 : ATPase, AAA+ type, core |
| MGG06580.6 | 2.53 | FAD binding domain-containing protein | IPR006094 : FAD linked oxidase, N-terminal |
| MGG10274.6 | 2.53 | aflatoxin biosynthesis ketoreductase nor-1 | IPR002198 : Short-chain dehydrogenase/reductase SDR |
| MGG10048.6 | 2.52 | conserved hypothetical protein | No defined Interpro term |
| MGG12963.6 | 2.52 | conserved hypothetical protein | IPR000719 : Protein kinase, core |
| MGG02904.6 | 2.51 | conserved hypothetical protein | No defined Interpro term |
| MGG03664.6 | 2.51 | palmitoyltransferase ERF2 |  |
| MGG05331.6 | 2.51 | protein-S-isoprenylcysteine O-methyltransferase | IPR007269 : Isoprenylcysteine carboxyl methyltransferase |
| MGG00282.6 | 2.51 | conserved hypothetical protein | IPR000209 : Peptidase S8 and S53, subtilisin, kexin, sedolisin, IPR010435 : Peptidase S8A, DUF1034 C-terminal |
| MGG07703.6 | 2.50 | hypothetical protein | No defined Interpro term |
| MGG15388.6 | 2.50 | lactose permease | IPR003663 : Sugar/inositol transporter, IPR005828 : General substrate transporter |
| MGG02793.6 | 2.50 | mannan endo-1,6-alpha-mannosidase DCW1 | IPR005198 : Glycoside hydrolase, family 76, IPR008928 : Six-hairpin glycosidase-like |
| MGG03128.6 | 2.49 | conserved hypothetical protein | No defined Interpro term |
| MGG02530.6 | 2.49 | quinate permease | IPR003663 : Sugar/inositol transporter, IPR005828 : General substrate transporter, IPR005829 : Sugar transporter, conserved site |
| MGG04206.6 | 2.49 | conserved hypothetical protein | No defined Interpro term |
| MGG04071.6 | 2.48 | hypothetical protein | No defined Interpro term |
| MGG12043.6 | 2.48 | hypothetical protein | No defined Interpro term |
| MGG04544.6 | 2.47 | F1F0 ATP synthase assembly protein Atp11 | IPR010591 : ATP11 |
| MGG10490.6 | 2.47 | GPI-anchor transamidase | IPR001096 : Peptidase C13, legumain |
| MGG06849.6 | 2.46 | amidohydrolase 3 | IPR013108 : Amidohydrolase 3 |
| MGG03716.6 | 2.46 | phosphatidylinositol N-acetylglucosaminyltransferase subunit C | IPR009450 : Phosphatidylinositol N-acetylglucosaminyltransferase |
| MGG06113.6 | 2.46 | conserved hypothetical protein | No defined Interpro term |
| MGG05746.6 | 2.45 | canalicular multispecific organic anion transporter 1 | IPR001140 : ABC transporter, transmembrane region IPR003593 : ATPase, AAA+ type, core |
| MGG10586.6 | 2.45 | conserved hypothetical protein | IPR000223 : Peptidase S26A, signal peptidase I |
| MGG13924.6 | 2.45 | serine palmitoyltransferase 2 | IPR001917 : Aminotransferase, class-II, pyridoxal-phosphate binding site, IPR015421 : Pyridoxal phosphate-dependent transferase, major region, subdomain 1 |
| MGG00806.6 | 2.45 | polyketide synthase | IPR000794 : Beta-ketoacyl synthase, IPR001227 : Acyl transferase region, IPR006163 : Phosphopantetheine-binding |
| MGG02916.6 | 2.45 | hypothetical protein | No defined Interpro term |
| MGG11754.6 | 2.45 | heavy metal tolerance protein | IPR003593 : ATPase, AAA+ type, core, IPR011527 : ABC transporter, transmembrane region, type 1, IPR017871 : ABC transporter, conserved site |
| MGG02988.6 | 2.45 | conserved hypothetical protein | No defined Interpro term |
| MGG06722.6 | 2.45 | 1,3-beta-glucanosyltransferase gel2 | IPR004886 : Glycolipid anchored surface protein GAS1, IPR013781 : Glycoside hydrolase, subgroup, catalytic core |
| MGG04404.6 | 2.44 | pisatin demethylase | IPR001128 : Cytochrome P450 |
| MGG08646.6 | 2.44 | DNA replication licensing factor mcm5 | IPR001208 : DNA-dependent ATPase MCM, IPR003593 : ATPase, AAA+ type, core, IPR008048 : MCM protein 5 |
| MGG11383.6 | 2.44 | phosphoacetylglucosamine mutase | IPR005843 : Alpha-D-phosphohexomutase, C-terminal, IPR005844 : Alpha-D-phosphohexomutase, alpha/beta/alpha domain I |
| MGG06873.6 | 2.44 | conserved hypothetical protein |  |
| MGG01977.6 | 2.44 | conserved hypothetical protein | No defined Interpro term |
| MGG00016.6 | 2.44 | 2-epi-5-epi-valiolone synthase | IPR002658 : 3-dehydroquinate synthase AroB |
| MGG12759.6 | 2.44 | conserved hypothetical protein | IPR006913 : Glutathione-dependent formaldehyde-activating, GFA |
| MGG06508.6 | 2.43 | thioredoxin | IPR001853 : DSBA oxidoreductase, IPR012335 : Thioredoxin fold |
| MGG04915.6 | 2.43 | hypothetical protein | No defined Interpro term |
| MGG04185.6 | 2.43 | conserved hypothetical protein | IPR000357 : HEAT, IPR016024 : Armadillo-type fold |
| MGG04706.6 | 2.42 | hypothetical protein | No defined Interpro term |
| MGG01412.6 | 2.42 | conserved hypothetical protein | IPR001810 : Cyclin-like F-box |
| MGG02514.6 | 2.42 | ankyrin repeat protein nuc-2 | IPR002110 : Ankyrin, IPR004331 : SPX, N-terminal, IPR017946 : PLC-like phosphodiesterase, TIM beta/alpha-barrel domain, |
| MGG13895.6 | 2.42 | fructose-bisphosphate aldolase | IPR000771 : Ketose-bisphosphate aldolase, class-II, IPR013785 : Aldolase-type TIM barrel |
| MGG07243.6 | 2.41 | conserved hypothetical protein | IPR001164 : Arf GTPase activating protein, IPR015940 : Ubiquitin-associated/translation elongation factor EF1B, N-terminal, eukaryote |
| MGG02084.6 | 2.41 | glyoxylate reductase | IPR006140 : D-isomer specific 2-hydroxyacid dehydrogenase, NAD-binding |
| MGG04816.6 | 2.40 | conserved hypothetical protein | IPR000504 : RNA recognition motif, RNP-1, IPR012677 : Nucleotide-binding, alpha-beta plait |
| MGG07238.6 | 2.40 | DNA polymerase kappa | IPR001126 : DNA-repair protein, UmuC-like, IPR006642 : Zinc finger, Rad18-type putative |
| MGG02899.6 | 2.40 | proline-specific permease | IPR002293 : Amino acid/polyamine transporter I, IPR004841 : Amino acid permease-associated region |
| MGG00040.6 | 2.38 | high-affinity glucose transporter ght2 | IPR003663 : Sugar/inositol transporter, IPR005828 : General substrate transporter, IPR005829 : Sugar transporter, conserved site |
| MGG05007.6 | 2.38 | conserved hypothetical protein | No defined Interpro term |
| MGG03967.6 | 2.37 | hypothetical protein | IPR002110 : Ankyrin |
| MGG00202.6 | 2.37 | conserved hypothetical protein | IPR001910 : Inosine/uridine-preferring nucleoside hydrolase |
| MGG08639.6 | 2.37 | hypothetical protein | IPR007727 : Spo12 |
| MGG00082.6 | 2.37 | conserved hypothetical protein | IPR010730 : Heterokaryon incompatibility |
| MGG03133.6 | 2.36 | conserved hypothetical protein | IPR007087 : Zinc finger, C2H2-type, IPR013087 : Zinc finger, C2H2-type/integrase, DNA-binding, IPR015880 : Zinc finger, C2H2-like |
| MGG01012.6 | 2.36 | ISWI chromatin-remodeling complex ATPase ISW2 | IPR001005 : SANT, DNA-binding, IPR001650 : DNA/RNA helicase, C-terminal, IPR014021 : Helicase, superfamily 1 and 2, ATP-binding, IPR014778 : Myb, DNA-binding, IPR015194 : ATPase, nucleosome remodelling ISWI, HAND domain |
| MGG00705.6 | 2.36 | hypothetical protein | No defined Interpro term |
| MGG12506.6 | 2.36 | hypothetical protein | No defined Interpro term |
| MGG04538.6 | 2.35 | conserved hypothetical protein | No defined Interpro term |
| MGG06575.6 | 2.35 | conserved hypothetical protein | IPR007087 : Zinc finger, C2H2-type, IPR015880 : Zinc finger, C2H2-like |
| MGG00450.6 | 2.34 | phosphoenolpyruvate carboxykinase |  |
| MGG00795.6 | 2.34 | conserved hypothetical protein | IPR002110 : Ankyrin |
| MGG06209.6 | 2.34 | inorganic phosphate transport protein PHO88 | IPR012098 : Inorganic phosphate transport PHO88 |
| MGG05554.6 | 2.34 | conserved hypothetical protein | IPR000169 : Peptidase, cysteine peptidase active site, PR002085 : Alcohol dehydrogenase superfamily, zinc-containing, IPR016040 : NAD(P)-binding |
| MGG03724.6 | 2.33 | copper-transporting ATPase RAN1 | IPR000150 : Cof protein, IPR001757 : ATPase, P-type, K/Mg/Cd/Cu/Zn/Na/Ca/Na/H-transporter, IPR001877 : ATPase 1, copper-transporting, IPR006121 : Heavy metal transport/detoxification protein, |
| MGG06714.6 | 2.33 | conserved hypothetical protein | No defined Interpro term |
| MGG00312.6 | 2.33 | glyoxylate reductase | IPR006140 : D-isomer specific 2-hydroxyacid dehydrogenase, NAD-binding |
| MGG06654.6 | 2.32 | conserved hypothetical protein | IPR001199 : Cytochrome b5 |
| MGG02069.6 | 2.32 | glyoxalase/bleomycin resistance protein/dioxygenase | IPR004360 : Glyoxalase/bleomycin resistance protein/dioxygenase |
| MGG06593.6 | 2.32 | endo-1,4-beta-xylanase 2 | IPR001137 : Glycoside hydrolase, family 11, IPR008985 : Concanavalin A-like lectin/glucanase, IPR013319 : Glycoside hydrolase, families 11 and 12, catalytic core |
| MGG15028.6 | 2.32 | hypothetical protein | No defined Interpro term |
| MGG13427.6 | 2.31 | NAD/NADP octopine/nopaline dehydrogenase | IPR003421 : Opine dehydrogenase, IPR011128 : NAD-dependent glycerol-3-phosphate dehydrogenase, N-terminal, IPR013328 : Dehydrogenase, multihelical, IPR016040 : NAD(P)-binding |
| MGG02626.6 | 2.31 | septin | IPR000038 : Cell division/GTP binding protein |
| MGG15036.6 | 2.31 | conserved hypothetical protein | No defined Interpro term |
| MGG02818.6 | 2.31 | isoamyl alcohol oxidase | IPR006094 : FAD linked oxidase, N-terminal, IPR012951 : Berberine/berberine-like, IPR016166 : FAD-binding, type 2 |
| MGG08750.6 | 2.30 | conserved hypothetical protein | No defined Interpro term |
| MGG10360.6 | 2.29 | haloacid dehalogenase | IPR006328 : Haloacid dehalogenase, type II, IPR006388 : HAD-superfamily hydrolase, subfamily IA, variant 2 |
| MGG15037.6 | 2.29 | hypothetical protein | No defined Interpro term |
| MGG04736.6 | 2.28 | conserved hypothetical protein | IPR016040 : NAD(P)-binding |
| MGG03123.6 | 2.28 | multidrug and toxin extrusion protein 1 | IPR002528 : Multi antimicrobial extrusion protein MatE |
| MGG10639.6 | 2.27 | hypothetical protein | No defined Interpro term |
| MGG08187.6 | 2.27 | thiol-specific monooxygenase | IPR000759 : Adrenodoxin reductase, IPR013027 : FAD-dependent pyridine nucleotide-disulphide oxidoreductase |
| MGG10653.6 | 2.27 | eukaryotic translation initiation factor 3 subunit F | IPR000555 : Mov34/MPN/PAD-1 |
| MGG07833.6 | 2.27 | conserved hypothetical protein | IPR013112 : FAD-binding 8, IPR013121 : Ferric reductase, NAD binding, IPR017927 : Ferredoxin reductase-type FAD-binding domain |
| MGG09858.6 | 2.26 | hypothetical protein | No defined Interpro term |
| MGG00836.6 | 2.25 | NUDIX domain-containing protein | IPR000086 : NUDIX hydrolase, core, IPR015797 : NUDIX |
| MGG08654.6 | 2.25 | conserved hypothetical protein | No defined Interpro term |
| MGG13767.6 | 2.25 | lovastatin nonaketide synthase | IPR000794 : Beta-ketoacyl synthase, IPR001227 : Acyl transferase region, IPR002364 : Quinone oxidoreductase/zeta-crystallin, conserved site, IPR006163 : Phosphopantetheine-binding, IPR013217 : Methyltransferase type 12, IPR013968 : Polyketide synthase, KR |
| MGG09089.6 | 2.25 | conserved hypothetical protein | IPR000182 : GCN5-related N-acetyltransferase, IPR016181 : Acyl-CoA N-acyltransferase |
| MGG10956.6 | 2.25 | hypothetical protein | No defined Interpro term |
| MGG03070.6 | 2.24 | epoxide hydrolase domain-containing protein | IPR000073 : Alpha/beta hydrolase fold-1, IPR016292 : Epoxide hydrolase |
| MGG01601.6 | 2.24 | hypothetical protein | No defined Interpro term |
| MGG13781.6 | 2.24 | serine hydroxymethyltransferase | IPR001085 : Glycine hydroxymethyltransferase, IPR015421 : Pyridoxal phosphate-dependent transferase, major region, subdomain 1 |
| MGG05531.6 | 2.23 | hypothetical protein | IPR008427 : Extracellular membrane protein, 8-cysteine region, CFEM |
| MGG07349.6 | 2.23 | hypothetical protein | No defined Interpro term |
| MGG13169.6 | 2.23 | conserved hypothetical protein | IPR011257 : DNA glycosylase |
| MGG12929.6 | 2.22 | glyoxylate reductase | IPR006139 : D-isomer specific 2-hydroxyacid dehydrogenase, catalytic region |
| MGG05008.6 | 2.21 | aldehyde dehydrogenase | IPR015590 : Aldehyde dehydrogenase, IPR016160 : Aldehyde dehydrogenase, conserved site |
| MGG00820.6 | 2.21 | conserved hypothetical protein | IPR010730 : Heterokaryon incompatibility |
| MGG04951.6 | 2.20 | Fungal transcriptional factor | IPR001138 : Fungal transcriptional regulatory protein, N-terminal |
| MGG01606.6 | 2.20 | methylmalonate-semialdehyde dehydrogenase, mitochondrial precursor | IPR010061 : Methylmalonate-semialdehyde dehydrogenase, IPR015590 : Aldehyde dehydrogenase |
| MGG08950.6 | 2.20 | high-affinity glucose transporter | IPR005828 : General substrate transporter, IPR016196 : Major facilitator superfamily, general substrate transporter |
| MGG07430.6 | 2.19 | conserved hypothetical protein | IPR000215 : Protease inhibitor I4, serpin |
| MGG14586.6 | 2.19 | conserved hypothetical protein | IPR001138 : Fungal transcriptional regulatory protein, N-terminal, IPR007219 : Fungal specific transcription factor |
| MGG07261.6 | 2.19 | 2-nitropropane dioxygenase | IPR004136 : 2-nitropropane dioxygenase, NPD |
| MGG04019.6 | 2.18 | transcription factor tau subunit sfc1 | No defined Interpro term |
| MGG10253.6 | 2.18 | conserved hypothetical protein | IPR010699 : Protein of unknown function DUF1275 |
| MGG07646.6 | 2.17 | alpha-glucuronidase | IPR005154 : Glycoside hydrolase family 67, IPR011099 : Glycosyl hydrolase 67, C-terminal |
| MGG04442.6 | 2.16 | splicing factor U2AF-associated protein 2 | IPR000504 : RNA recognition motif, RNP-1, IPR012677 : Nucleotide-binding, alpha-beta plait |
| MGG00074.6 | 2.16 | conserved hypothetical protein | IPR008960 : Carbohydrate-binding family 9/cellobiose dehydrogenase, cytochrome |
| MGG03399.6 | 2.16 | arsenite resistance protein arsB | IPR002657 : Bile acid:sodium symporter, IPR004706 : Arsenical-resistance protein ACR3 |
| MGG03202.6 | 2.15 | replication factor C subunit 3 | IPR003593 : ATPase, AAA+ type, core, IPR008921 : DNA polymerase III clamp loader subunit, C-terminal |
| MGG03141.6 | 2.15 | hypothetical protein | No defined Interpro term |
| MGG11274.6 | 2.15 | conserved hypothetical protein | IPR002227 : Tyrosinase, IPR008922 : Di-copper centre-containing |
| MGG04961.6 | 2.14 | conserved hypothetical protein | No defined Interpro term |
| MGG06552.6 | 2.14 | monoxygenase | IPR003042 : Aromatic-ring hydroxylase-like, IPR013027 : FAD-dependent pyridine nucleotide-disulphide oxidoreductase |
| MGG09906.6 | 2.14 | mitochondrial phosphate carrier protein 2 | IPR001993 : Mitochondrial substrate carrier |
| MGG02091.6 | 2.13 | hypothetical protein | No defined Interpro term |
| MGG01429.6 | 2.13 | conserved hypothetical protein | IPR011513 : Nse1 non-SMC component of SMC5-6 complex, IPR013083 : Zinc finger, RING/FYVE/PHD-type |
| MGG00418.6 | 2.12 | hypothetical protein | No defined Interpro term |
| MGG11124.6 | 2.12 | Na(+)/H(+) antiporter | IPR006153 : Cation/H+ exchanger |
| MGG02807.6 | 2.12 | RNAse P Rpr2/Rpp21 subunit domain-containing protein | IPR007175 : RNAse P, Rpr2/Rpp21 subunit |
| MGG02789.6 | 2.12 | conserved hypothetical protein | No defined Interpro term |
| MGG04322.6 | 2.12 | hypothetical protein | No defined Interpro term |
| MGG07116.6 | 2.12 | cell morphogenesis protein PAG1 | IPR001664 : Intermediate filament protein, IPR016024 : Armadillo-type fold |
| MGG12267.6 | 2.12 | FAD synthetase | IPR002500 : Phosphoadenosine phosphosulphate reductase |
| MGG09215.6 | 2.11 | conserved hypothetical protein | No defined Interpro term |
| MGG04435.6 | 2.11 | bifunctional purine biosynthesis protein ADE17 | IPR002695 : AICARFT/IMPCHase bienzyme, IPR011607 : MGS-like, IPR013982 : AICARFT/IMPCHase bienzyme, formylation region |
| MGG01389.6 | 2.11 | alpha-N-arabinofuranosidase | IPR010720 : Alpha-L-arabinofuranosidase, C-terminal, IPR017853 : Glycoside hydrolase, catalytic core |
| MGG09883.6 | 2.11 | conserved hypothetical protein | No defined Interpro term |
| MGG09210.6 | 2.11 | endosome protein | IPR001870 : B302 (SPRY)-like, IPR003877 : SPla/RYanodine receptor SPRY |
| MGG14805.6 | 2.10 | DUF907 domain-containing protein | IPR010308 : Protein of unknown function DUF907, fungi |
| MGG06980.6 | 2.10 | conserved hypothetical protein | IPR011701 : Major facilitator superfamily MFS-1, IPR016196 : Major facilitator superfamily, general substrate transporter |
| MGG05141.6 | 2.10 | conserved hypothetical protein | No defined Interpro term |
| MGG08895.6 | 2.09 | fructose-1,6-bisphosphatase | IPR000146 : Fructose-1,6-bisphosphatase |
| MGG04110.6 | 2.09 | queuine tRNA-ribosyltransferase | IPR002616 : Queuine/other tRNA-ribosyltransferase, IPR004803 : Queuine tRNA-ribosyltransferase |
| MGG03651.6 | 2.09 | conserved hypothetical protein | IPR011333 : BTB/POZ fold, IPR013069 : BTB/POZ, IPR013089 : Kelch related |
| MGG12678.6 | 2.09 | glucan endo-1,3-alpha-glucosidase agn1 | IPR005197 : Glycoside hydrolase, family 71 |
| MGG15073.6 | 2.09 | conserved hypothetical protein | No defined Interpro term |
| MGG06548.6 | 2.08 | carotenoid oxygenase | IPR004294 : Carotenoid oxygenase |
| MGG05879.6 | 2.08 | conserved hypothetical protein | IPR001087 : Lipase, GDSL, IPR013830 : Esterase, SGNH hydrolase-type, IPR013831 : Esterase, SGNH hydrolase-type, subgroup |
| MGG04888.6 | 2.08 | MFS hexose transporter | IPR005828 : General substrate transporter, IPR005829 : Sugar transporter, conserved site |
| MGG04914.6 | 2.07 | conserved hypothetical protein | IPR002110 : Ankyrin |
| MGG06014.6 | 2.07 | DNA base excision repair N-glycosylase 1 | IPR000445 : Helix-hairpin-helix motif, IPR003265 : HhH-GPD domain, IPR011257 : DNA glycosylase |
| MGG12636.6 | 2.07 | mediator of RNA polymerase II transcription subunit 17 | No defined Interpro term |
| MGG05989.6 | 2.07 | dipeptidyl aminopeptidase B | IPR001375 : Peptidase S9, prolyl oligopeptidase active site region, IPR002469 : Peptidase S9B, dipeptidylpeptidase IV N-terminal |
| MGG14810.6 | 2.06 | hypothetical protein | No defined Interpro term |
| MGG00287.6 | 2.06 | hypothetical protein | No defined Interpro term |
| MGG08774.6 | 2.06 | chitin deacetylase | IPR002509 : Polysaccharide deacetylase, IPR011330 : Glycoside hydrolase/deacetylase, beta/alpha-barrel |
| MGG05608.6 | 2.06 | conserved hypothetical protein | No defined Interpro term |
| MGG03161.6 | 2.05 | conserved hypothetical protein | IPR013916 : , IPR014751 : DNA double-strand break repair and VJ recombination XRCC4, C-terminal |
| MGG00556.6 | 2.05 | hypothetical protein | No defined Interpro term |
| MGG04516.6 | 2.05 | glycosyl transferase family 17 protein | IPR006813 : Glycosyl transferase, family 17 |
| MGG05123.6 | 2.05 | conserved hypothetical protein | No defined Interpro term |
| MGG05255.6 | 2.05 | autophagy protein 16 | IPR013923 : Autophagy protein 16 |
| MGG01717.6 | 2.04 | conserved hypothetical protein | No defined Interpro term |
| MGG02510.6 | 2.04 | coiled-coil domain-containing protein 25 | IPR008532 : Protein of unknown function DUF814 |
| MGG14577.6 | 2.04 | hypothetical protein | No defined Interpro term |
| MGG00631.6 | 2.04 | conserved hypothetical protein | No defined Interpro term |
| MGG08007.6 | 2.03 | conserved hypothetical protein | No defined Interpro term |
| MGG01759.6 | 2.03 | DNA replication licensing factor mcm3 | IPR001208 : DNA-dependent ATPase MCM, IPR003593 : ATPase, AAA+ type, core, IPR012340 : Nucleic acid-binding, OB-fold |
| MGG11615.6 | 2.03 | hypothetical protein | No defined Interpro term |
| MGG05767.6 | 2.03 | conserved hypothetical protein | No defined Interpro term |
| MGG11724.6 | 2.03 | hypothetical protein | No defined Interpro term |
| MGG03362.6 | 2.02 | conserved hypothetical protein | IPR002656 : Acyltransferase 3 |
| MGG05979.6 | 2.02 | conserved hypothetical protein | IPR011701 : Major facilitator superfamily MFS-1, IPR016196 : Major facilitator superfamily, general substrate transporter |
| MGG07609.6 | 2.02 | hypothetical protein | No defined Interpro term |
| MGG04153.6 | 2.02 | multidrug transporter of the major facilitator superfamily | IPR007114 : , IPR011701 : Major facilitator superfamily MFS-1, IPR016196 : Major facilitator superfamily, general substrate transporter |
| MGG05397.6 | 2.01 | protein kinase domain protein | IPR000719 : Protein kinase, core, IPR002290 : Serine/threonine protein kinase |
| MGG06896.6 | 2.01 | UDP-N-acetylglucosamine-peptide N-acetylglucosaminyltransferase | IPR001440 : Tetratricopeptide TPR |
| MGG01372.6 | 2.01 | conserved hypothetical protein | IPR017867 : Protein-tyrosine phosphatase |
| MGG01292.6 | 2.01 | nucleolar protein NOP2 | IPR001678 : Bacterial Fmu (Sun)/eukaryotic nucleolar NOL1/Nop2p |

^a^ Induction ratios from the microarray analysis are calculated as the expression in the *ΔMohox2* mutant divided by the wild-type during conidiation.
