# Supplementary material for: Gene Expression Profiling during Conidiation in the Rice Blast Pathogen Magnaporthe oryzae
Source: PLoS One. 2012 Aug 21;7(8):e43202. doi: 10.1371/journal.pone.0043202 (PMC3424150; doi:10.1371/journal.pone.0043202)
Supplement: Table S7 — Genes induced in the wild type and repressed in the MoHOX2 deletion mutant during conidiation. (DOCX) [file pone.0043202.s007.docx]

**Table S7.** Genes induced in the wild type and repressed in the *MoHOX2* deletion mutant during conidiation

| **Locus** | **Fold-induction during conidiation in the wild-type^a^** | **Fold-reduction during conidiation in the *ΔMohox2* mutant^b^** | **Annotation** | **Interpro search** |
| --- | --- | --- | --- | --- |
| MGG01046.6 | 17.42 | 0.02 | methionyl-tRNA synthetase | IPR002304 : Methionyl-tRNA synthetase, class Ia, IPR009080: Aminoacyl-tRNA synthetase, class 1a |
| MGG13137.6 | 15.54 | 0.04 | ABC1 family protein | IPR004147: ABC-1, IPR011009: Protein kinase-like |
| MGG06446.6 | 22.74 | 0.04 | 5-aminolevulinate synthase, mitochondrial precursor | IPR001917: Aminotransferase, class-II, pyridoxal-phosphate binding site |
| MGG07997.6 | 25.42 | 0.04 | covalently-linked cell wall protein | IPR000420: Yeast PIR protein repeat |
| MGG10197.6 | 28.6 | 0.04 | conserved hypothetical protein | IPR000054: Ribosomal protein L31e, IPR007219: Fungal specific transcription factor |
| MGG00888.6 | 5.91 | 0.05 | hypothetical protein | No defined Interpro term |
| MGG09863.6 | 6.79 | 0.05 | conserved hypothetical protein | No defined Interpro term |
| MGG10031.6 | 8.66 | 0.06 | hypothetical protein | No defined Interpro term |
| MGG11271.6 | 10.13 | 0.06 | hypothetical protein | No defined Interpro term |
| MGG08758.6 | 10.81 | 0.06 | aminopeptidase Y | IPR003137: Protease-associated PA, IPR007484: Peptidase M28 |
| MGG05908.6 | 13.71 | 0.06 | cytochrome P450 52A11 | IPR001128: Cytochrome P450, IPR002401: Cytochrome P450, E-class, group I |
| MGG07623.6 | 17.67 | 0.06 | hypothetical protein | IPR001002: Chitin-binding, type 1 |
| MGG05805.6 | 19.49 | 0.07 | hypothetical protein | No defined Interpro term |
| MGG15378.6 | 23.16 | 0.07 | hypothetical protein | No defined Interpro term |
| MGG07565.6 | 27.65 | 0.07 | conserved hypothetical protein | IPR008427: Extracellular membrane protein, 8-cysteine region, CFEM, |
| MGG02840.6 | 35.33 | 0.07 | conserved hypothetical protein | IPR011701: Major facilitator superfamily MFS-1, IPR016196: Major facilitator superfamily, general substrate transporter |
| MGG12522.6 | 10.56 | 0.08 | hypothetical protein | No defined Interpro term |
| MGG05035.6 | 10.97 | 0.08 | hypothetical protein | No defined Interpro term |
| MGG02837.6 | 13.62 | 0.08 | conserved hypothetical protein | No defined Interpro term |
| MGG10107.6 | 21.09 | 0.08 | caleosin domain-containing protein | IPR007736: Caleosin related |
| MGG05109.6 | 23.35 | 0.08 | conserved hypothetical protein | No defined Interpro term |
| MGG02962.6 | 4.56 | 0.09 | C6 zinc finger domain-containing protein | IPR001138: Fungal transcriptional regulatory protein, N-terminal |
| MGG12146.6 | 4.6 | 0.09 | ER membrane protein | IPR013635: ICE2 |
| MGG01485.6 | 7.53 | 0.09 | conserved hypothetical protein | IPR011701: Major facilitator superfamily MFS-1, IPR016196: Major facilitator superfamily, general substrate transporter |
| MGG00748.6 | 12.37 | 0.09 | myosin-5 | IPR001609: Myosin head, motor region, IPR000048: IQ calmodulin-binding region, IPR001452: Src homology-3 domain, IPR010926: Myosin tail 2 |
| MGG03403.6 | 14.37 | 0.09 | hypothetical protein | No defined Interpro term |
| MGG13535.6 | 4.03 | 0.1 | nucleoporin POM152 | No defined Interpro term |
| MGG15343.6 | 5.22 | 0.1 | hypothetical protein | No defined Interpro term |
| MGG02329.6 | 5.87 | 0.10 | isotrichodermin C-15 hydroxylase | IPR001128: Cytochrome P450, IPR001209 : Ribosomal protein S14 |
| MGG08429.6 | 6.19 | 0.1 | serin endopeptidas | IPR000209: Peptidase S8 and S53, subtilisin, kexin, sedolisin, IPR003137 : Protease-associated PA |
| MGG07150.6 | 9.62 | 0.1 | conserved hypothetical protein | No defined Interpro term |
| MGG05584.6 | 13.71 | 0.1 | conserved hypothetical protein | IPR004854: Ubiquitin fusion degradation protein UFD1 |
| MGG10571.6 | 21.01 | 0.1 | conserved hypothetical protein | No defined Interpro term |
| MGG11816.6 | 8.24 | 0.11 | NADPH-dependent 1-acyldihydroxyacetone phosphate reductase | IPR002198: Short-chain dehydrogenase/reductase SDR, IPR002347: Glucose/ribitol dehydrogenase, IPR016040: NAD(P)-binding |
| MGG02294.6 | 5.11 | 0.12 | ent-kaurene oxidase | IPR001128: Cytochrome P450 |
| MGG02817.6 | 5.42 | 0.12 | glutamate decarboxylase | IPR010107 : Glutamate decarboxylase, IPR000760: Inositol monophosphatase, IPR002129: Pyridoxal phosphate-dependent decarboxylase |
| MGG04378.6 | 16.25 | 0.12 | integral membrane protein | No defined Interpro term |
| MGG12421.6 | 17.55 | 0.12 | aminomethyltransferase | IPR006076: FAD dependent oxidoreductase, IPR006222: Glycine cleavage T-protein, N-terminal |
| MGG00334.6 | 18.74 | 0.12 | hypothetical protein | No defined Interpro term |
| MGG04346.6 | 20.26 | 0.12 | sterol 24-C-methyltransferase | IPR013216: Methyltransferase type 11, IPR013705: Sterol methyltransferase C-terminal |
| MGG03896.6 | 4.53 | 0.13 | conserved hypothetical protein | No defined Interpro term |
| MGG08161.6 | 4.67 | 0.13 | conserved hypothetical protein | IPR013217: Methyltransferase type 12 |
| MGG05100.6 | 6.62 | 0.13 | hypothetical protein | IPR001283: Allergen V5/Tpx-1 related, IPR014044: SCP-like extracellular |
| MGG02612.6 | 11.14 | 0.13 | 3-oxoacyl-[acyl-carrier-protein] reductase | IPR002198: Short-chain dehydrogenase/reductase SDR, IPR016040: NAD(P)-binding |
| MGG08846.6 | 11.45 | 0.13 | conserved hypothetical protein | IPR000759: Adrenodoxin reductase, IPR013027: FAD-dependent pyridine nucleotide-disulphide oxidoreductase |
| MGG01391.6 | 4.87 | 0.14 | ent-kaurene oxidase | IPR001128: Cytochrome P450, IPR002403: Cytochrome P450, E-class, group IV |
| MGG02109.6 | 6.63 | 0.14 | conserved hypothetical protein | No defined Interpro term |
| MGG12552.6 | 7.73 | 0.14 | hypothetical protein | No defined Interpro term |
| MGG10710.6 | 8.71 | 0.14 | oxidoreductase | IPR003042: Aromatic-ring hydroxylase-like, IPR006076 : FAD dependent oxidoreductase |
| MGG00635.6 | 11.51 | 0.14 | conserved hypothetical protein | No defined Interpro term |
| MGG07311.6 | 12.48 | 0.14 | hypothetical protein | No defined Interpro term |
| MGG12988.6 | 12.98 | 0.14 | alpha-glucoside transport protein | IPR003663: Sugar/inositol transporter, IPR016196: Major facilitator superfamily, general substrate transporter |
| MGG04487.6 | 5.13 | 0.15 | conserved hypothetical protein | No defined Interpro term |
| MGG01933.6 | 5.29 | 0.15 | conserved hypothetical protein | No defined Interpro term |
| MGG06572.6 | 5.3 | 0.15 | phosphatidylinositol-4-phosphate 5-kinase its3 (PtdIns(4)P-5-kinase) | IPR002498: Phosphatidylinositol-4-phosphate 5-kinase |
| MGG07198.6 | 5.33 | 0.15 | conserved hypothetical protein | IPR001680: WD40 repeat, IPR015943: WD40/YVTN repeat-like |
| MGG06225.6 | 6.38 | 0.15 | hypothetical protein | No defined Interpro term |
| MGG10214.6 | 13.08 | 0.15 | fumarylacetoacetate hydrolase domain-containing protein 2 | IPR011234 : Fumarylacetoacetase |
| MGG05327.6 | 15.01 | 0.15 | hypothetical protein | No defined Interpro term |
| MGG06832.6 | 16.48 | 0.15 | conserved hypothetical protein | IPR001138: Fungal transcriptional regulatory protein, N-terminal |
| MGG06323.6 | 4.94 | 0.16 | solute carrier family 35 member E3 | IPR004853: Protein of unknown function DUF250 |
| MGG04191.6 | 5.01 | 0.16 | heat shock protein SSC1 | IPR001023: Heat shock protein Hsp70, IPR012725: Chaperone DnaK |
| MGG08917.6 | 6.59 | 0.16 | conserved hypothetical protein | IPR001138: Fungal transcriptional regulatory protein, N-terminal |
| MGG08941.6 | 6.66 | 0.16 | hypothetical protein | IPR011058: Cyanovirin-N |
| MGG10277.6 | 8.86 | 0.16 | brefeldin A resistance protein | IPR013525 : ABC-2 type transporter |
| MGG03336.6 | 9.8 | 0.16 | LEA domain-containing protein | No defined Interpro term |
| MGG08926.6 | 11.14 | 0.16 | conserved hypothetical protein | No defined Interpro term |
| MGG03597.6 | 4.08 | 0.17 | conserved hypothetical protein |  |
| MGG08122.6 | 9.51 | 0.17 | DNA replication licensing factor mcm2 | IPR001208: DNA-dependent ATPase MCM, IPR008045: MCM protein 2 |
| MGG03482.6 | 11.16 | 0.17 | hypothetical protein | No defined Interpro term |
| MGG05574.6 | 13.99 | 0.17 | conserved hypothetical protein | IPR006076: FAD dependent oxidoreductase |
| MGG01102.6 | 4.31 | 0.18 | ornithine carbamoyltransferase | IPR002292: Ornithine carbamoyltransferase |
| MGG05295.6 | 5.35 | 0.18 | conserved hypothetical protein | No defined Interpro term |
| MGG02775.6 | 8.28 | 0.18 | conserved hypothetical protein | IPR007087: Zinc finger, C2H2-type |
| MGG14825.6 | 4.04 | 0.19 | hypothetical protein | No defined Interpro term |
| MGG01883.6 | 4.67 | 0.19 | conserved hypothetical protein | IPR011701: Major facilitator superfamily MFS-1, IPR016196: Major facilitator superfamily, general substrate transporter |
| MGG03694.6 | 6.46 | 0.19 | beclin-1 | IPR007243: Autophagy protein 6 |
| MGG04925.6 | 9.97 | 0.19 | hypothetical protein | No defined Interpro term |
| MGG09055.6 | 23.21 | 0.19 | hypothetical protein | No defined Interpro term |
| MGG04880.6 | 5.63 | 0.2 | conserved hypothetical protein | No defined Interpro term |
| MGG13405.6 | 9.55 | 0.2 | geranylgeranyl pyrophosphate synthetase | IPR000092: Polyprenyl synthetase, IPR008949: Terpenoid synthase, |
| MGG05763.6 | 4.05 | 0.21 | stress responsive A/B barrel domain-containing protein | IPR011008: Dimeric alpha-beta barrel, IPR013097: Stress responsive alpha-beta barrel |
| MGG14006.6 | 4.47 | 0.21 | hypothetical protein | No defined Interpro term |
| MGG12598.6 | 4.89 | 0.21 | pantothenate kinase | IPR004567: Eukaryotic pantothenate kinase, IPR011602: Fumble |
| MGG04575.6 | 11.89 | 0.21 | conserved hypothetical protein | No defined Interpro term |
| MGG09533.6 | 4.99 | 0.22 | hypothetical protein | No defined Interpro term |
| MGG03238.6 | 10.82 | 0.22 | zinc finger protein ZPR1 | IPR004457: Zinc finger, ZPR1-type |
| MGG04353.6 | 4.98 | 0.23 | hypothetical protein | No defined Interpro term |
| MGG05670.6 | 8.46 | 0.23 | conserved hypothetical protein |  |
| MGG03369.6 | 13.72 | 0.23 | conserved hypothetical protein | IPR011038: Calycin-like, IPR012674 : Calycin |
| MGG02339.6 | 27.29 | 0.23 | hypothetical protein | IPR011058: Cyanovirin-N |
| MGG14716.6 | 5.04 | 0.24 | hypothetical protein | No defined Interpro term |
| MGG00655.6 | 5.4 | 0.24 | peroxisomal biogenesis factor 2 | IPR001841: Zinc finger, RING-type, IPR006845: Pex, N-terminal, |
| MGG06877.6 | 10.84 | 0.24 | DUF618 domain-containing protein | IPR006569: Regulation of nuclear pre-mRNA protein, IPR006903: Protein of unknown function DUF618 |
| MGG08111.6 | 3.98 | 0.25 | conserved hypothetical protein | No defined Interpro term |

^a^ Induction ratios from the microarray analysis are calculated as the expression in conidiating mycelia divided by non-conidiating mycelia of the wild-type.

^b^ Reduction ratios from the microarray analysis are calculated as the expression in the *ΔMohox2* mutant divided by the wild-type during conidiation.
